# Supplementary material for: Human contributions to global soundscapes are less predictable than the acoustic rhythms of wildlife
Source: Nat Ecol Evol. 2025 Jul 9;9(9):1585–98. doi: 10.1038/s41559-025-02786-5 (PMC12420389; doi:10.1038/s41559-025-02786-5)
Supplement: Supplementary file 1 — Supplementary Figs. 1–62 and Discussion. [file 41559_2025_2786_MOESM1_ESM.pdf]

nature ecology & evolution

Supplementary information https://doi.org/10.1038/s41559-025-02786-5

Human contributions to global soundscapes are less predictable than the acoustic rhythms of wildlife

In the format provided by the authors and unedited

Supplementary material for Somervuo et al.
Less predictable global rhythms in human than wildlife contributions to soundscapes

The current analyses are based on a total of 1,484,181 one-minute recordings from 139 different sites across six continents. These massive amounts of audio data can naturally be characterized from a wealth of different perspectives. In our study, we have chosen to focus on 15 “soundscape indices”, which are defined and discussed in the Main Text and Methods. In the main text of our article, we have brought out higher-level patterns of variation, there focusing on differences between the general categories of biophony, geophony and anthrophony. At the same time, we have pointed to differences in information content among individual indices, emphasizing that individual indices will be complementary rather than alternative in nature. To expose the more detailed patterns underlying our inference, and the patterns revealed by individual indices, we here provide the full set of results across all metrics addressed. We have structured this extensive Supplementary Material as follows:

In Section 1 (Seasonal and diurnal patterns in soundscape indices), we map all our sampling sites (Supplementary Figure 1), then illustrate the patterns revealed for each individual metric. The section starts with a verbal description of the patterns uncovered. Since diurnal patterns can be described with reference to either local, absolute time or time relative to local sunrise and sunset, the graphs are divided into two sections: 1.1 *Graphs relative to absolute time* and 1.2 *Graphs relative to the sun cycle*.

In Section 2 (Composition of sounds under index “Animal”), we take a critical look at the composition of sounds classified by YAMNet[1] as “animals”, aiming to resolve the fraction of vocalizations deriving from e.g. domestic animals and pets.

In Section 3 (Evaluating the effect of time representation on model predictions and inference), we address the question of whether different representations of time, i.e., absolute time of day (main text) or time relative to sun set and sun rise (Supplement, Section 3) affect our results.

In Section 4 (Correlations between different soundscape indices), we address the level of congruence among individual soundscape indices, in the form of a correlation plot.

In Section 5 (Validation of BirdNET detections), we evaluate congruence in the bird classifications reached by BirdNET[2] (as used in our analyses) and a human observer.

In Section 6 (Species and trait compositions in urban green spaces vs. more pristine sites), we ask whether urban green spaces and more pristine sites differ in terms of the trait composition of the local bird fauna.

The individual sections can be found on the following pages:

Contents

Section 1. Seasonal and diurnal patterns in soundscape indices 2

1.1 Graphs relative to absolute time 5

1.2 Graphs relative to the sun cycle. 32

Section 2. Composition of sounds under index “Animal” 59

Section 3. Evaluating the effect of time representation on model predictions and inference 60

Section 4. Correlations between different soundscape indices 65

Section 5. Validation of BirdNET detections 66

Section 6. Species and trait compositions in urban green spaces vs. more pristine sites 68
References 70

Section 1. Seasonal and diurnal patterns in soundscape indices

To reveal seasonal and diurnal patterns in each acoustic index addressed, we offer a series of plots showing predictions for each metric for the hour of the day and the day of the year for each site in the study. The colour code for each site is given in Supplementary Figure 1, which thus serves as a key to all Supplementary Figures 2-55. Since diurnal patterns can be described either with reference to local, absolute time or time relative to local sunrise and sunset, the graphs are divided into two sections: 1.1 *Graphs relative to absolute time* and 1.2 *Graphs relative to the sun cycle*.

The plots are grouped into four categories based on the type of variable that the model predicts. The four categories are 1) acoustic energy; 2) general acoustic indices ACI, ADI, Bio, H, NDSI (for definitions, see Fig. 1 in the main text); 3) acoustic events; and 4) bird species identified in the 1-min recording. For each variable, there are four panels A, B, C, D, which shows the model predictions for the hour of the day and the day of the year using both site-specific model and global model.

All methods in the four categories summarize the contents of the recording differently. The first category, acoustic energy, is the simplest and also the most robust. The second category (with five acoustic indices) offers slightly more sophisticated methods, e.g., by calculating entropy over time and frequency. We note that there are many acoustic indices, and that we have chosen to focus on a set of five commonly used ones (ACI, ADI, Bio, H, and NDSI). The two other categories (acoustic events and bird species identified) are perhaps the most complex indices. They characterize the contents of the recordings by providing them with a set of labels, thus describing the origin of the sound source (e.g., wind or rain or the bird species producing the vocalizations). For acoustic events, we use six classes from the Google AudioSet and for bird species identified, we draw on all the bird species that BirdNET 2.4 is able to recognize. How accurate a classification is achieved will depend on how well the data to be analyzed matches the data used in model training – and how well the model can generalize to unseen data. In the present work, the classification of acoustic events and the identification of bird species have implemented through convolutional neural networks (CNNs), using YAMNet[1] for acoustic events and BirdNET[2] for bird species identification. Below, we will examine the patterns resolved for each category of indices.

Category 1. Acoustic energy

Time series plots within this category are in Supplementary Figures 2-7 for absolute time and in Supplementary Figures 29-34 for time relative to sun cycle. For acoustic energy, we show time series predictions for low, middle, and high frequency bands using both maximum and mean energy within a 1-minute recording as the response variable of the model. The global model (panels B and D) provides smoother time series compared to the site-specific models (panels A and C). In the global model the only information of each site is the latitude. For low frequency sounds (0-1 kHz), we note that the diurnal peak occurs during the middle of the day and that across space, there is more sound energy at southern sites (panel B: green, yellow, and red) compared to northern sites (blue and magenta). Over seasons (day of the year, panel D), the sound energy is highest at sites near the Equator (green). For mid-frequency sounds (1-10 kHz), the daily patterns (panel B) show two peaks, one in the late morning and the other in the evening. These peaks are clearest for mid-latitudinal and southern sites (green and yellow). Acoustic energy is higher at southern sites compared to northern sites. Mid-frequency sounds show clear seasonal patterns, with the peaks occurring during the local spring/early summer (with half-a-year delay between northern and southern hemispheres). High-frequency sounds (10-20 kHz) are more abundant at southern sites compared

to northern sites. At southern sites (red), the seasonal peak occurs at the beginning of the year. Near the Equator, the peak occurs during the autumn (panel D).

Category 2. General acoustic indices

Time series plots within this category are in Supplementary Figures 8-12 for absolute time and in Supplementary Figures 35-39 for time relative to sun cycle. For general acoustic indices, we show time series predictions for acoustic indices ACI, ADI, Bio, H, and NDSI. Of these, all indices show differences between northern and southern sites: ACI and ADI show single peaks during the day and ADI shows seasonal patterns. The Bio index shows pattern somewhat similar to those in mid-frequency sounds (see above), with two peaks during the day and clear seasonal patterns. NDSI reveals seasonal patterns with half-a-year difference between peaks at northern and southern sites. H shows bimodal diel patterning (panel B) and a strong seasonal pattern for southern sites (yellow and red, panel D).

Category 3. Acoustic events

Time series plots for this category are in Supplementary Figures 13-24 for absolute time and in Supplementary Figures 40-51 for time relative to sun cycle. In terms of acoustic events, we show time series predictions for six AudioSet classes: Speech, Animal, Vehicle, Silence, Wind, and Rain. In each case, we resolve both maximum and mean probabilities within each 1-min recording. Silence is mostly detected at northern sites. Southern sites exhibit a seasonal peak during the summer (panel D). For northern sites (blue and magenta), the seasonal peak occurs towards the beginning/end of the year. Animal detections show both daily and seasonal patterning. The daily pattern is bimodal, with peaks in the morning and evening. Counts of Animal detections are greatest at southern sites and lowest at northern sites. At southern sites (red and yellow), the seasonal peak occurs during the beginning/end of the year, whereas at central and northern sites (green and blue), the peak occurs during the spring. Wind detections are greatest in northern sites. For daily pattern, WindMax shows a clearer peak during the day compared to WindMean. Both indices reveal seasonal patterns and differences between northern and southern sites (panel D). For rain, RainMax shows a clearer peak during the day compared to RainMean (panel B). Yet, for both inidces, the frequency of rain detections is greater near the Equator (green) than at other sites. Rain detections show seasonal trends: at southern sites the peaks occur during the first half of the year (and slightly beyond that), whereas at low latitudes and at northern sites, the peaks occur during the autumn and end of the year. The daily pattern in Vehicle detections shows two peaks at low latitudes (green), where detection counts are also greatest. The seasonal patterns in Vehicle detections are weak. Detections of Speech peak during the middle of the day, but lack any clear seasonal pattern. The amount of speech is lowest at northern sites (blue and magenta).

Category 4. Bird species identified

Time series plots for this category are in Supplementary Figures 25-28 for absolute time and in Supplementary Figures 52-55 for time relative to sun cycle.
With respect to Bird species identified, we show predictions for bird species richness and abundance using two different thresholds (0.3 and 0.8) for BirdNET confidence values. Diel variation in both species richness and abundance show a strong bimodal pattern (panel B). The stronger peak occurs during the morning and the smaller peak occurs in the evening. In seasonal pattern, the peak occurs in the local spring/early summer for sites in the northern hemisphere (blue and green); in the southern hemisphere, it occurs half a year later during the local spring/summer (yellow and red). Curves based on different thresholds for BirdNET detections exhibit similar shapes, with differences only in the absolute number of counts.

Compared with time series plots using absolute time, although general patterns are highly similar using both representations of time, in the plots where the time is relative to sun cycle, the peaks in the morning and evening are better aligned across all sites of different latitudes.

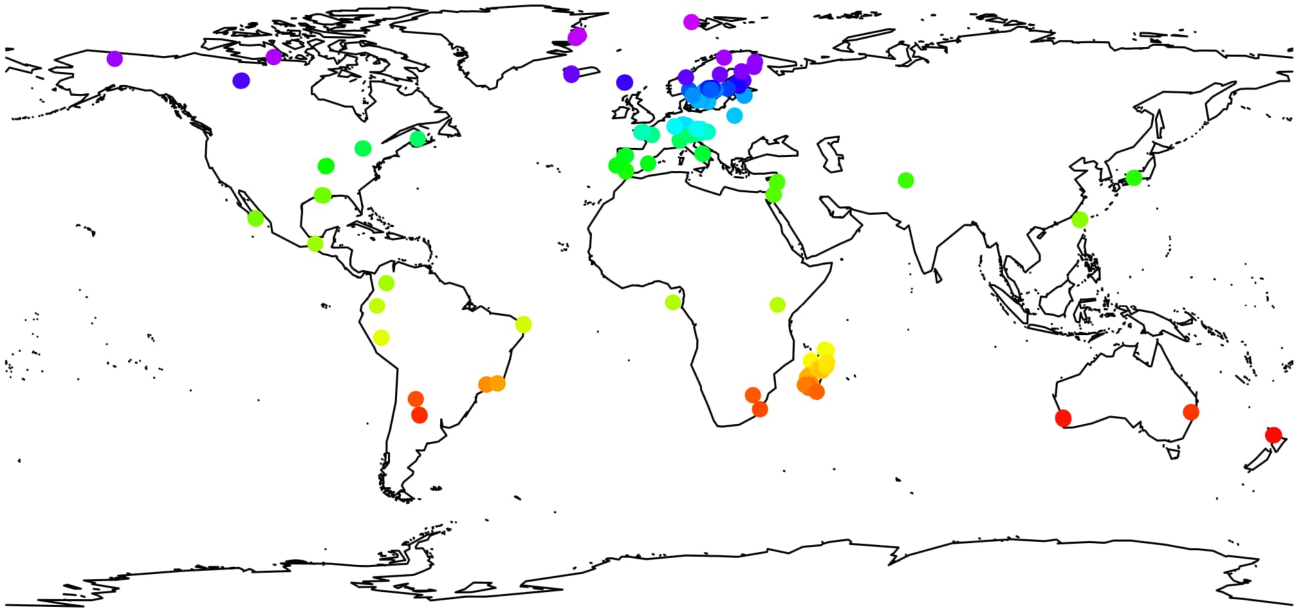

Supplementary Figure 1. Recording sites colour-coded by latitude.

This map serves as a legend for Supplementary Figures 2-55.

1.1 Graphs relative to absolute time

In all graphs below (Supplementary Figures 2-28), we show predictions from models where the local hour is represented by the local absolute hour.

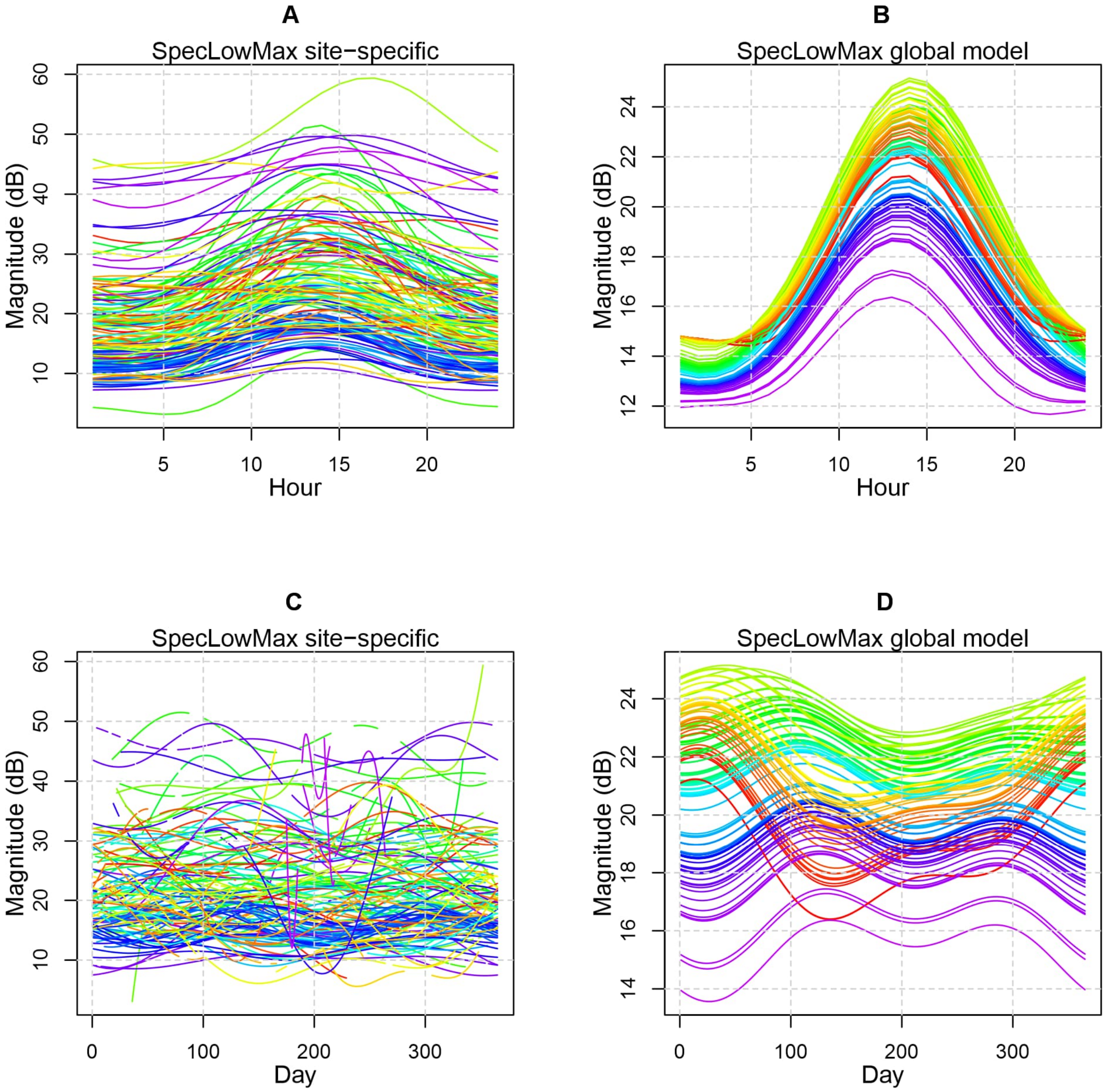

Supplementary Figure 2. Predictions of SpecLowMax for the hour of the day (A, B) and the day of the year (C, D) for all sites in the study. Here, time is represented by the absolute hour. Site-specific models are displayed on the left (A, C), with global model predictions on the right (B, D). Each site is color-coded according to the map in Supplementary Figure 1. All sites included have data from at least 100 days except for 3 sites for which the accessible field season is very short (locations in the extreme north).

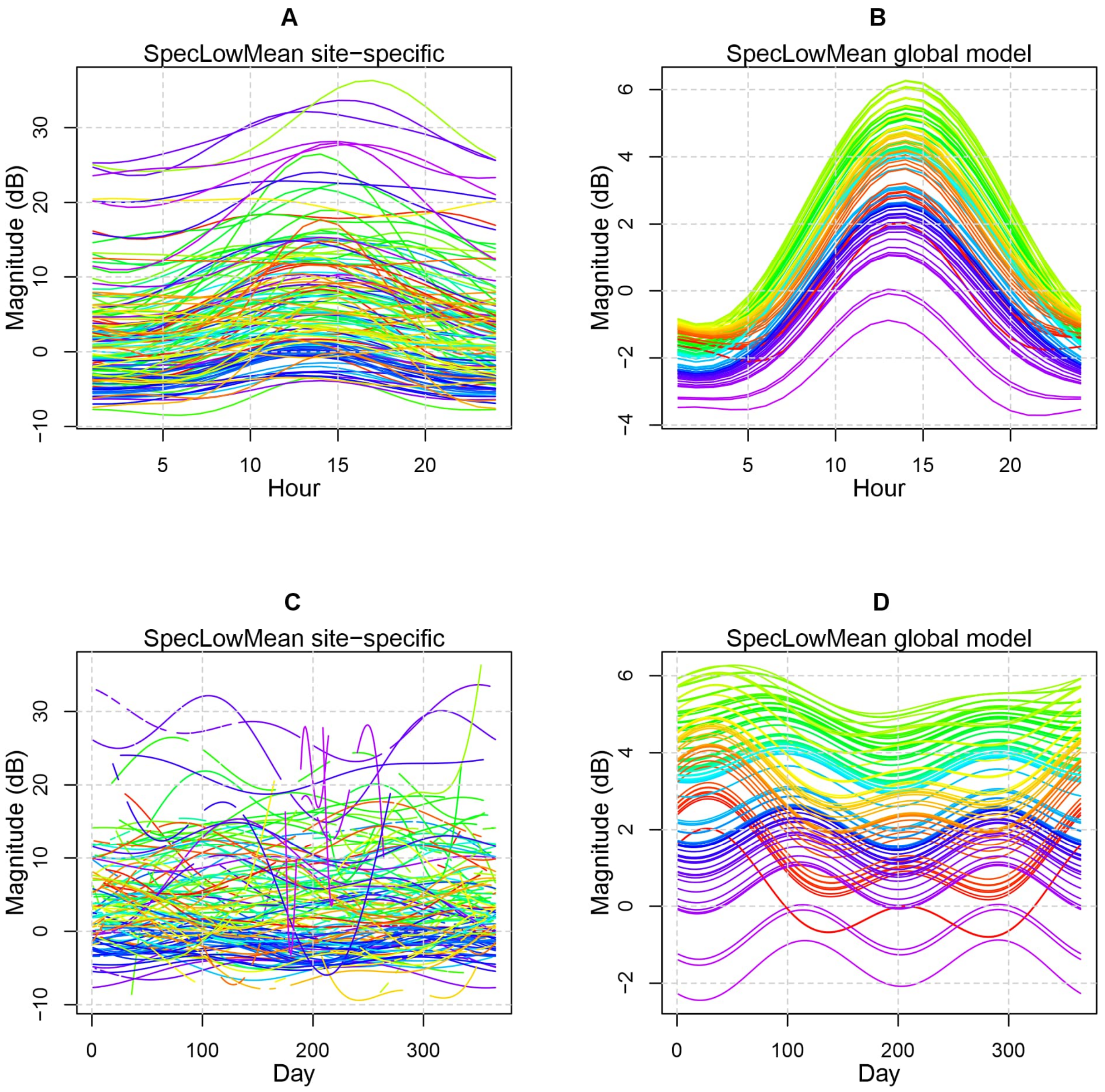

Supplementary Figure 3. Predictions of SpecLowMean for the hour of the day (A, B) and the day of the year (C, D) for all sites in the study. Here, time is represented by the absolute hour. Site-specific models are displayed on the left (A, C), with global model predictions on the right (B, D). Each site is color-coded according to the map in Supplementary Figure 1. All sites included have data from at least 100 days except for 3 sites for which the accessible field season is very short (locations in the extreme north).

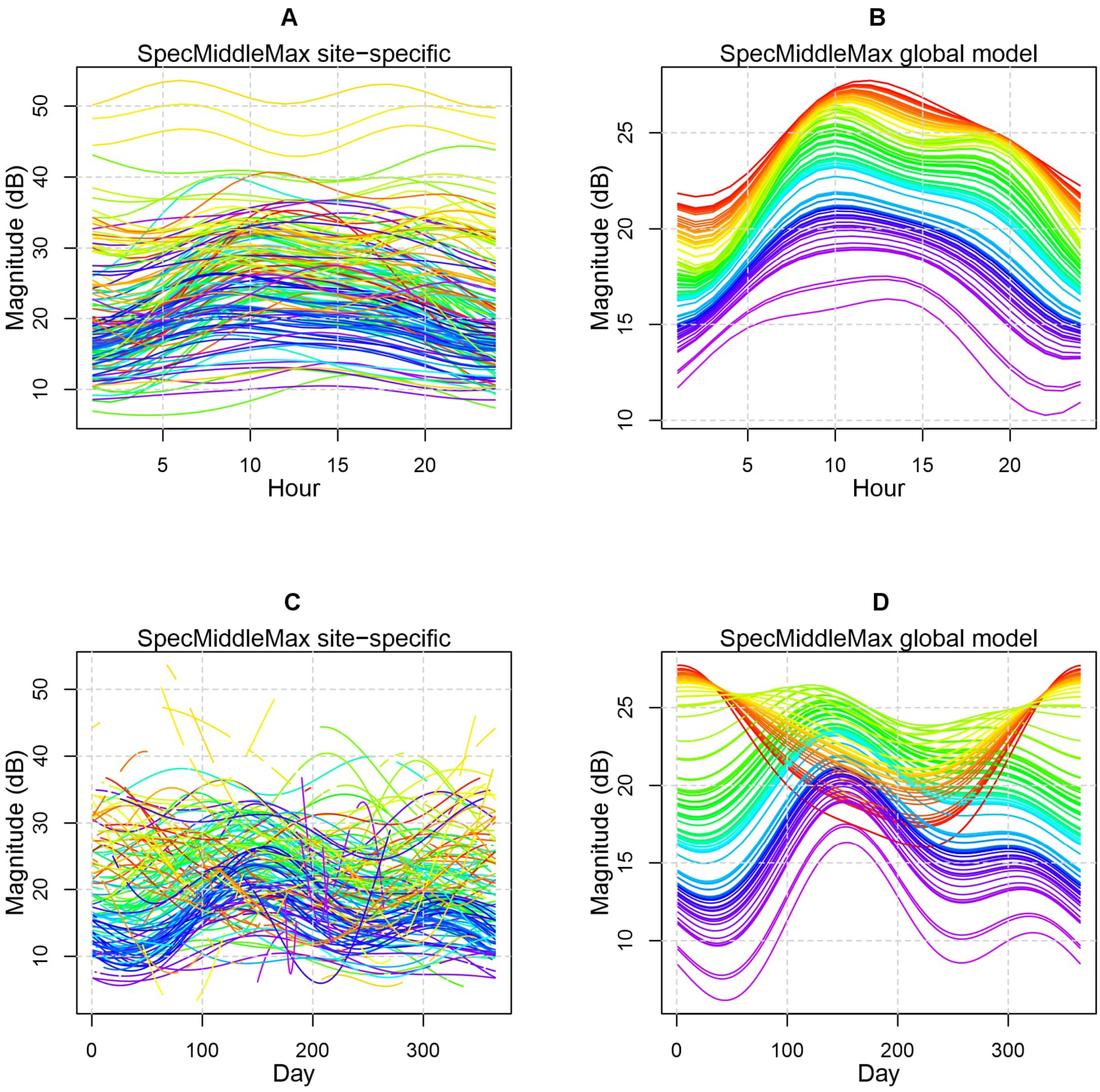

Supplementary Figure 4. Predictions of SpecMiddleMax for the hour of the day (A, B) and the day of the year (C, D) for all sites in the study. Here, time is represented by the absolute hour. Site-specific models are displayed on the left (A, C), with global model predictions on the right (B, D). Each site is color-coded according to the map in Supplementary Figure 1. All sites included have data from at least 100 days except for 3 sites for which the accessible field season is very short (locations in the extreme north).

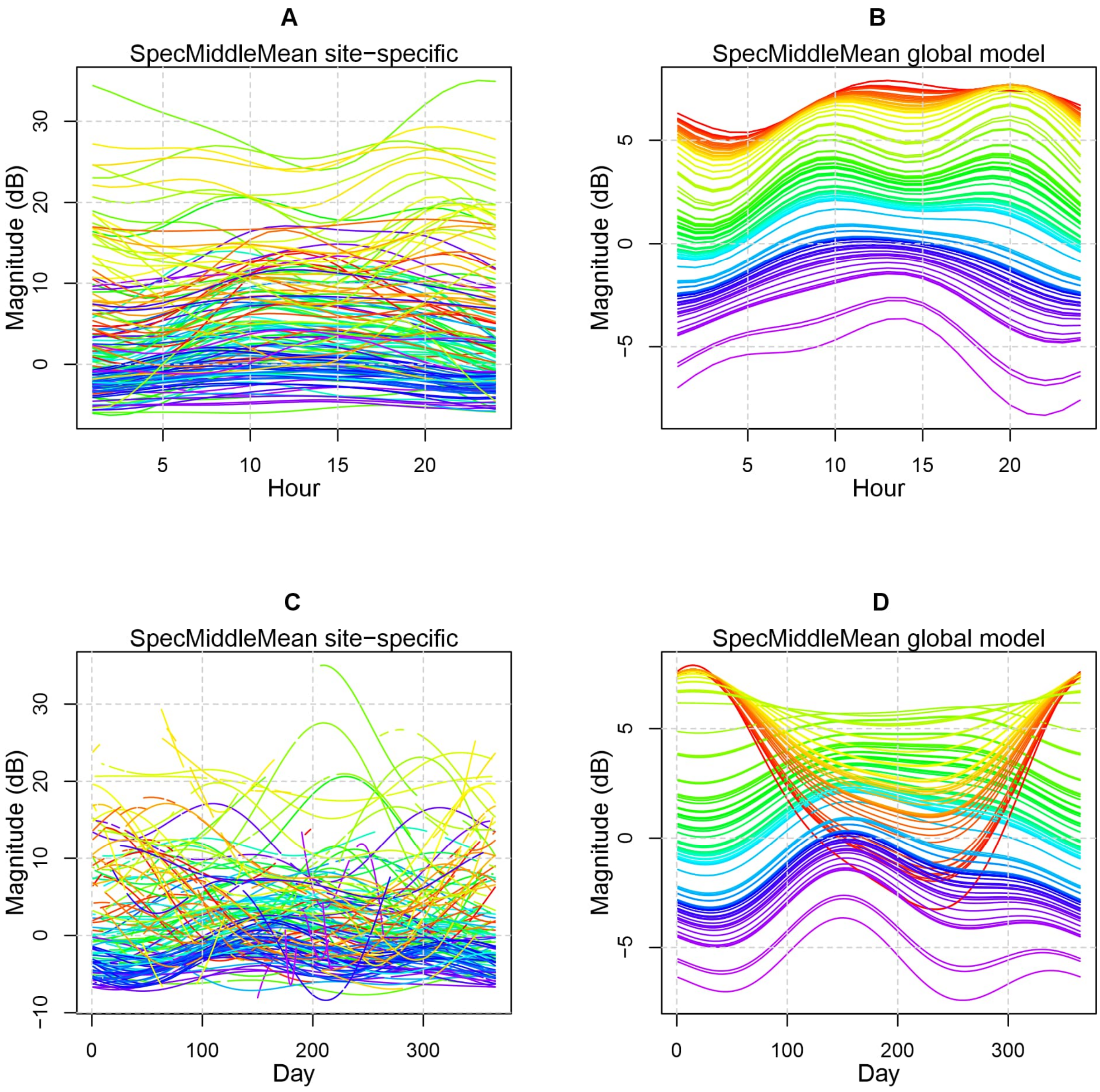

Supplementary Figure 5. Predictions of SpecMiddleMean for the hour of the day (A, B) and the day of the year (C, D) for all sites in the study. Here, time is represented by the absolute hour. Site-specific models are displayed on the left (A, C), with global model predictions on the right (B, D). Each site is color-coded according to the map in Supplementary Figure 1. All sites included have data from at least 100 days except for 3 sites for which the accessible field season is very short (locations in the extreme north).

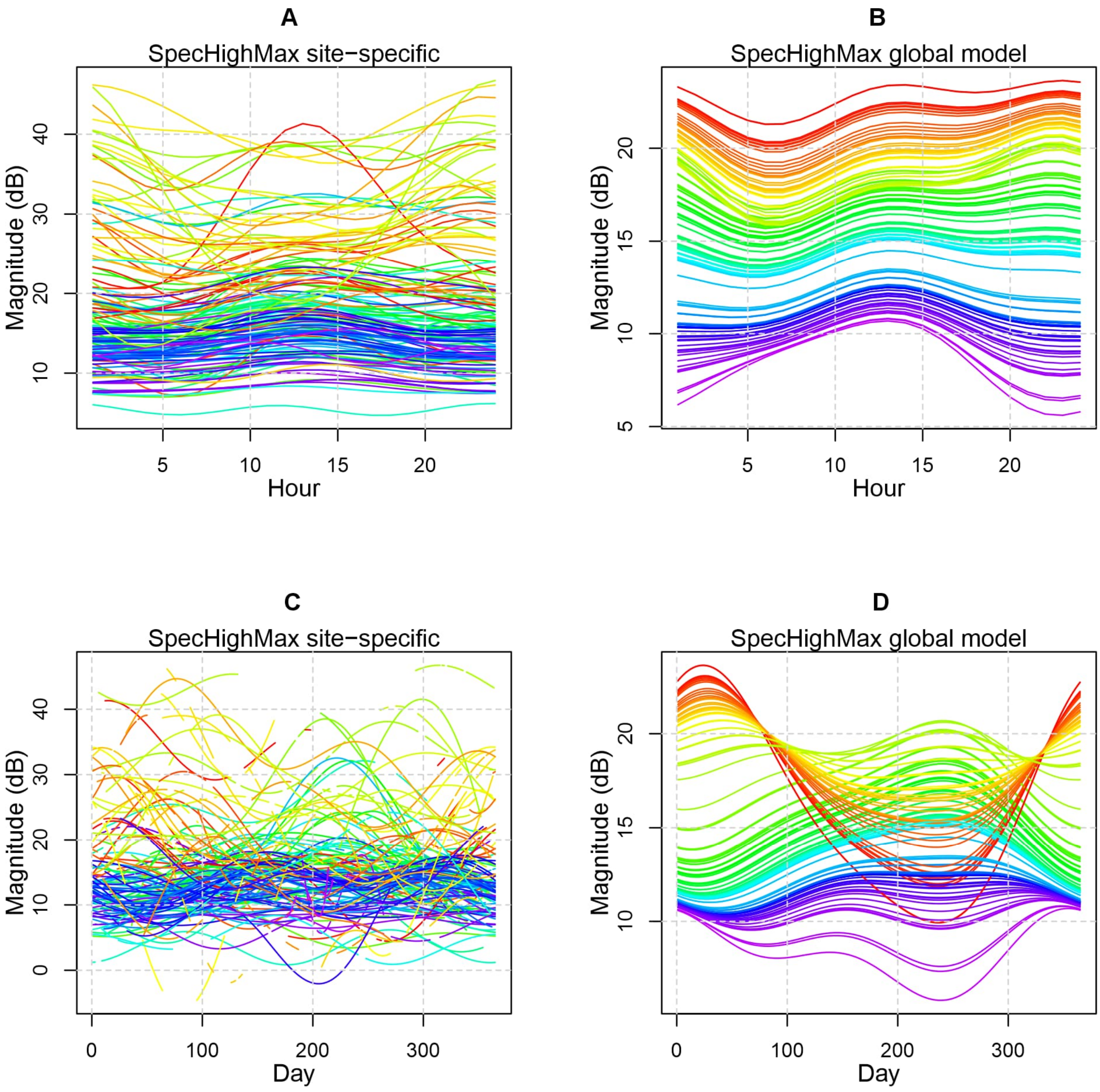

Supplementary Figure 6. Predictions of SpecHighMax for the hour of the day (A, B) and the day of the year (C, D) for all sites in the study. Here, time is represented by the absolute hour. Site-specific models are displayed on the left (A, C), with global model predictions on the right (B, D). Each site is color-coded according to the map in Supplementary Figure 1. All sites included have data from at least 100 days except for 3 sites for which the accessible field season is very short (locations in the extreme north).

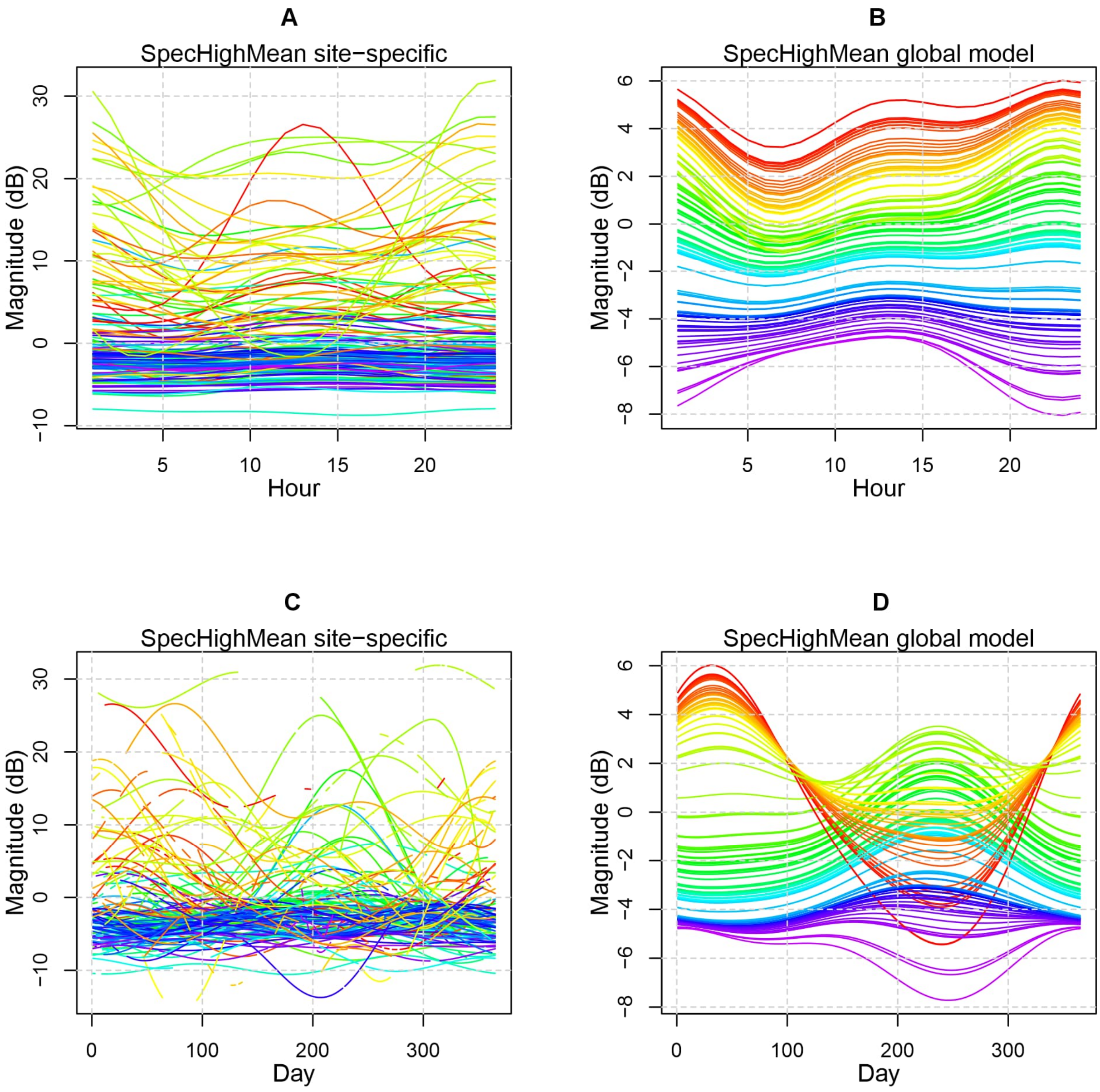

Supplementary Figure 7. Predictions of SpecHighMean for the hour of the day (A, B) and the day of the year (C, D) for all sites in the study. Here, time is represented by the absolute hour. Site-specific models are displayed on the left (A, C), with global model predictions on the right (B, D). Each site is color-coded according to the map in Supplementary Figure 1. All sites included have data from at least 100 days except for 3 sites for which the accessible field season is very short (locations in the extreme north).

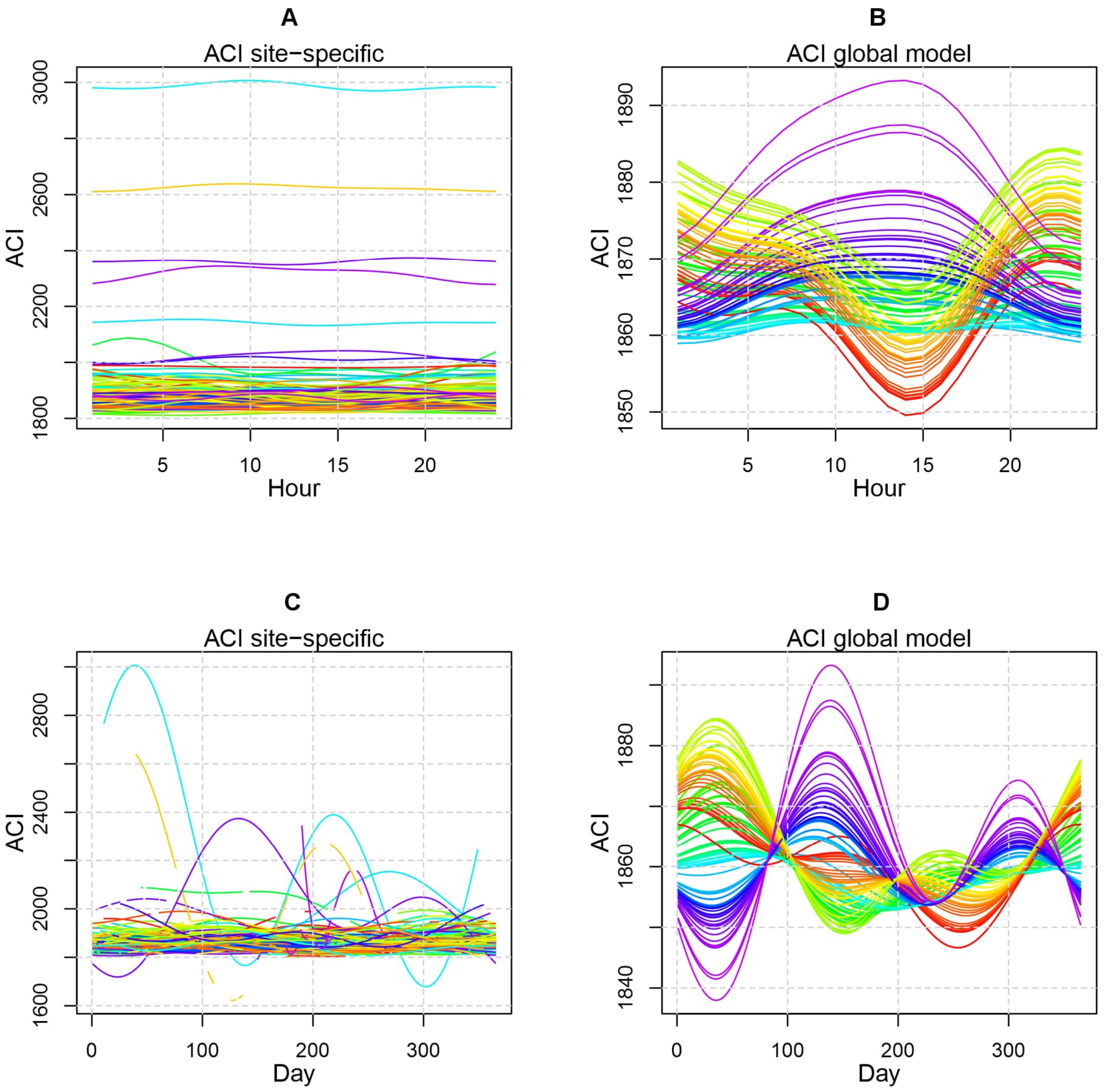

Supplementary Figure 8. Predictions of ACI for the hour of the day (A, B) and the day of the year (C, D) for all sites in the study. Here, time is represented by the absolute hour. Site-specific models are displayed on the left (A, C), with global model predictions on the right (B, D). Each site is color-coded according to the map in Supplementary Figure 1. All sites included have data from at least 100 days except for 3 sites for which the accessible field season is very short (locations in the extreme north).

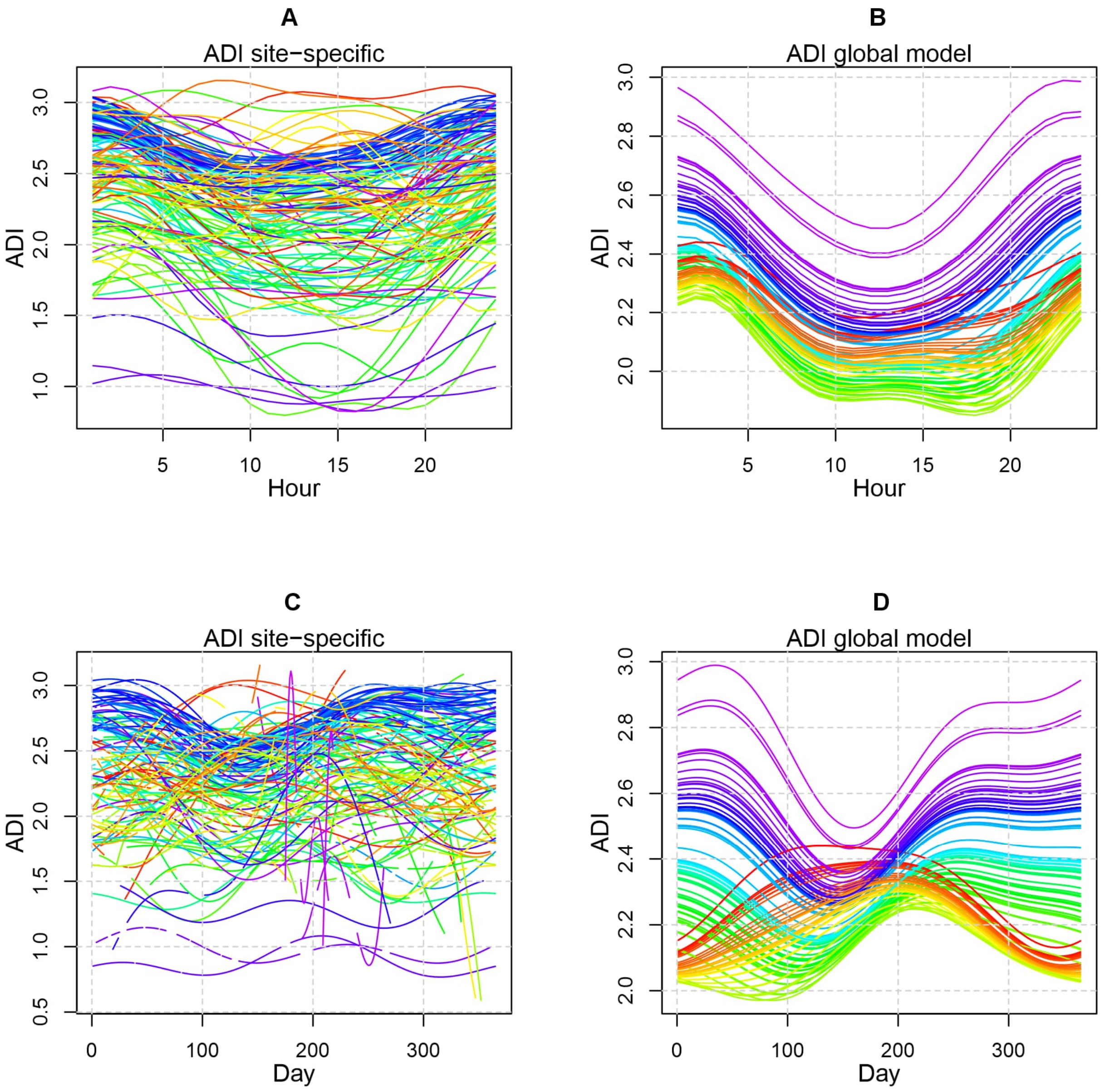

Supplementary Figure 9. Predictions of ADI for the hour of the day (A, B) and the day of the year (C, D) for all sites in the study. Here, time is represented by the absolute hour. Site-specific models are displayed on the left (A, C), with global model predictions on the right (B, D). Each site is color-coded according to the map in Supplementary Figure 1. All sites included have data from at least 100 days except for 3 sites for which the accessible field season is very short (locations in the extreme north).

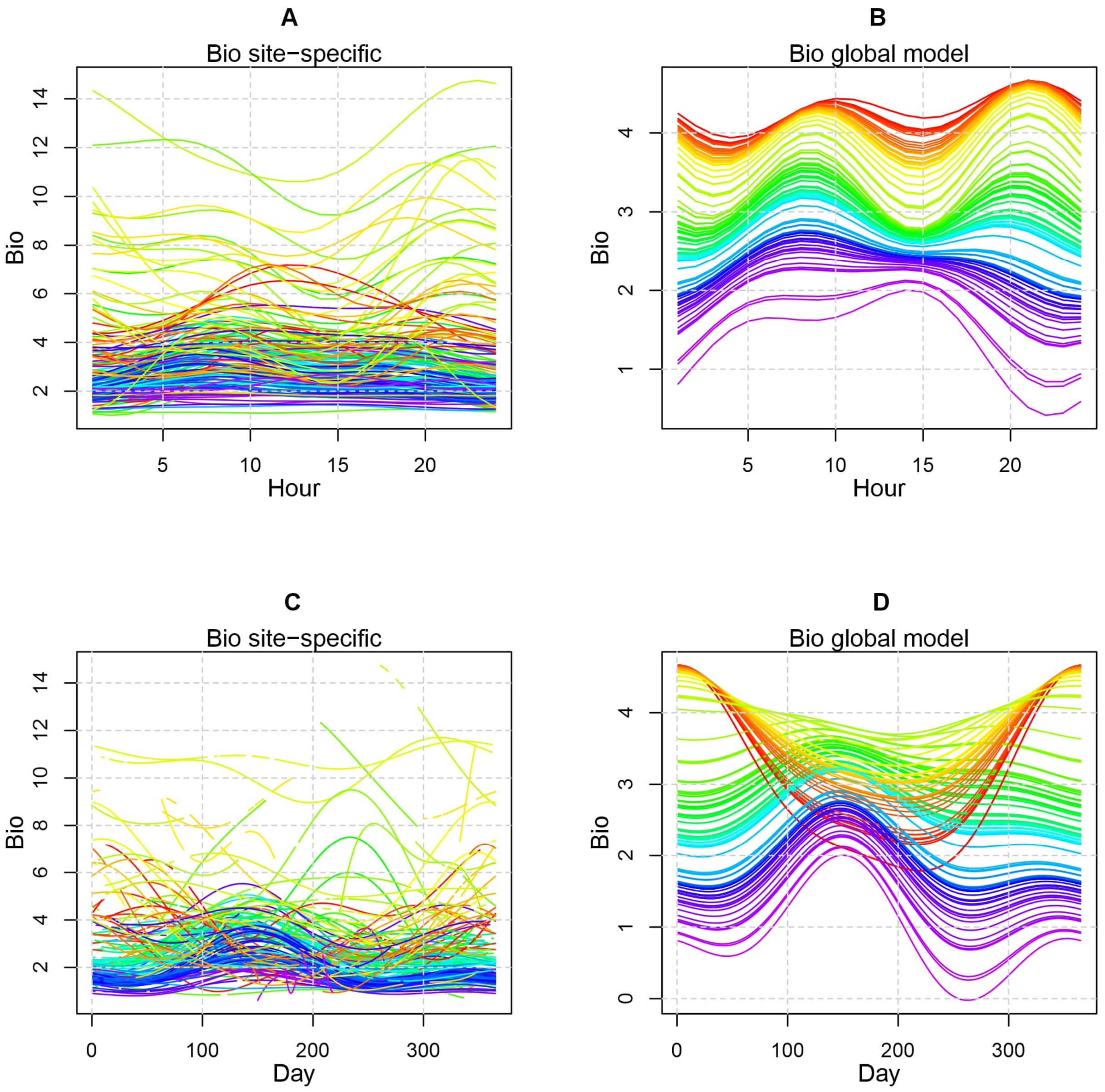

Supplementary Figure 10. Predictions of ADI for the hour of the day (A, B) and the day of the year (C, D) for all sites in the study. Here, time is represented by the absolute hour. Site-specific models are displayed on the left (A, C), with global model predictions on the right (B, D). Each site is color-coded according to the map in Supplementary Figure 1. All sites included have data from at least 100 days except for 3 sites for which the accessible field season is very short (locations in the extreme north).

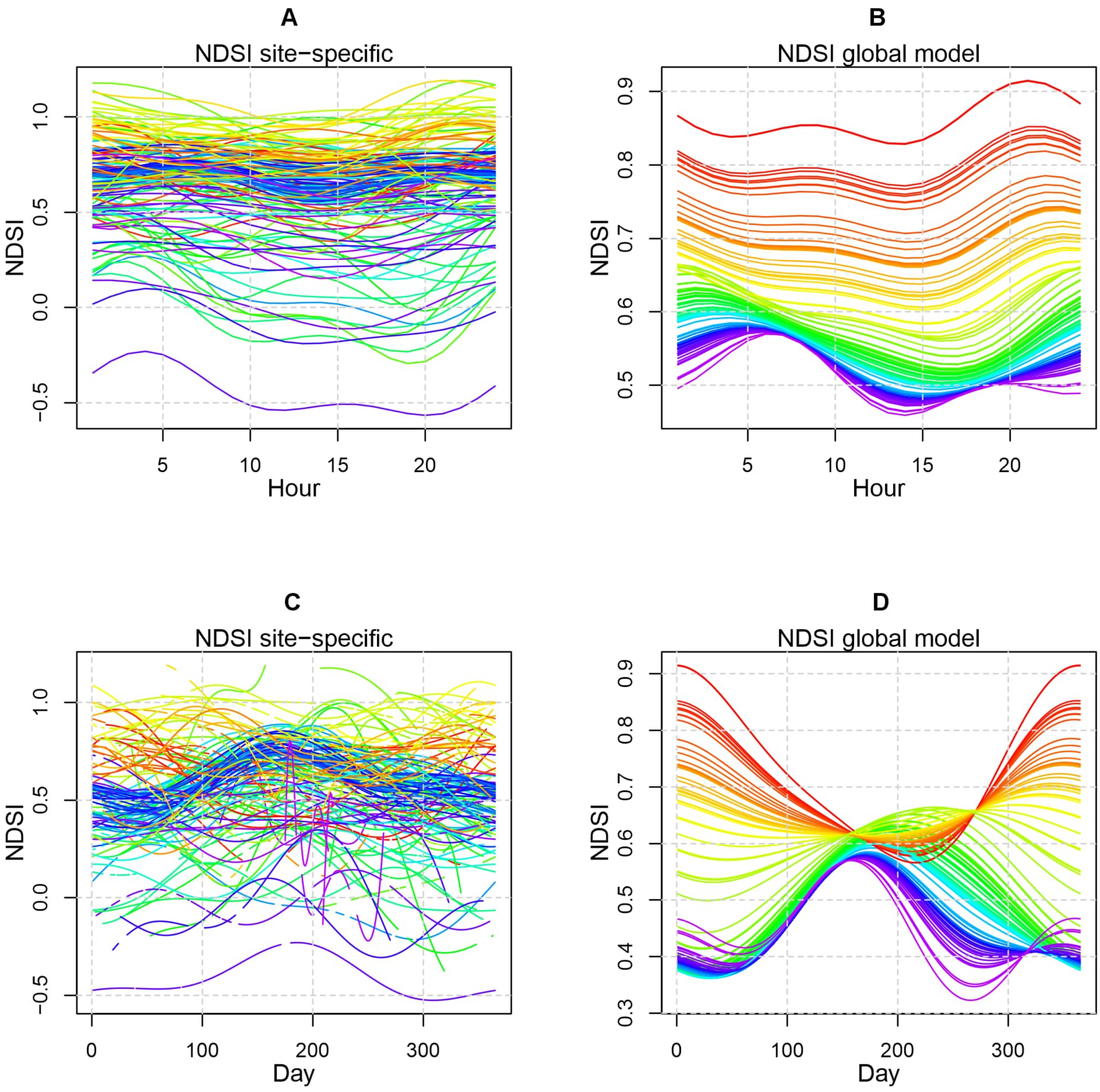

Supplementary Figure 11. Predictions of NDSI for the hour of the day (A, B) and the day of the year (C, D) for all sites in the study. Here, time is represented by the absolute hour. Site-specific models are displayed on the left (A, C), with global model predictions on the right (B, D). Each site is color-coded according to the map in Supplementary Figure 1. All sites included have data from at least 100 days except for 3 sites for which the accessible field season is very short (locations in the extreme north).

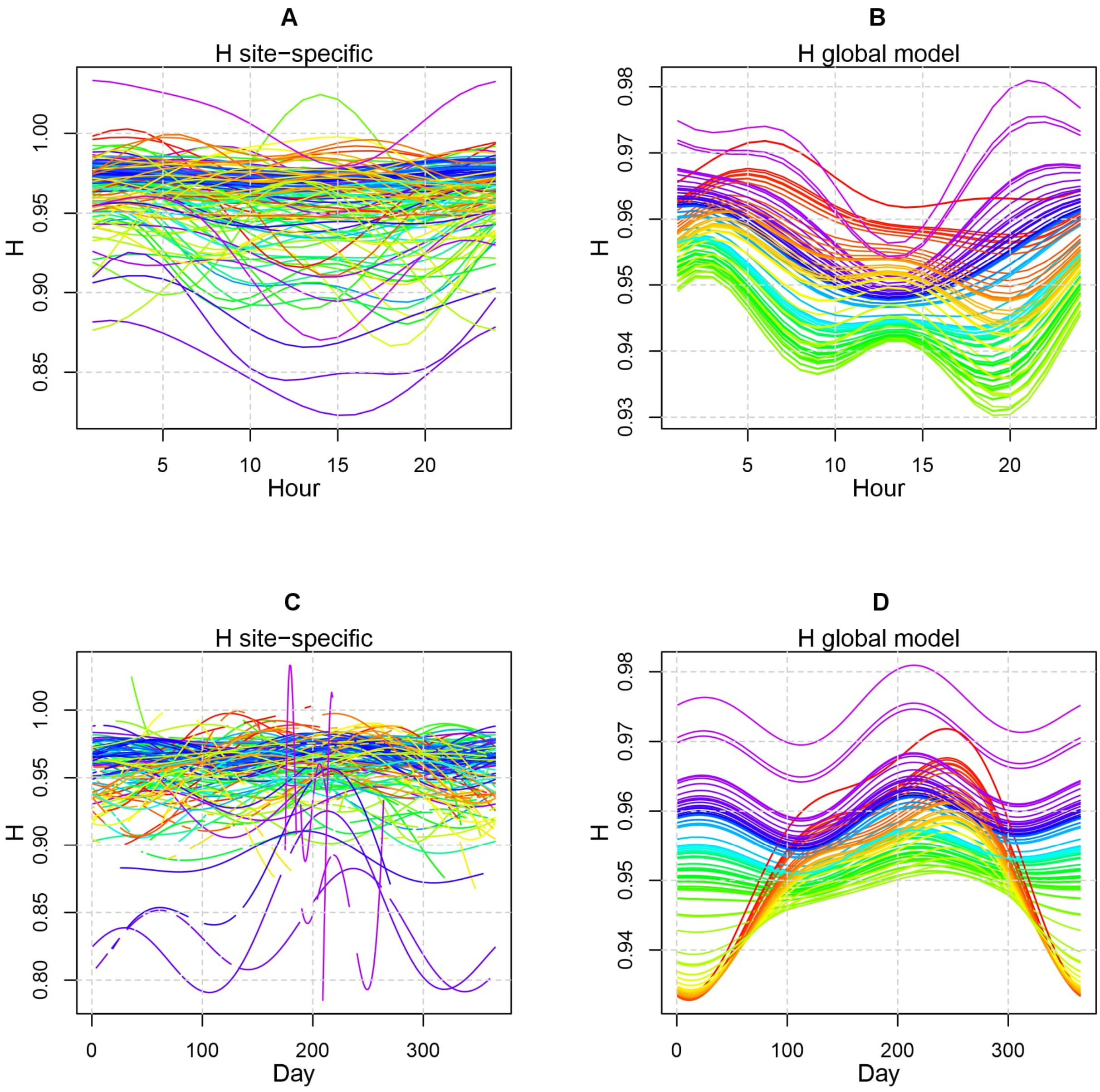

Supplementary Figure 12. Predictions of H for the hour of the day (A, B) and the day of the year (C, D) for all sites in the study. Here, time is represented by the absolute hour. Site-specific models are displayed on the left (A, C), with global model predictions on the right (B, D). Each site is color-coded according to the map in Supplementary Figure 1. All sites included have data from at least 100 days except for 3 sites for which the accessible field season is very short (locations in the extreme north).

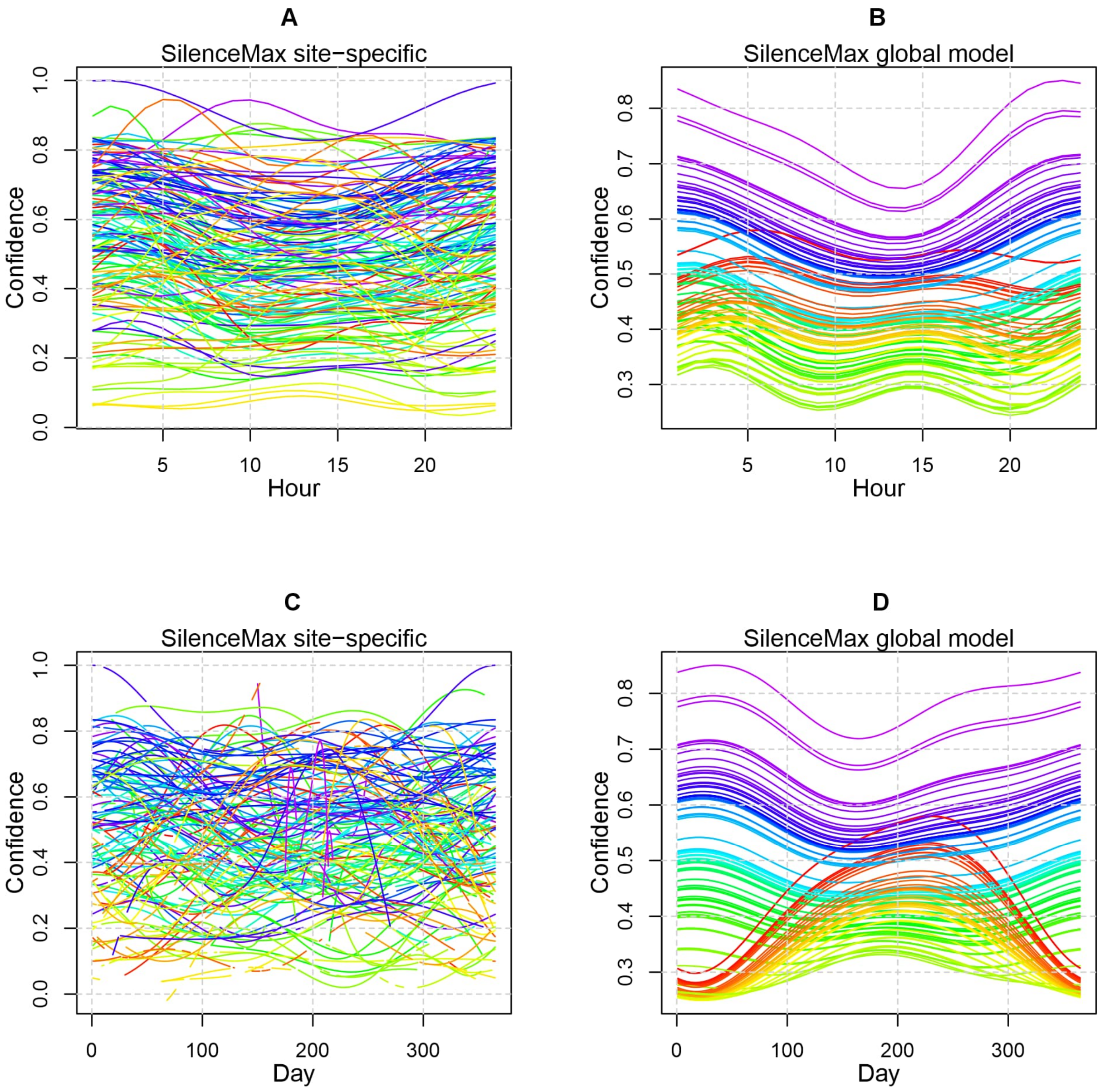

Supplementary Figure 13. Predictions of SilenceMax for the hour of the day (A, B) and the day of the year (C, D) for all sites in the study. Here, time is represented by the absolute hour. Site-specific models are displayed on the left (A, C), with global model predictions on the right (B, D). Each site is color-coded according to the map in Supplementary Figure 1. All sites included have data from at least 100 days except for 3 sites for which the accessible field season is very short (locations in the extreme north).

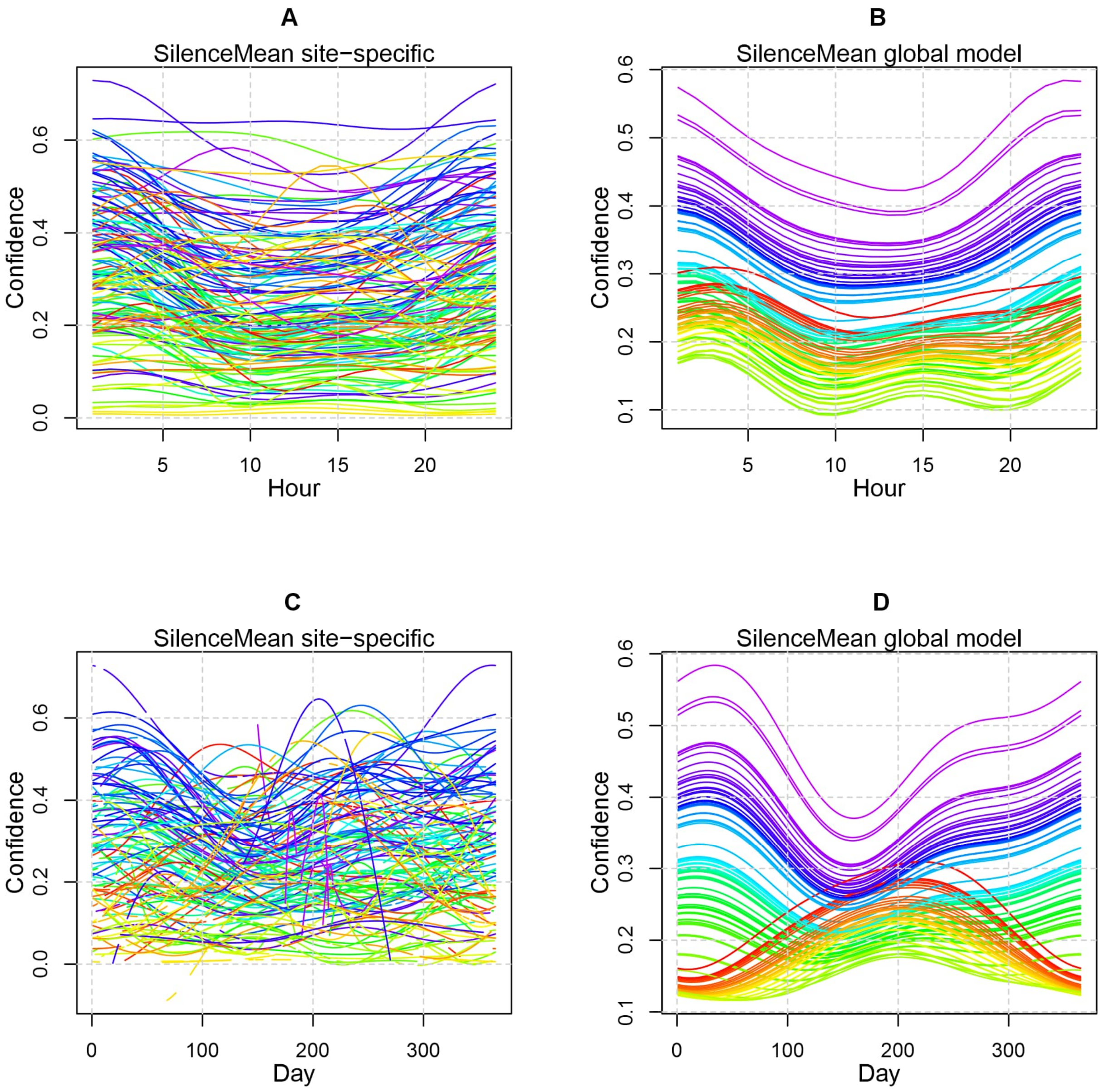

Supplementary Figure 14. Predictions of SilenceMean for the hour of the day (A, B) and the day of the year (C, D) for all sites in the study. Here, time is represented by the absolute hour. Site-specific models are displayed on the left (A, C), with global model predictions on the right (B, D). Each site is color-coded according to the map in Supplementary Figure 1. All sites included have data from at least 100 days except for 3 sites for which the accessible field season is very short (locations in the extreme north).

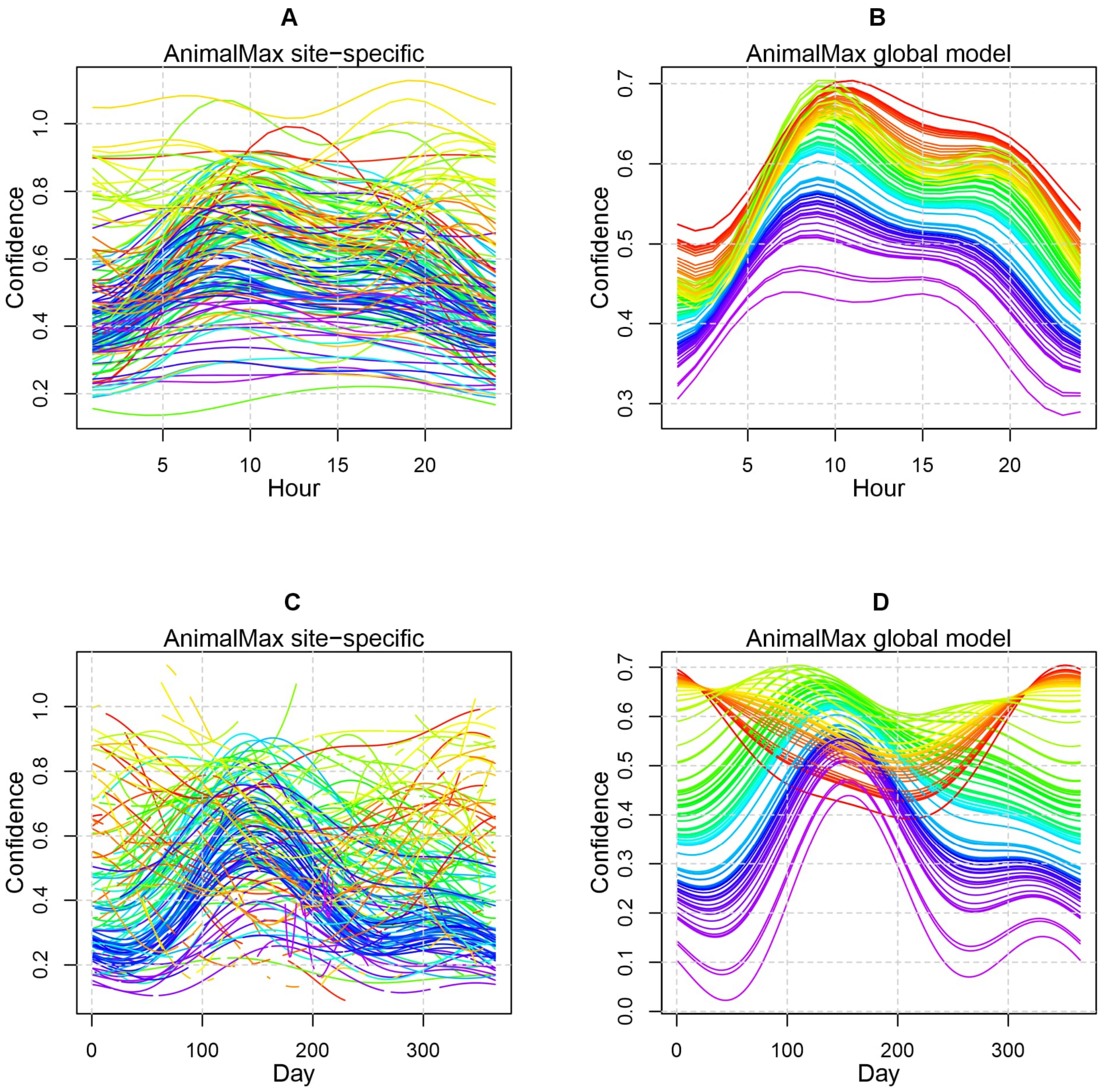

Supplementary Figure 15. Predictions of AnimalMax for the hour of the day (A, B) and the day of the year (C, D) for all sites in the study. Here, time is represented by the absolute hour. Site-specific models are displayed on the left (A, C), with global model predictions on the right (B, D). Each site is color-coded according to the map in Supplementary Figure 1. All sites included have data from at least 100 days except for 3 sites for which the accessible field season is very short (locations in the extreme north).

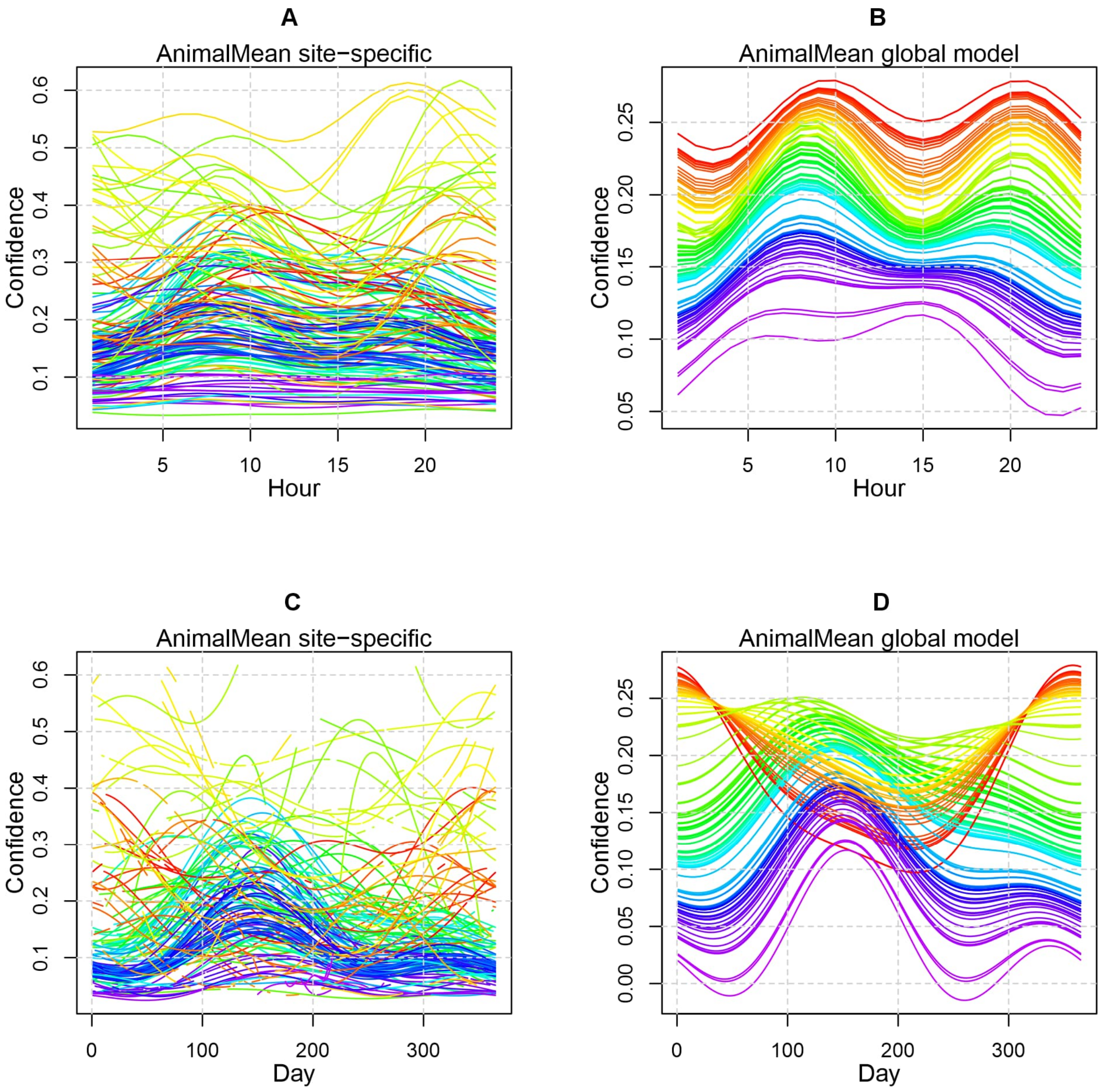

Supplementary Figure 16. Predictions of AnimalMean for the hour of the day (A, B) and the day of the year (C, D) for all sites in the study. Here, time is represented by the absolute hour. Site-specific models are displayed on the left (A, C), with global model predictions on the right (B, D). Each site is color-coded according to the map in Supplementary Figure 1. All sites included have data from at least 100 days except for 3 sites for which the accessible field season is very short (locations in the extreme north).

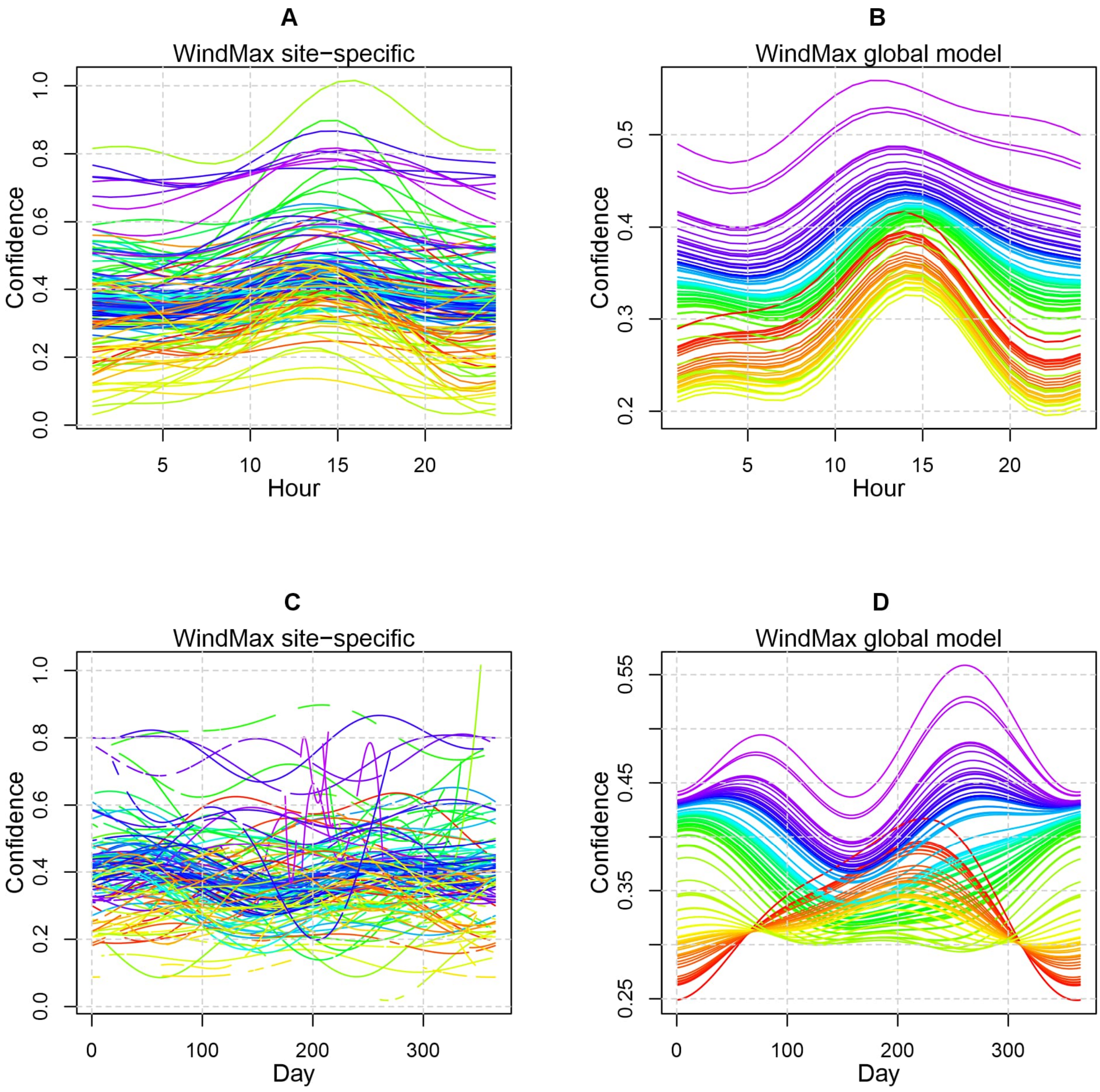

Supplementary Figure 17. Predictions of WindMax for the hour of the day (A, B) and the day of the year (C, D) for all sites in the study. Here, time is represented by the absolute hour. Site-specific models are displayed on the left (A, C), with global model predictions on the right (B, D). Each site is color-coded according to the map in Supplementary Figure 1. All sites included have data from at least 100 days except for 3 sites for which the accessible field season is very short (locations in the extreme north).

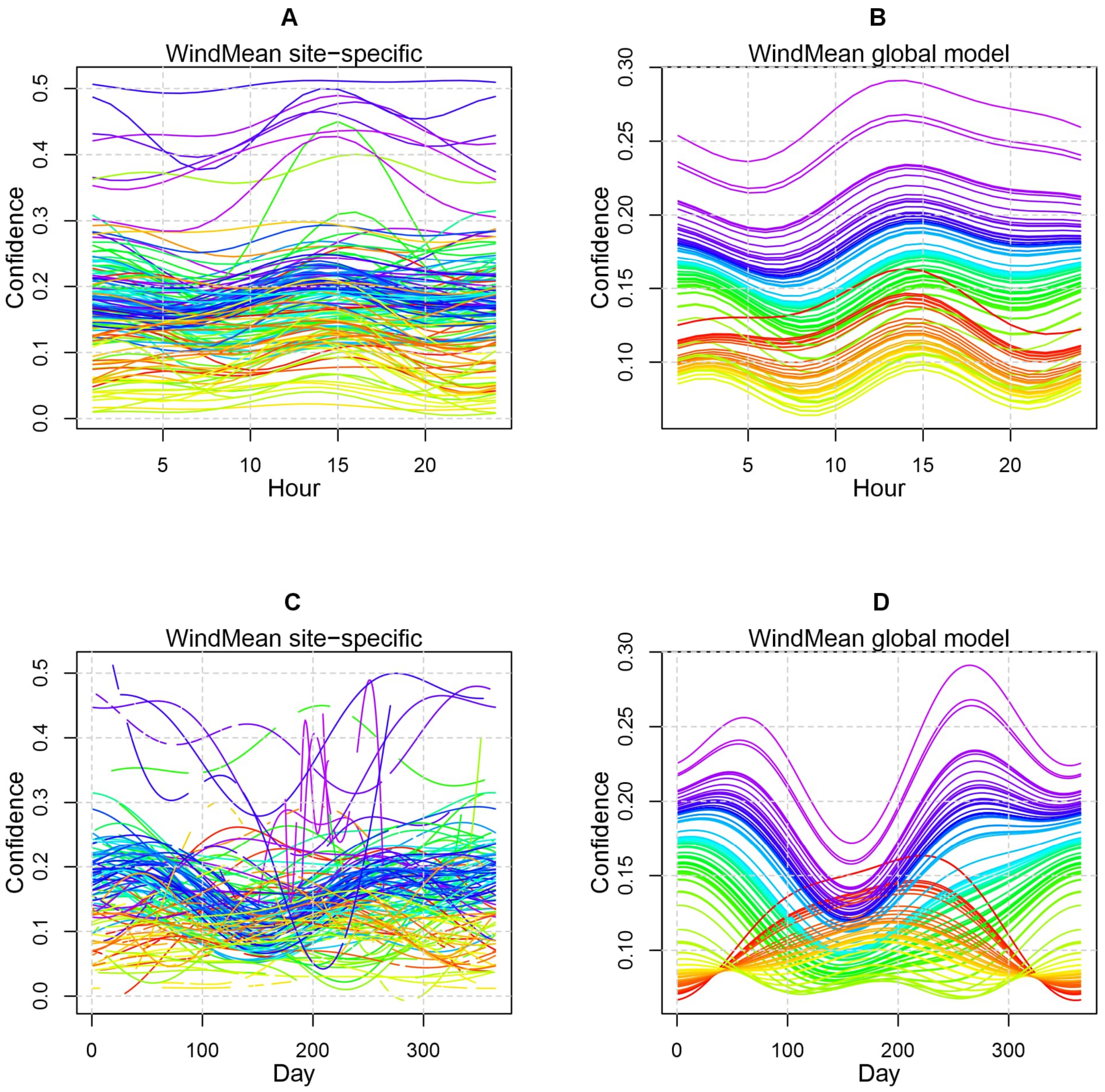

Supplementary Figure 18. Predictions of WindMean for the hour of the day (A, B) and the day of the year (C, D) for all sites in the study. Here, time is represented by the absolute hour. Site-specific models are displayed on the left (A, C), with global model predictions on the right (B, D). Each site is color-coded according to the map in Supplementary Figure 1. All sites included have data from at least 100 days except for 3 sites for which the accessible field season is very short (locations in the extreme north).

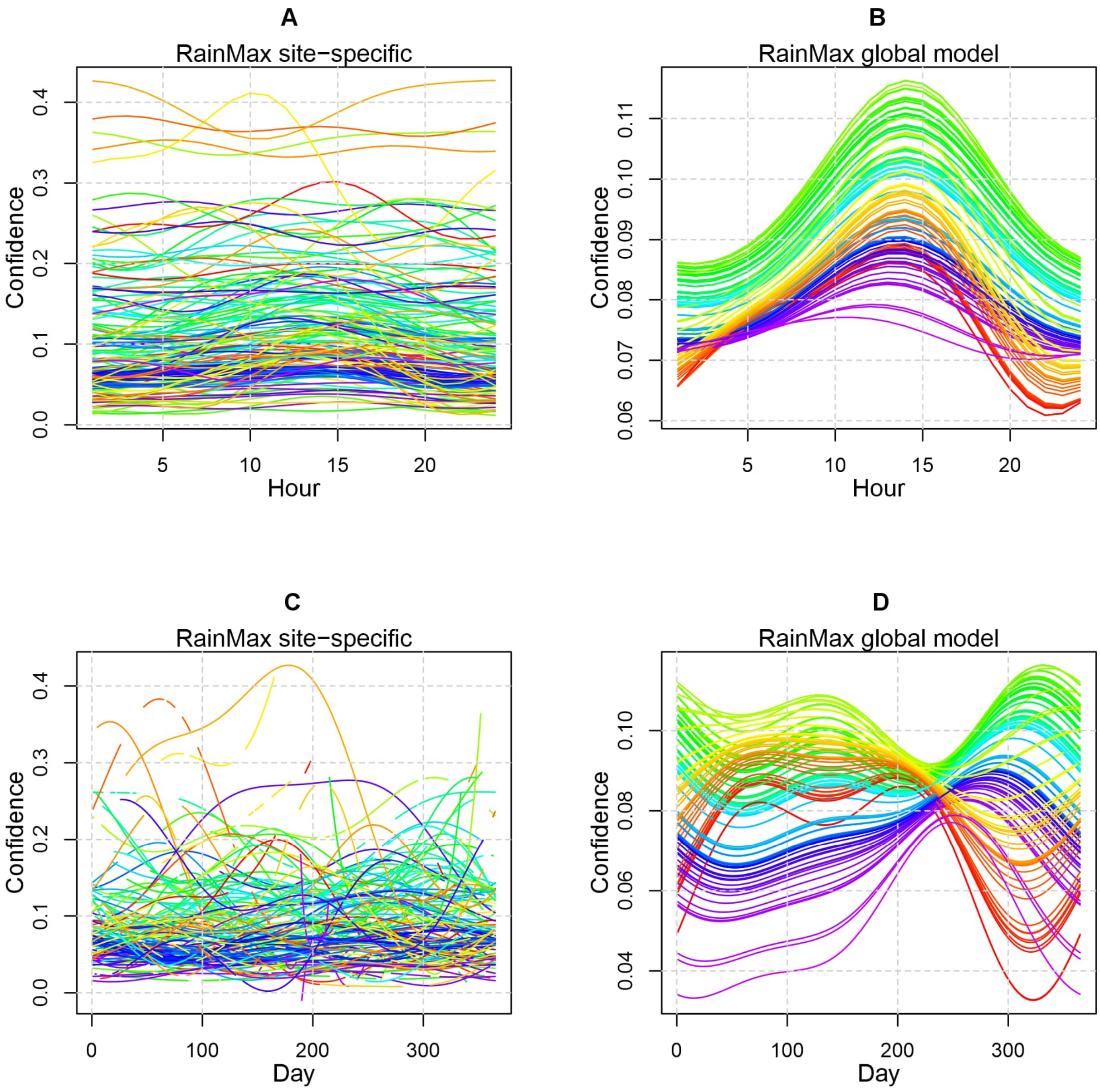

Supplementary Figure 19. Predictions of RainMax for the hour of the day (A, B) and the day of the year (C, D) for all sites in the study. Here, time is represented by the absolute hour. Site-specific models are displayed on the left (A, C), with global model predictions on the right (B, D). Each site is color-coded according to the map in Supplementary Figure 1. All sites included have data from at least 100 days except for 3 sites for which the accessible field season is very short (locations in the extreme north).

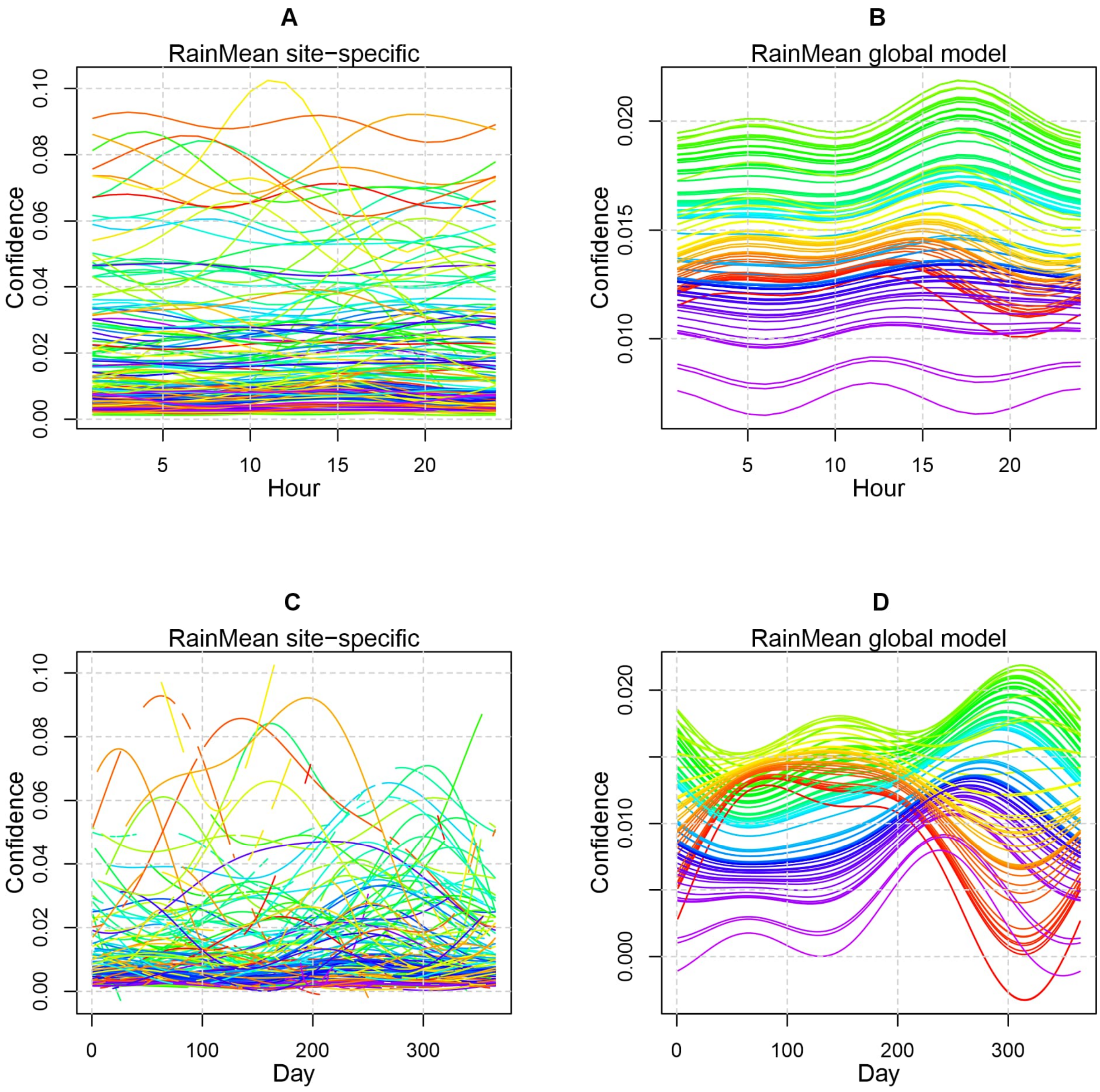

Supplementary Figure 20. Predictions of RainMean for the hour of the day (A, B) and the day of the year (C, D) for all sites in the study. Here, time is represented by the absolute hour. Site-specific models are displayed on the left (A, C), with global model predictions on the right (B, D). Each site is color-coded according to the map in Supplementary Figure 1. All sites included have data from at least 100 days except for 3 sites for which the accessible field season is very short (locations in the extreme north).

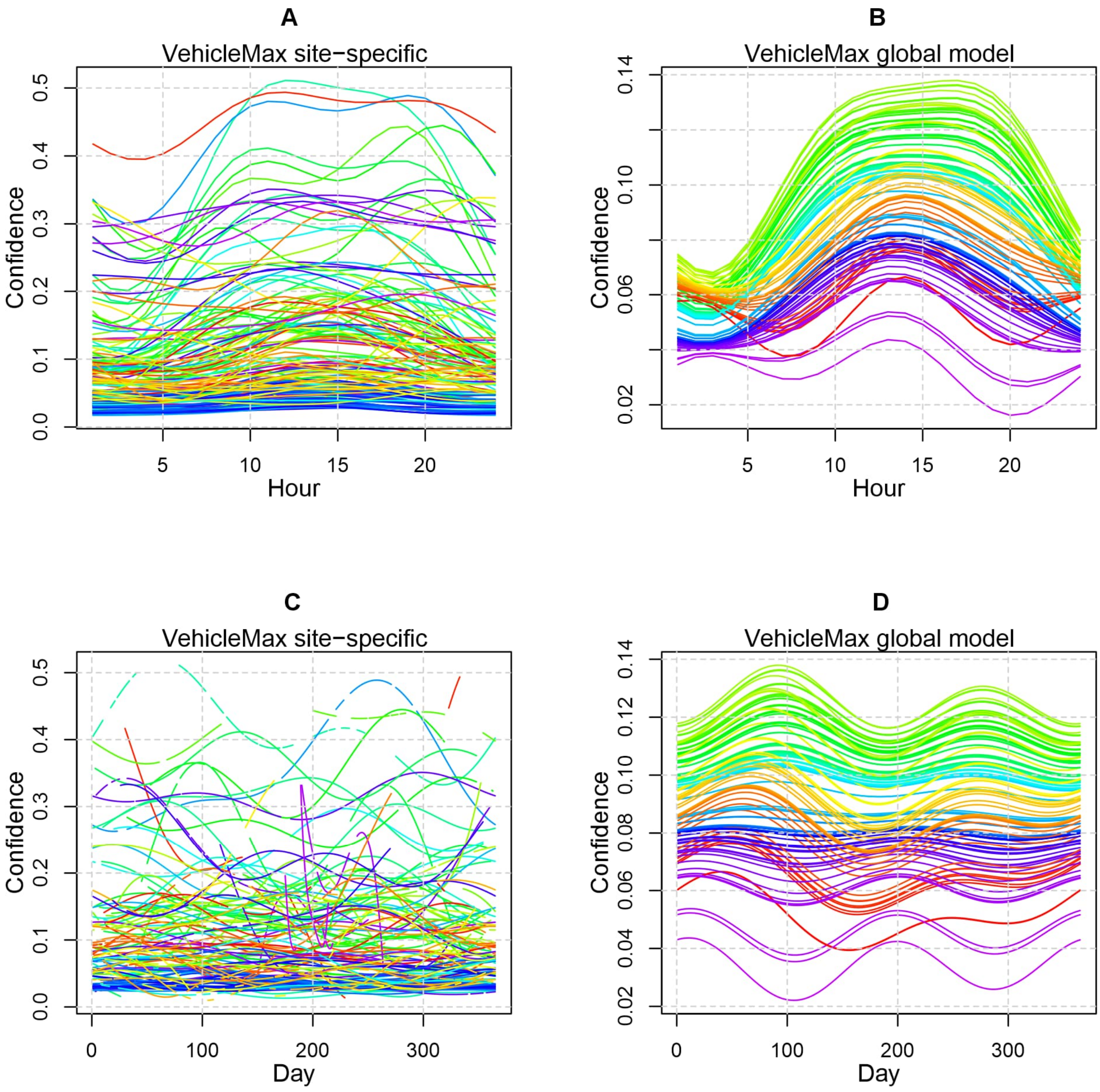

Supplementary Figure 21. Predictions of VehicleMax for the hour of the day (A, B) and the day of the year (C, D) for all sites in the study. Here, time is represented by the absolute hour. Site-specific models are displayed on the left (A, C), with global model predictions on the right (B, D). Each site is color-coded according to the map in Supplementary Figure 1. All sites included have data from at least 100 days except for 3 sites for which the accessible field season is very short (locations in the extreme north).

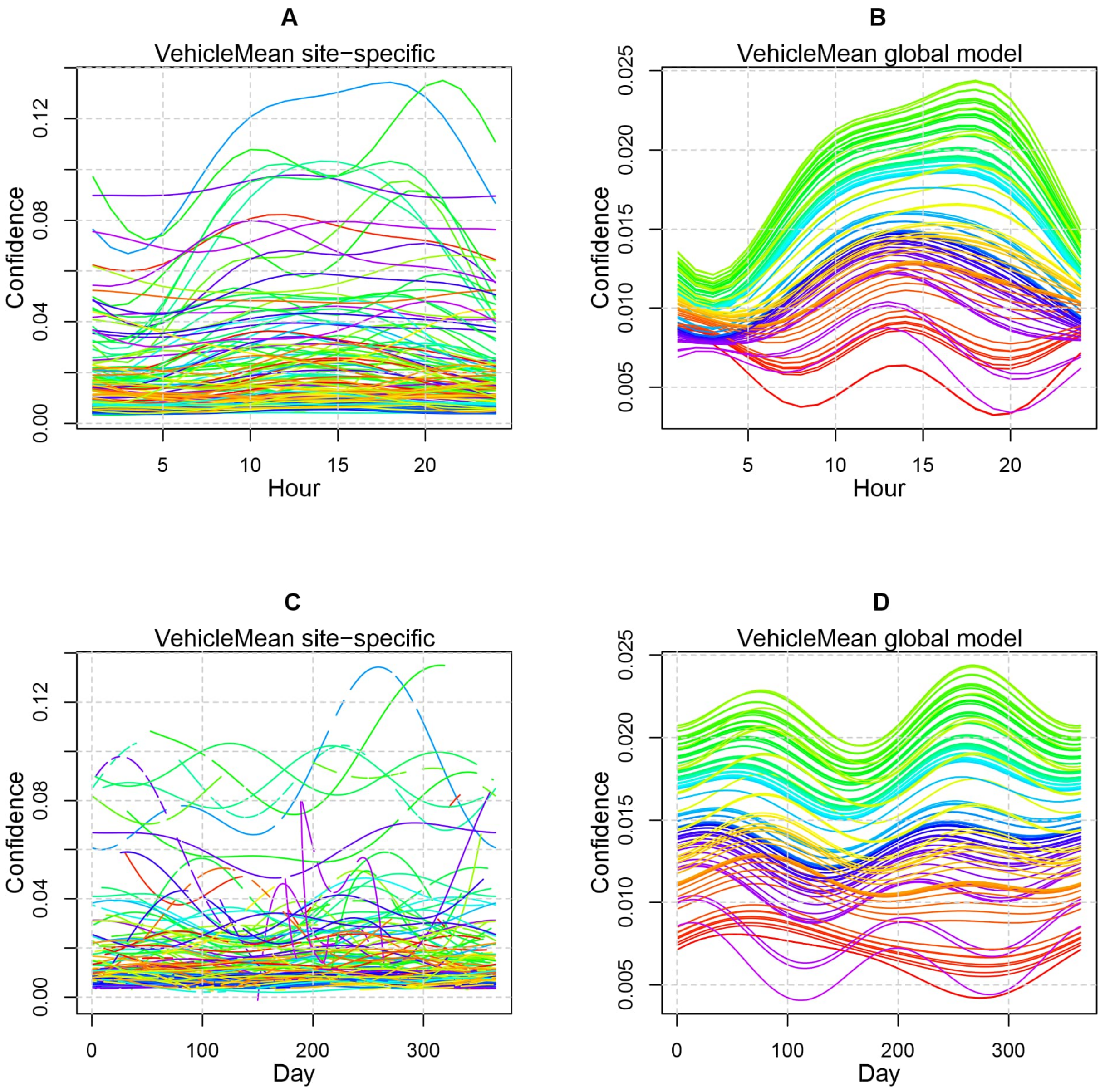

Supplementary Figure 22. Predictions of VehicleMean for the hour of the day (A, B) and the day of the year (C, D) for all sites in the study. Here, time is represented by the absolute hour. Site-specific models are displayed on the left (A, C), with global model predictions on the right (B, D). Each site is color-coded according to the map in Supplementary Figure 1. All sites included have data from at least 100 days except for 3 sites for which the accessible field season is very short (locations in the extreme north).

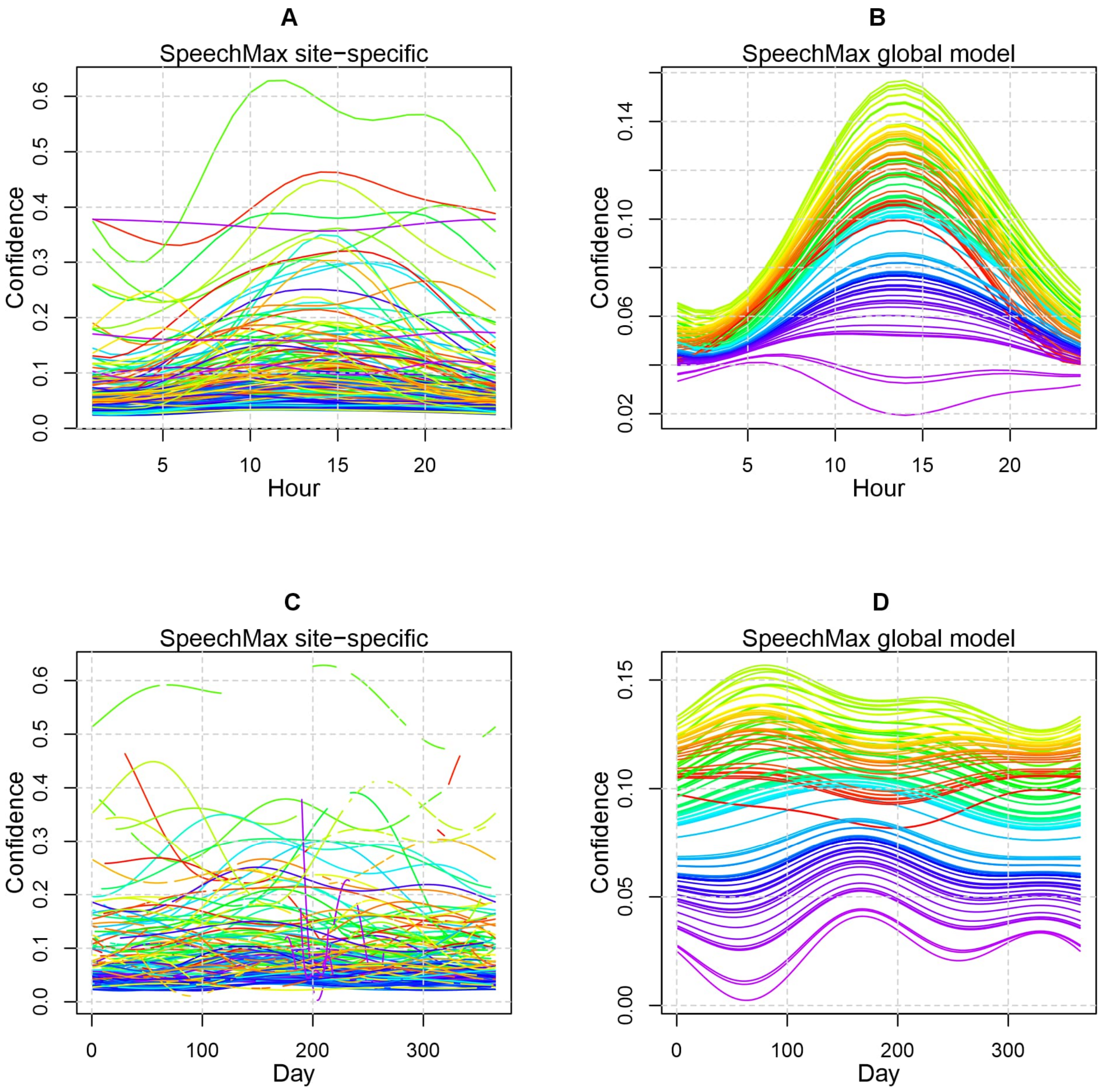

Supplementary Figure 23. Predictions of SpeechMax for the hour of the day (A, B) and the day of the year (C, D) for all sites in the study. Here, time is represented by the absolute hour. Site-specific models are displayed on the left (A, C), with global model predictions on the right (B, D). Each site is color-coded according to the map in Supplementary Figure 1. All sites included have data from at least 100 days except for 3 sites for which the accessible field season is very short (locations in the extreme north).

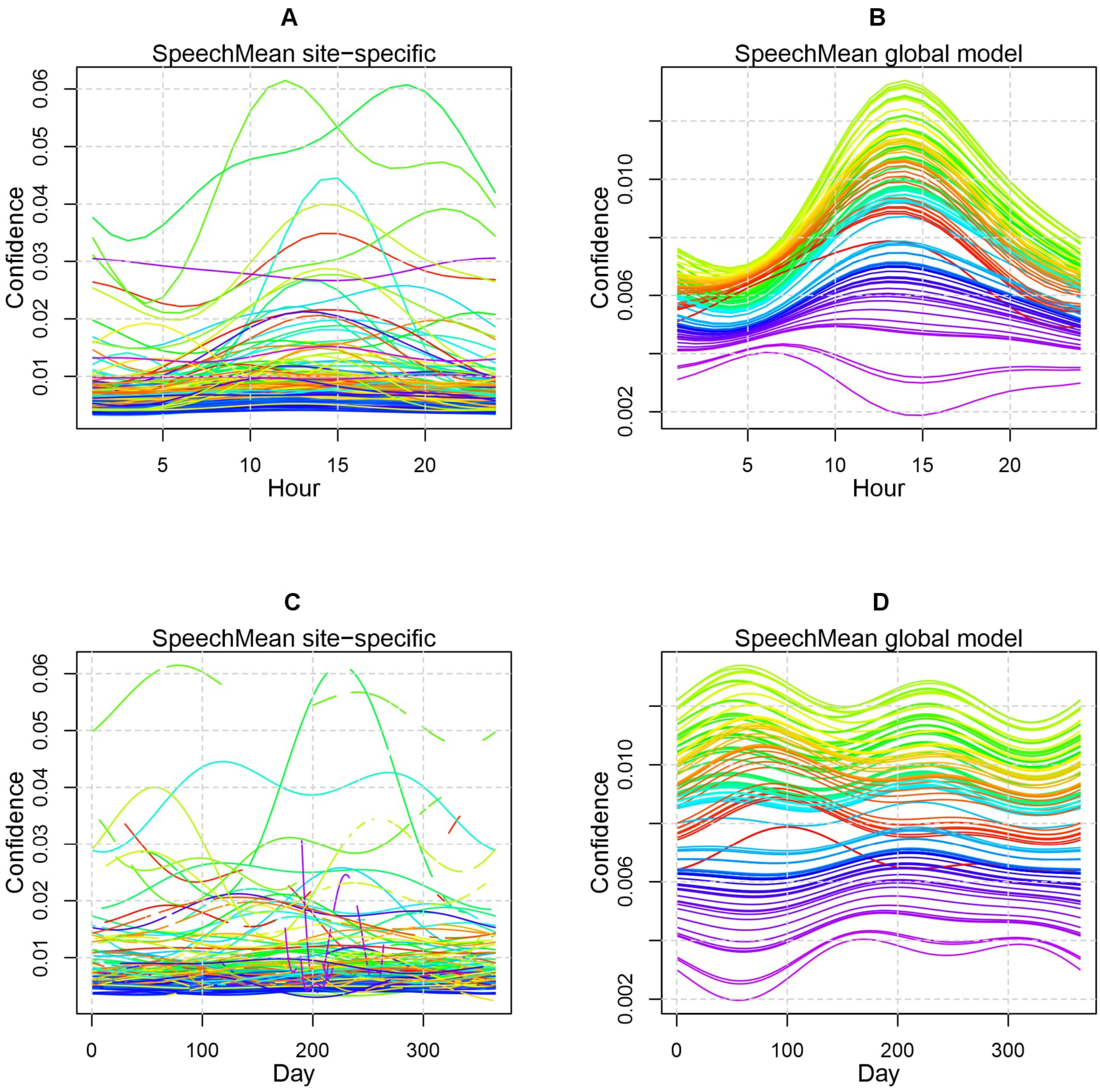

Supplementary Figure 24. Predictions of SpeechMean for the hour of the day (A, B) and the day of the year (C, D) for all sites in the study. Here, time is represented by the absolute hour. Site-specific models are displayed on the left (A, C), with global model predictions on the right (B, D). Each site is color-coded according to the map in Supplementary Figure 1. All sites included have data from at least 100 days except for 3 sites for which the accessible field season is very short (locations in the extreme north).

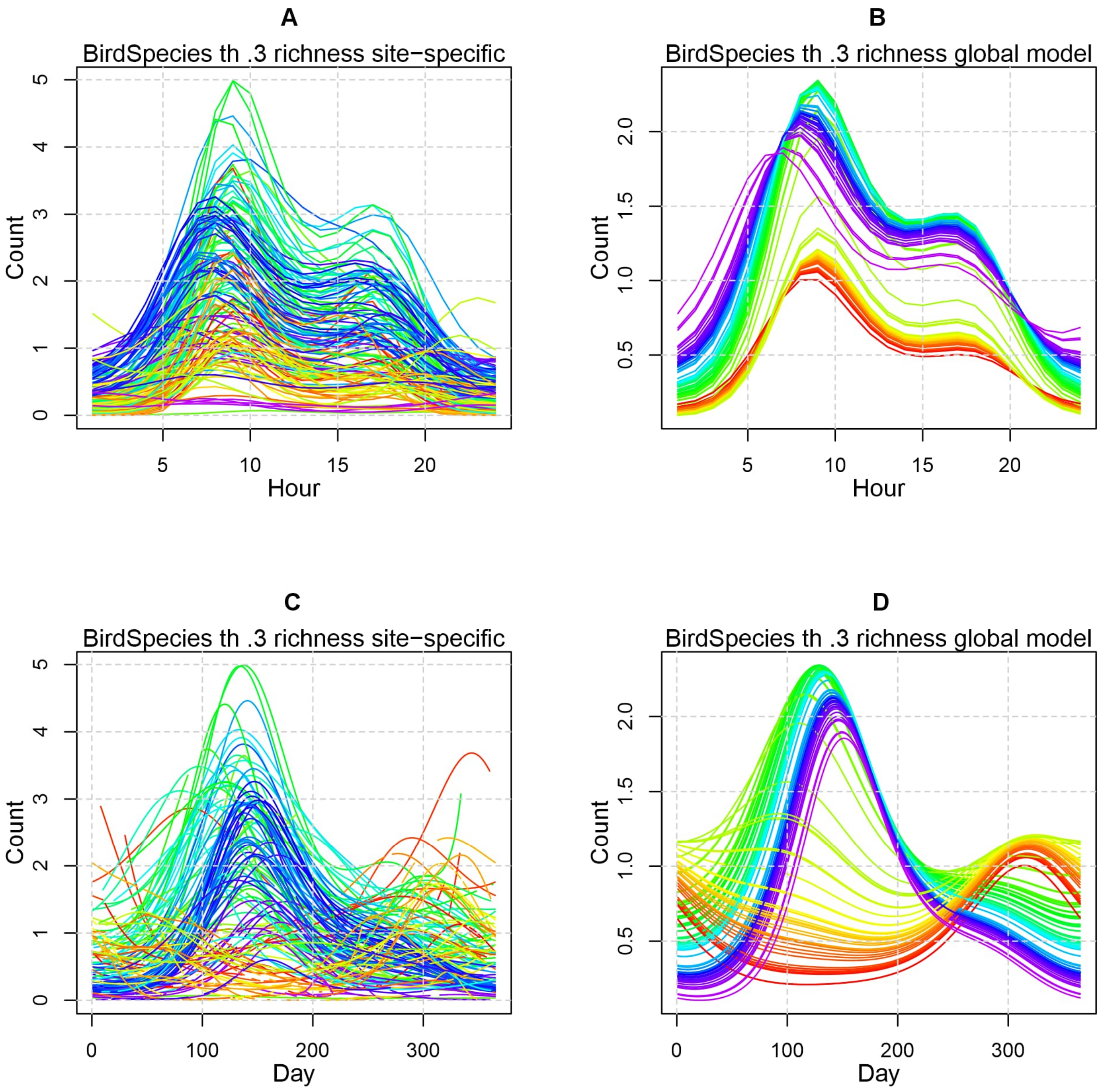

Supplementary Figure 25. Predictions of BirdSpecies with confidence threshold 0.3, measured as species richness, for the hour of the day (A, B) and the day of the year (C, D) for all sites in the study. Here, time is represented by the absolute hour. Site-specific models are displayed on the left (A, C), with global model predictions on the right (B, D). Each site is color-coded according to the map in Supplementary Figure 1. All sites included have data from at least 100 days except for 3 sites for which the accessible field season is very short (locations in the extreme north).

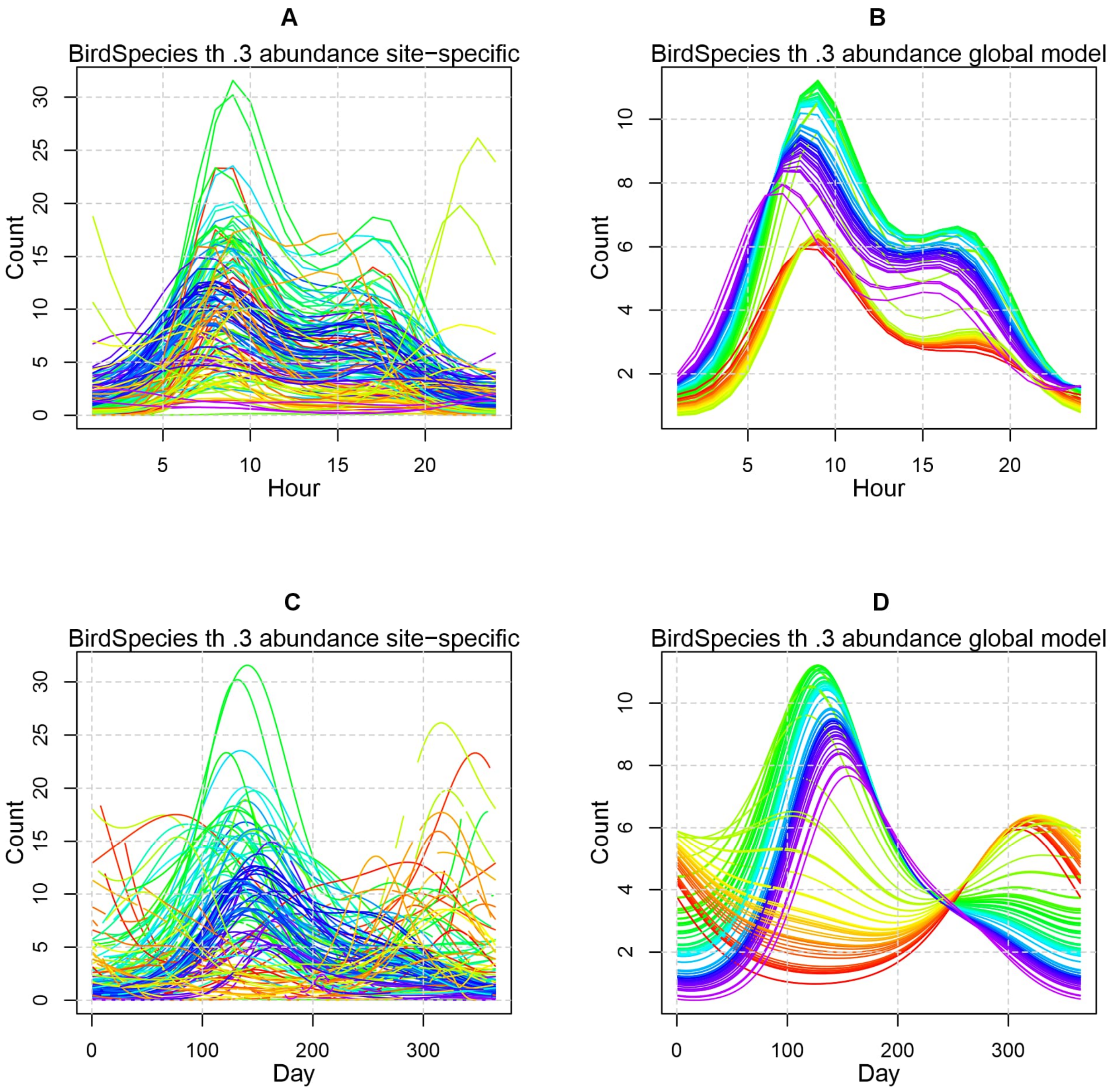

Supplementary Figure 26. Predictions of BirdSpecies with confidence threshold 0.3, measured as abundance, for the hour of the day (A, B) and the day of the year (C, D) for all sites in the study. Here, time is represented by the absolute hour. Site-specific models are displayed on the left (A, C), with global model predictions on the right (B, D). Each site is color-coded according to the map in Supplementary Figure 1. All sites included have data from at least 100 days except for 3 sites for which the accessible field season is very short (locations in the extreme north).

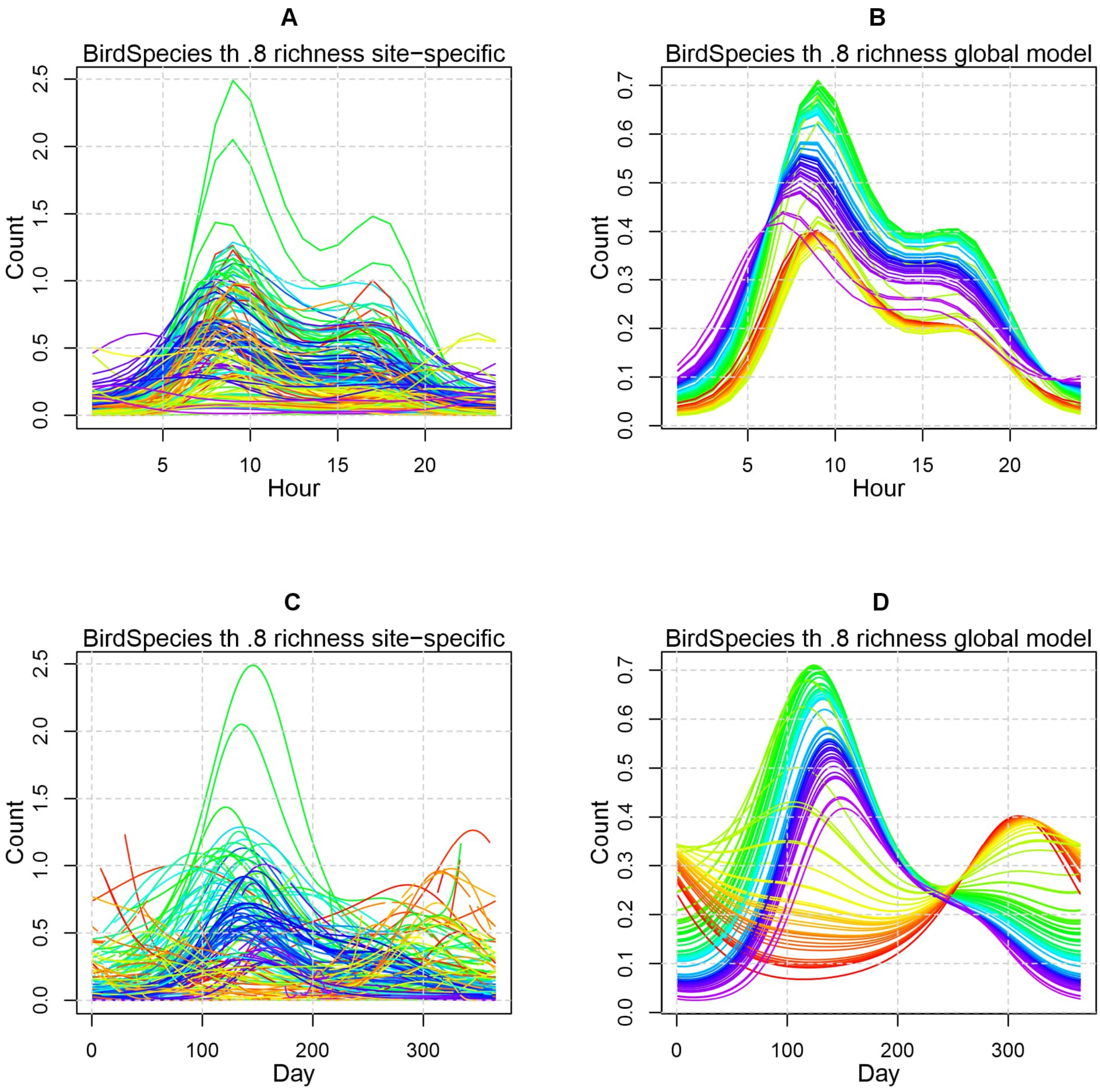

Supplementary Figure 27. Predictions of BirdSpecies with confidence threshold 0.8, measured as species richness, for the hour of the day (A, B) and the day of the year (C, D) for all sites in the study. Here, time is represented by the absolute hour. Site-specific models are displayed on the left (A, C), with global model predictions on the right (B, D). Each site is color-coded according to the map in Supplementary Figure 1. All sites included have data from at least 100 days except for 3 sites for which the accessible field season is very short (locations in the extreme north).

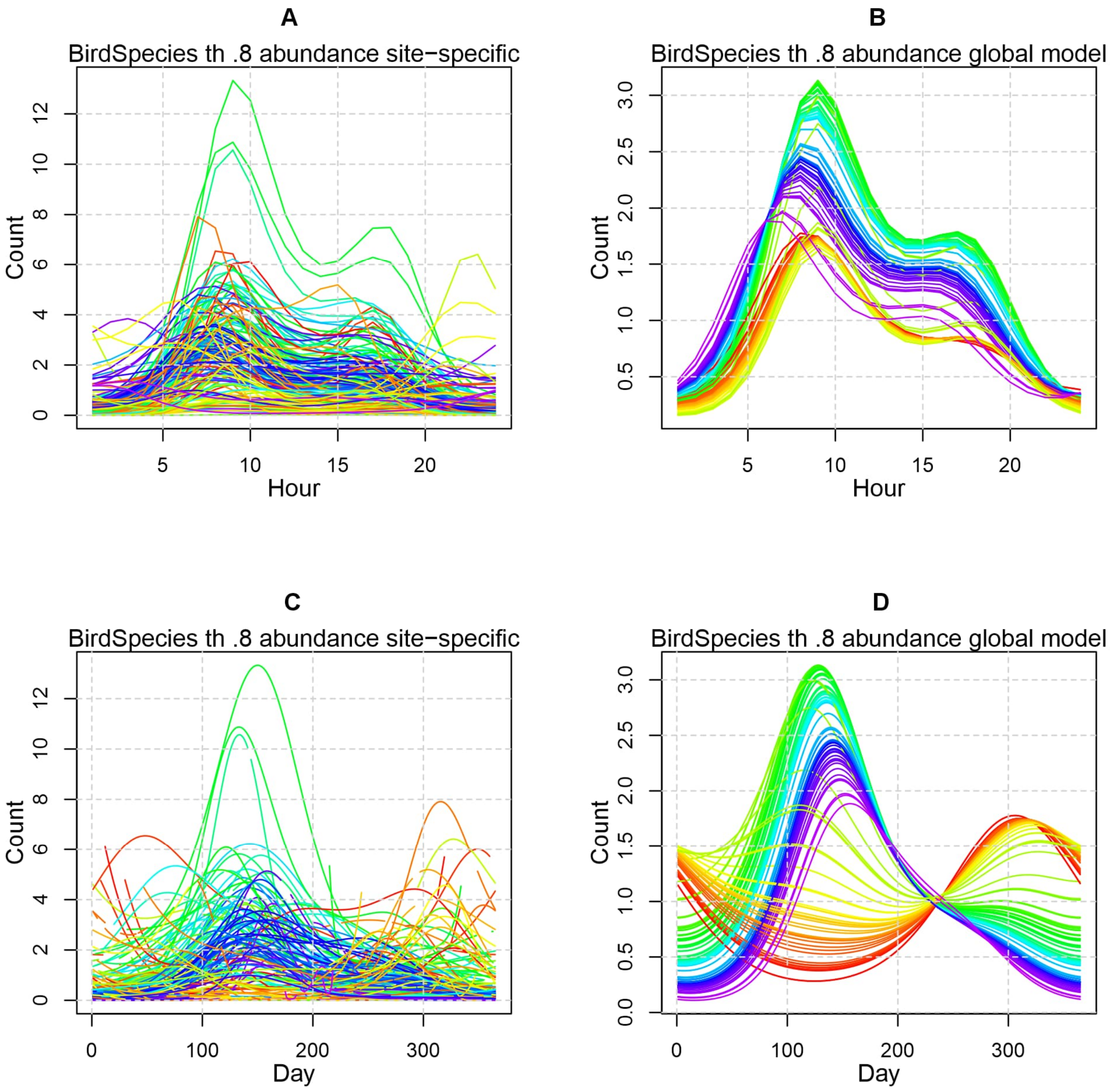

Supplementary Figure 28. Predictions of BirdSpecies with confidence threshold 0.8, measured as abundance, for the hour of the day (A, B) and the day of the year (C, D) for all sites in the study. Here, time is represented by the absolute hour. Site-specific models are displayed on the left (A, C), with global model predictions on the right (B, D). Each site is color-coded according to the map in Supplementary Figure 1. All sites included have data from at least 100 days except for 3 sites for which the accessible field season is very short (locations in the extreme north).

1.2 Graphs relative to the sun cycle.

In all graphs below (Supplementary Figure 29-S55), we show predictions from models where the local hour is represented by time relative to local sun set and sun rise.

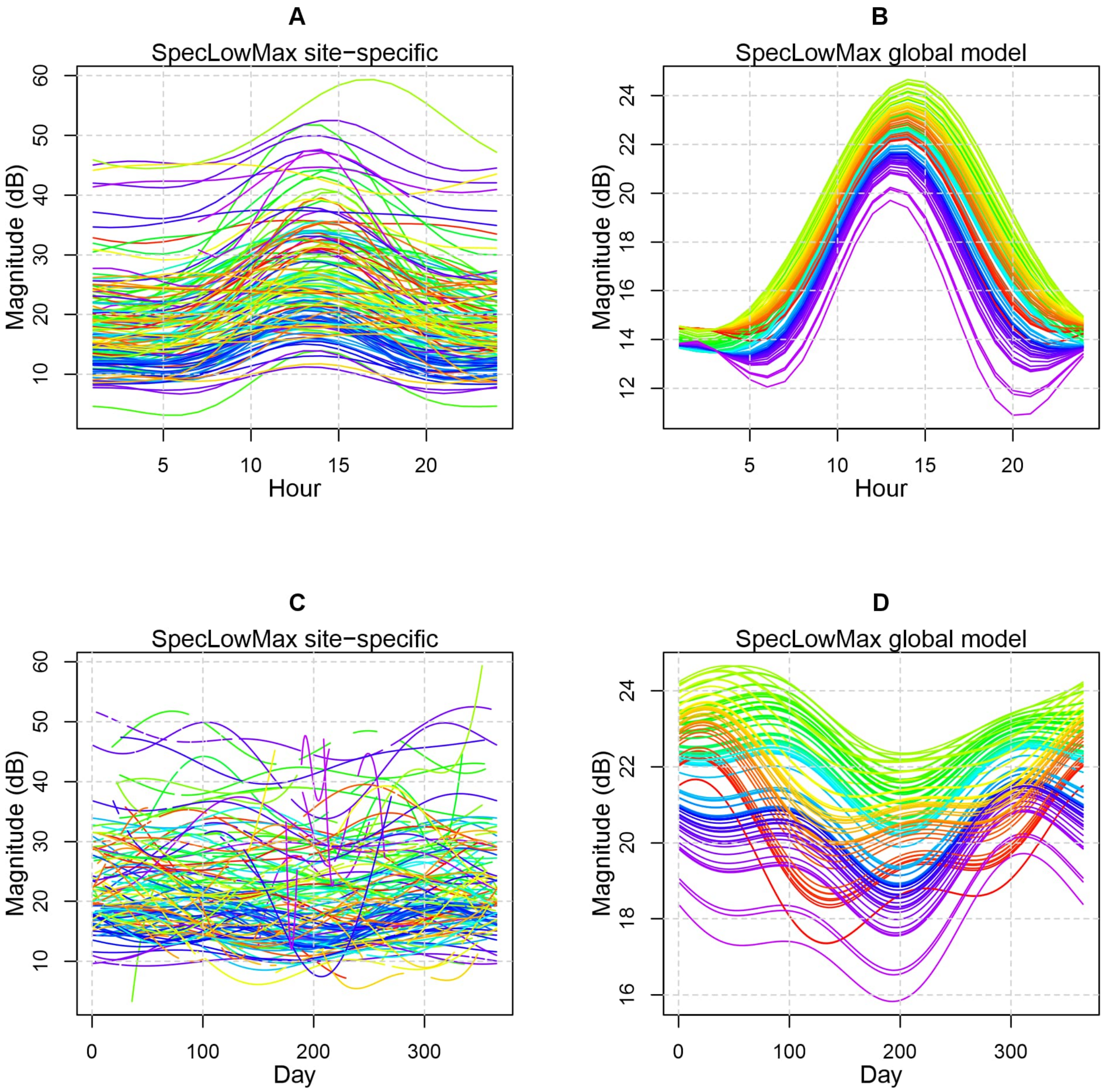

Supplementary Figure 29. Predictions of SpecLowMax for the hour of the day (A, B) and the day of the year (C, D) for all sites in the study. Here, time is represented by time relative to the sun cycle, mapped so that sunrise corresponds to 6AM and sunset to 6PM (see Methods). Site-specific models are displayed on the left (A, C), with global model predictions on the right (B, D). Each site is color-coded according to the map in Supplementary Figure 1. All sites included have data from at least 100 days except for 3 sites for which the accessible field season is very short (locations in the extreme north).

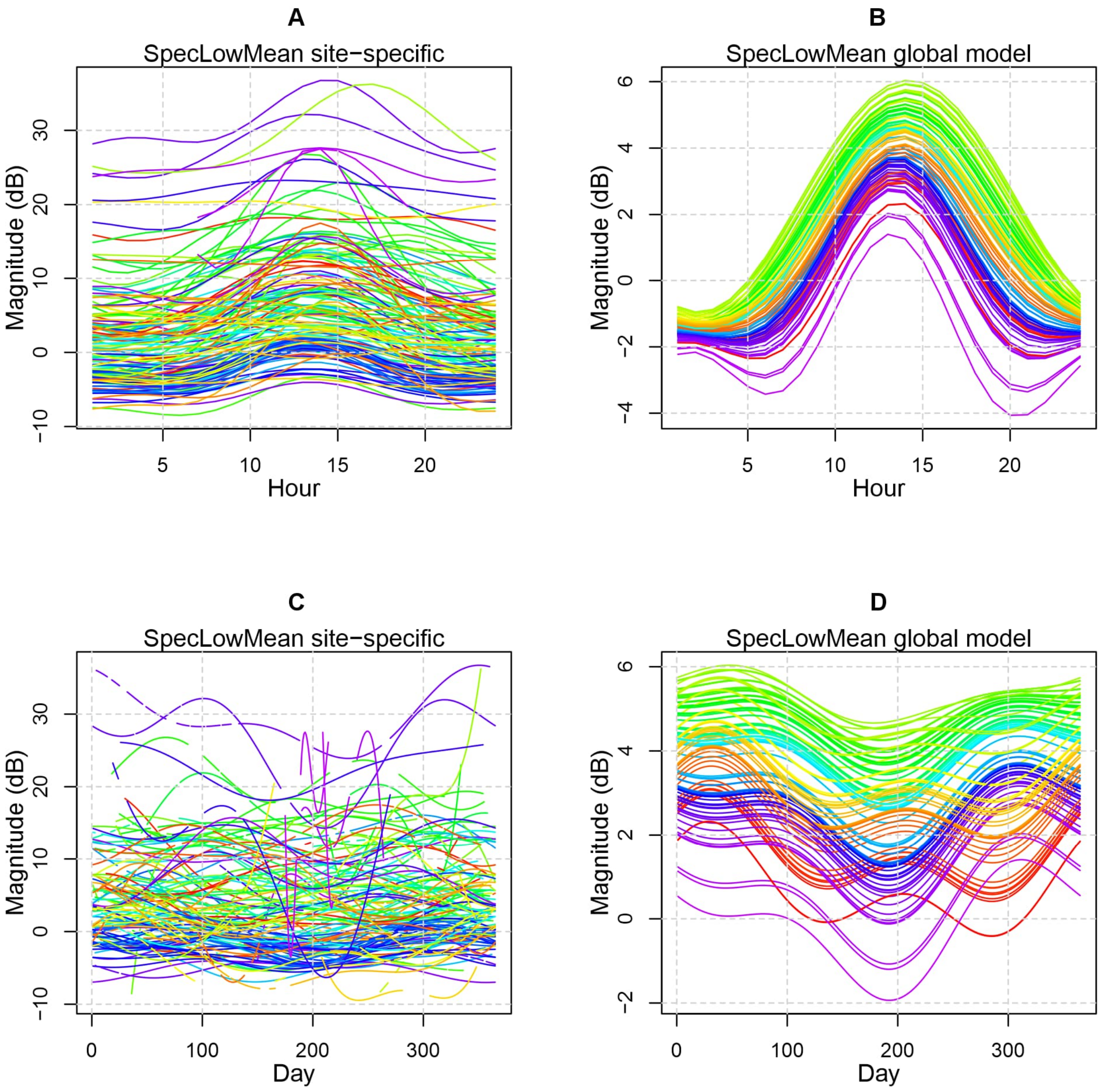

Supplementary Figure 30. Predictions of SpecLowMean for the hour of the day (A, B) and the day of the year (C, D) for all sites in the study. Here, time is represented by time relative to the sun cycle, mapped so that sunrise corresponds to 6AM and sunset to 6PM (see Methods). Site-specific models are displayed on the left (A, C), with global model predictions on the right (B, D). Each site is color-coded according to the map in Supplementary Figure 1. All sites included have data from at least 100 days except for 3 sites for which the accessible field season is very short (locations in the extreme north).

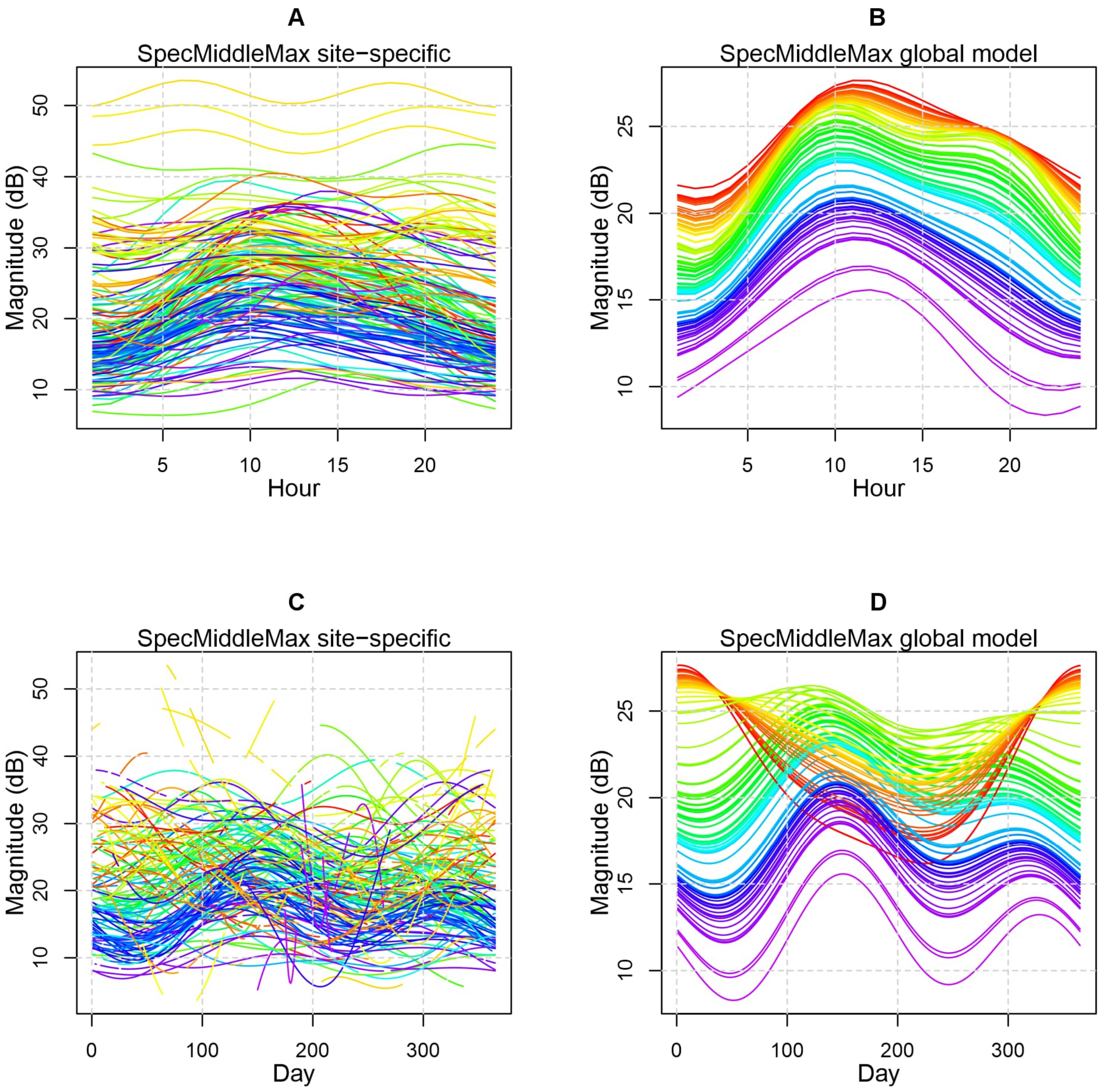

Supplementary Figure 31. Predictions of SpecMiddleMax for the hour of the day (A, B) and the day of the year (C, D) for all sites in the study. Here, time is represented by time relative to the sun cycle, mapped so that sunrise corresponds to 6AM and sunset to 6PM (see Methods). Site-specific models are displayed on the left (A, C), with global model predictions on the right (B, D). Each site is color-coded according to the map in Supplementary Figure 1. All sites included have data from at least 100 days except for 3 sites for which the accessible field season is very short (locations in the extreme north).

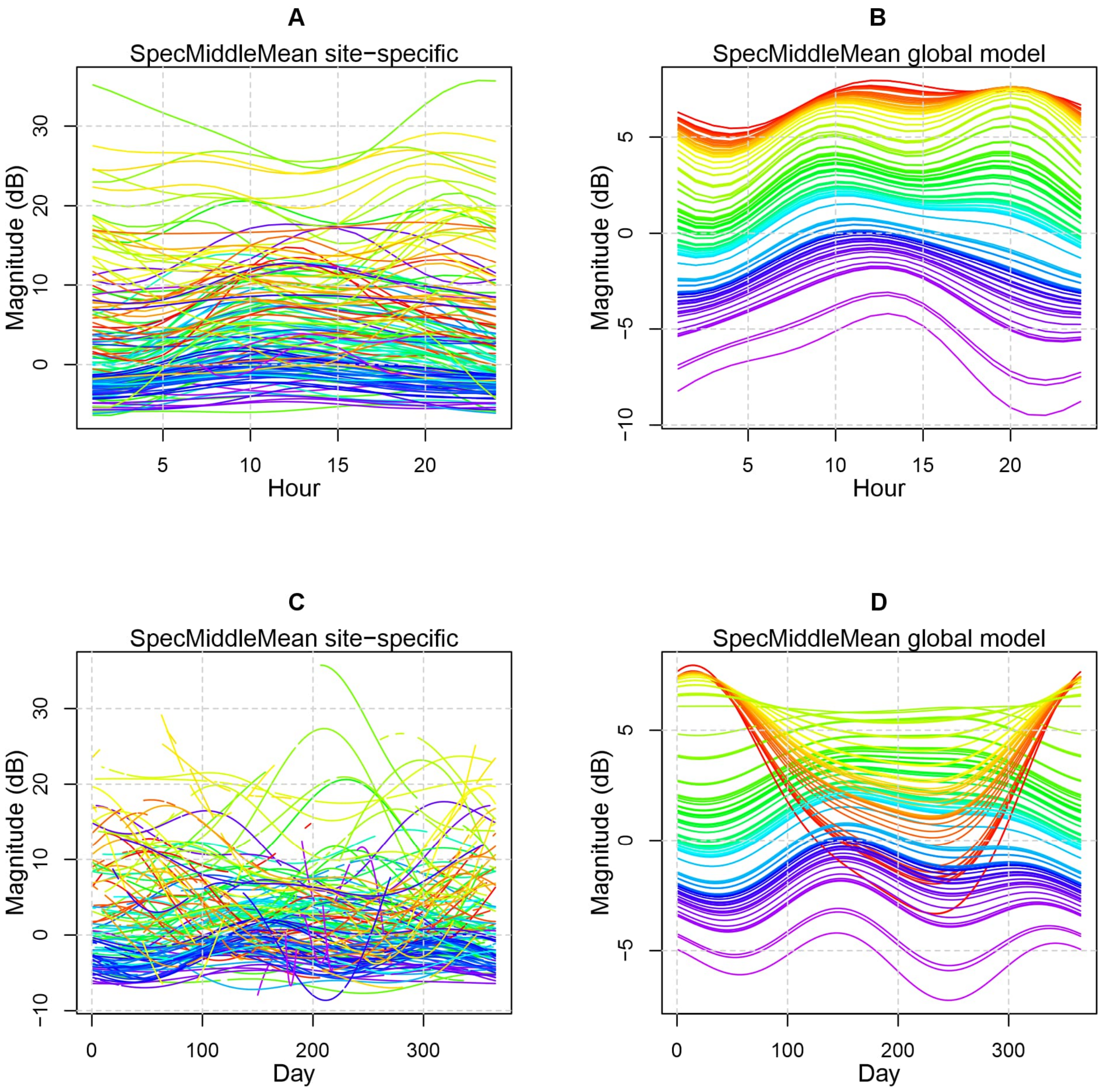

Supplementary Figure 32. Predictions of SpecMiddleMean for the hour of the day (A, B) and the day of the year (C, D) for all sites in the study. Here, time is represented by time relative to the sun cycle, mapped so that sunrise corresponds to 6AM and sunset to 6PM (see Methods). Site-specific models are displayed on the left (A, C), with global model predictions on the right (B, D). Each site is color-coded according to the map in Supplementary Figure 1. All sites included have data from at least 100 days except for 3 sites for which the accessible field season is very short (locations in the extreme north).

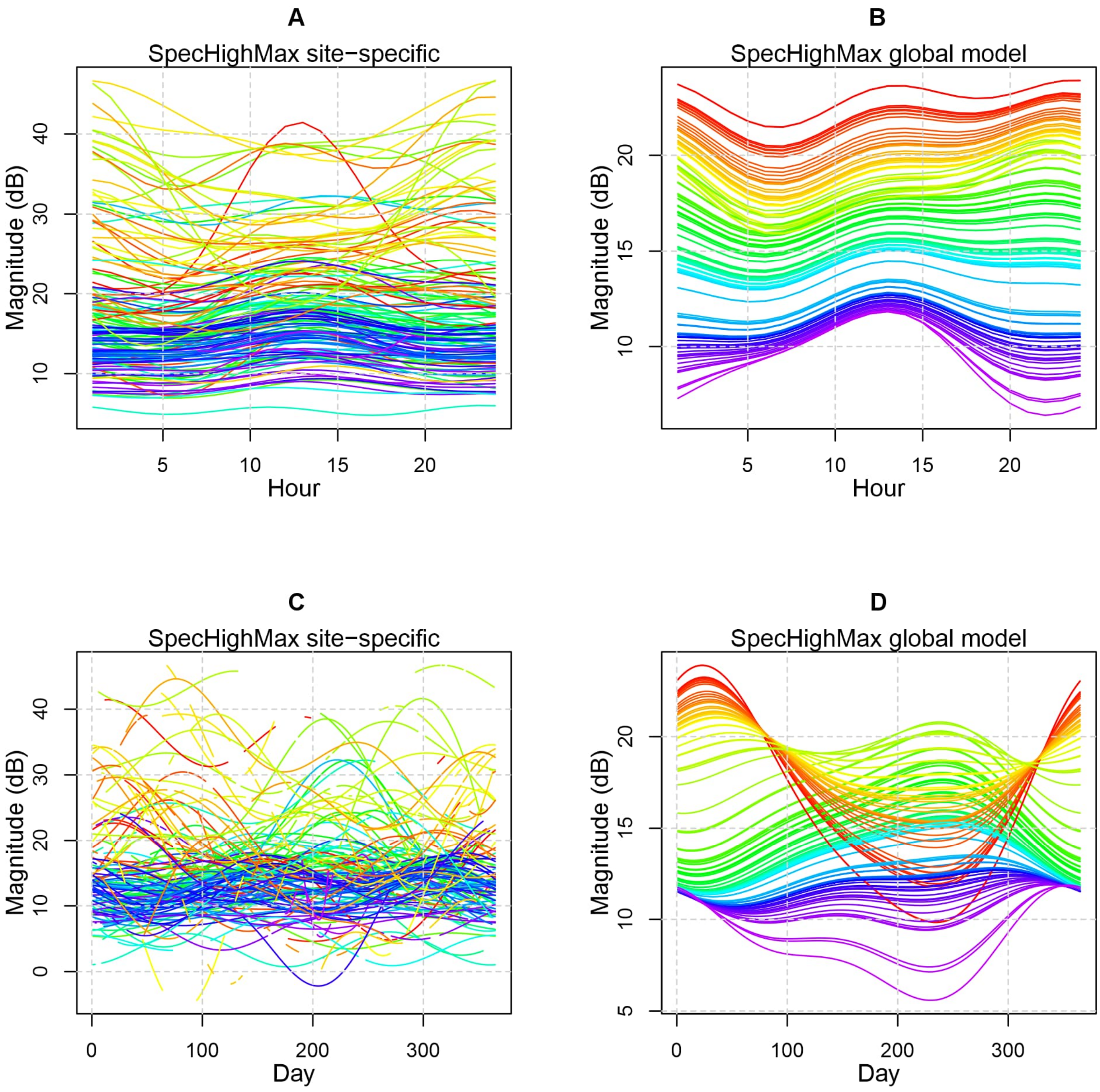

Supplementary Figure 33. Predictions of SpecHighMax for the hour of the day (A, B) and the day of the year (C, D) for all sites in the study. Here, time is represented by time relative to the sun cycle, mapped so that sunrise corresponds to 6AM and sunset to 6PM (see Methods). Site-specific models are displayed on the left (A, C), with global model predictions on the right (B, D). Each site is color-coded according to the map in Supplementary Figure 1. All sites included have data from at least 100 days except for 3 sites for which the accessible field season is very short (locations in the extreme north).

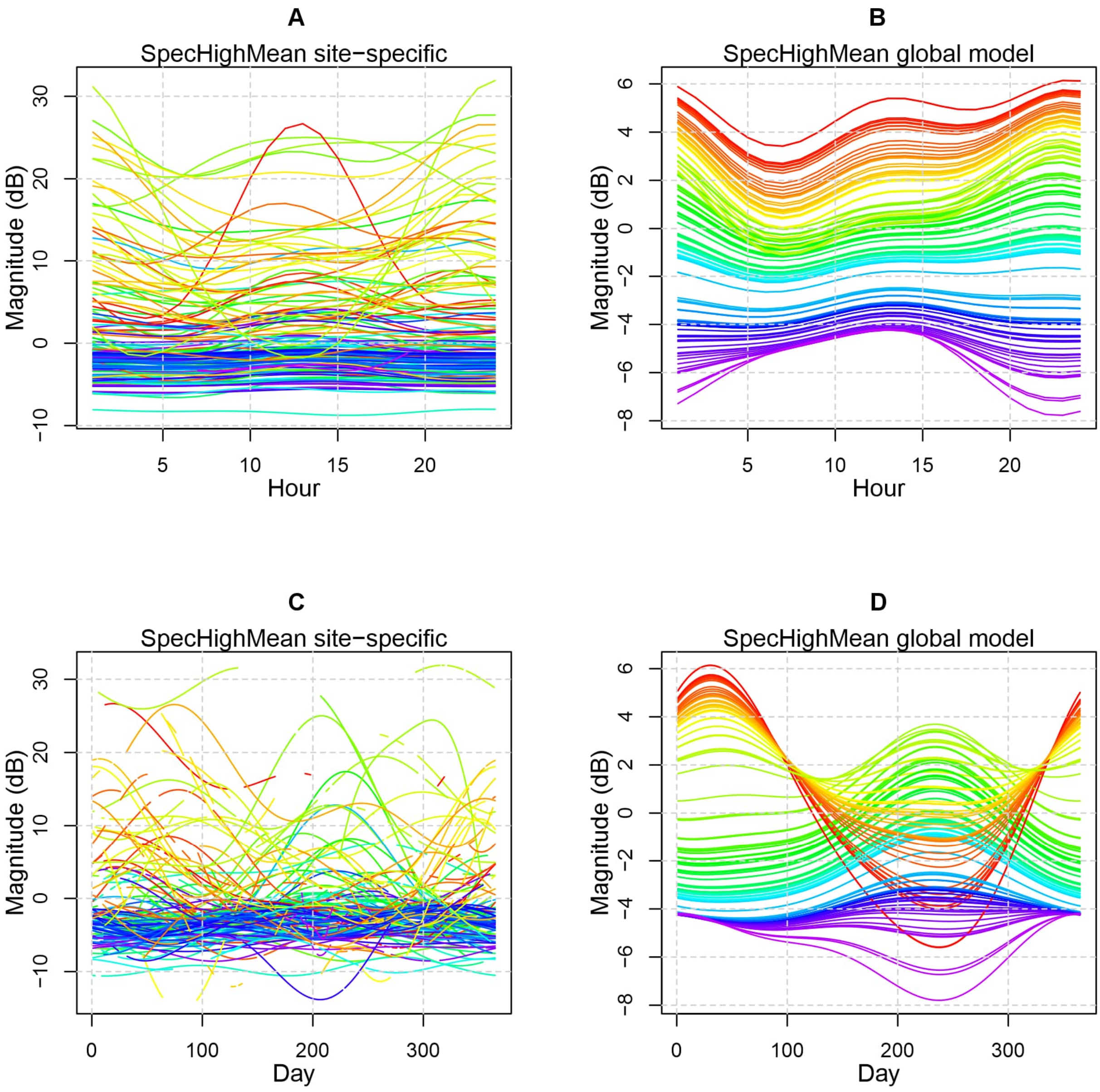

Supplementary Figure 34. Predictions of SpecHighMean for the hour of the day (A, B) and the day of the year (C, D) for all sites in the study. Here, time is represented by time relative to the sun cycle, mapped so that sunrise corresponds to 6AM and sunset to 6PM (see Methods). Site-specific models are displayed on the left (A, C), with global model predictions on the right (B, D). Each site is color-coded according to the map in Supplementary Figure 1. All sites included have data from at least 100 days except for 3 sites for which the accessible field season is very short (locations in the extreme north).

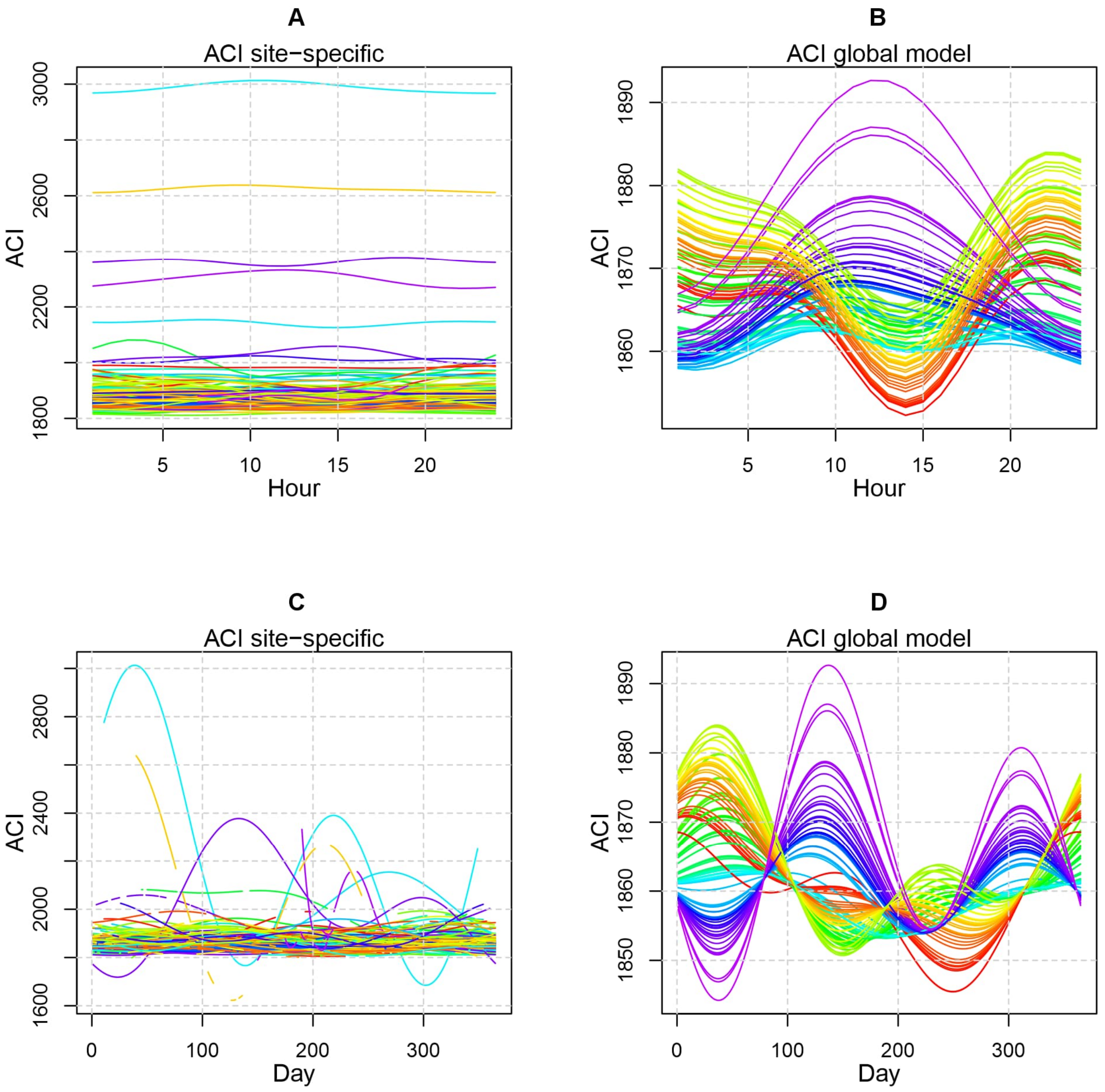

Supplementary Figure 35. Predictions of ACI for the hour of the day (A, B) and the day of the year (C, D) for all sites in the study. Here, time is represented by time relative to the sun cycle, mapped so that sunrise corresponds to 6AM and sunset to 6PM (see Methods). Site-specific models are displayed on the left (A, C), with global model predictions on the right (B, D). Each site is color-coded according to the map in Supplementary Figure 1. All sites included have data from at least 100 days except for 3 sites for which the accessible field season is very short (locations in the extreme north).

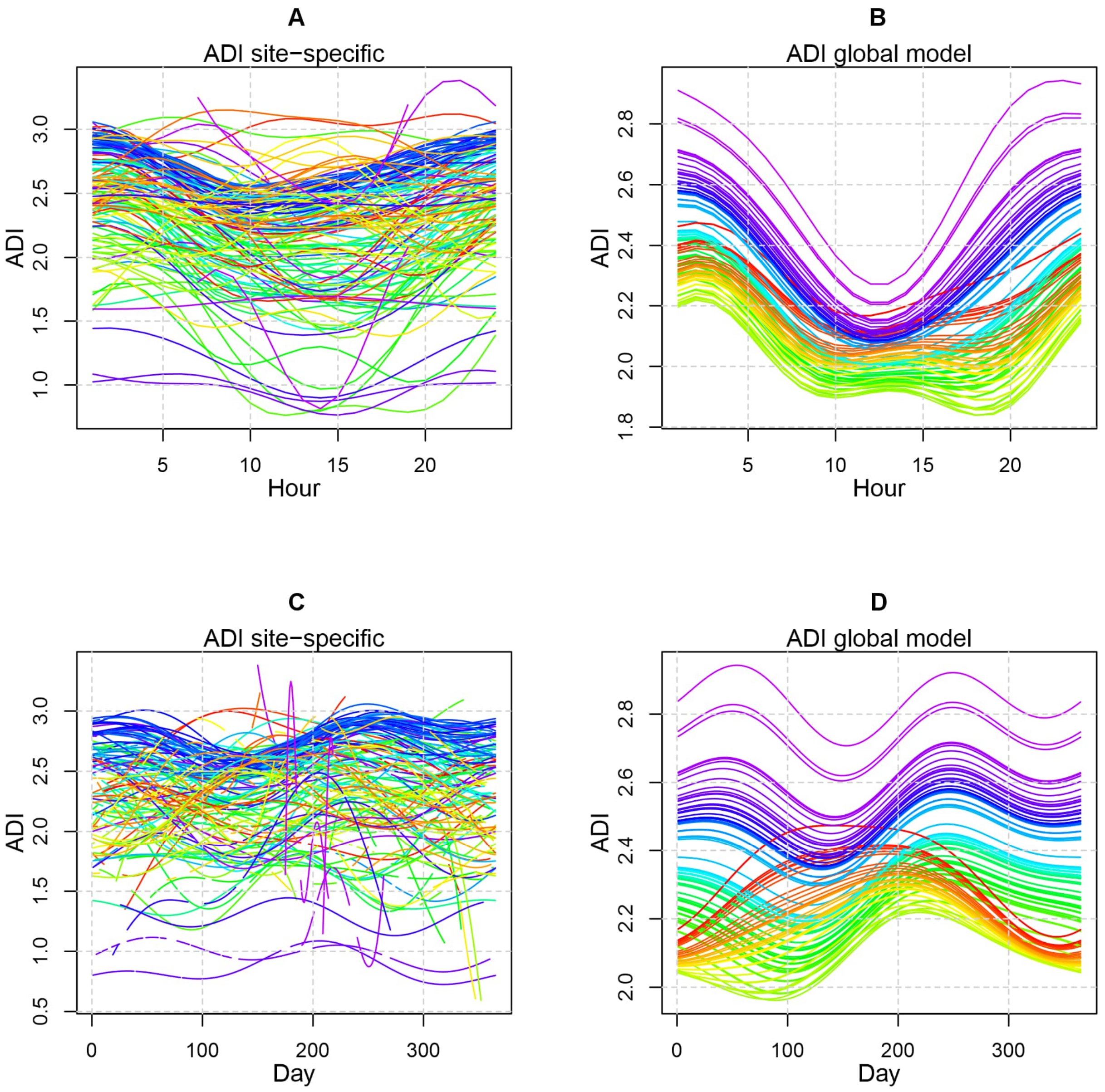

Supplementary Figure 36. Predictions of ADI for the hour of the day (A, B) and the day of the year (C, D) for all sites in the study. Here, time is represented by time relative to the sun cycle, mapped so that sunrise corresponds to 6AM and sunset to 6PM (see Methods). Site-specific models are displayed on the left (A, C), with global model predictions on the right (B, D). Each site is color-coded according to the map in Supplementary Figure 1. All sites included have data from at least 100 days except for 3 sites for which the accessible field season is very short (locations in the extreme north).

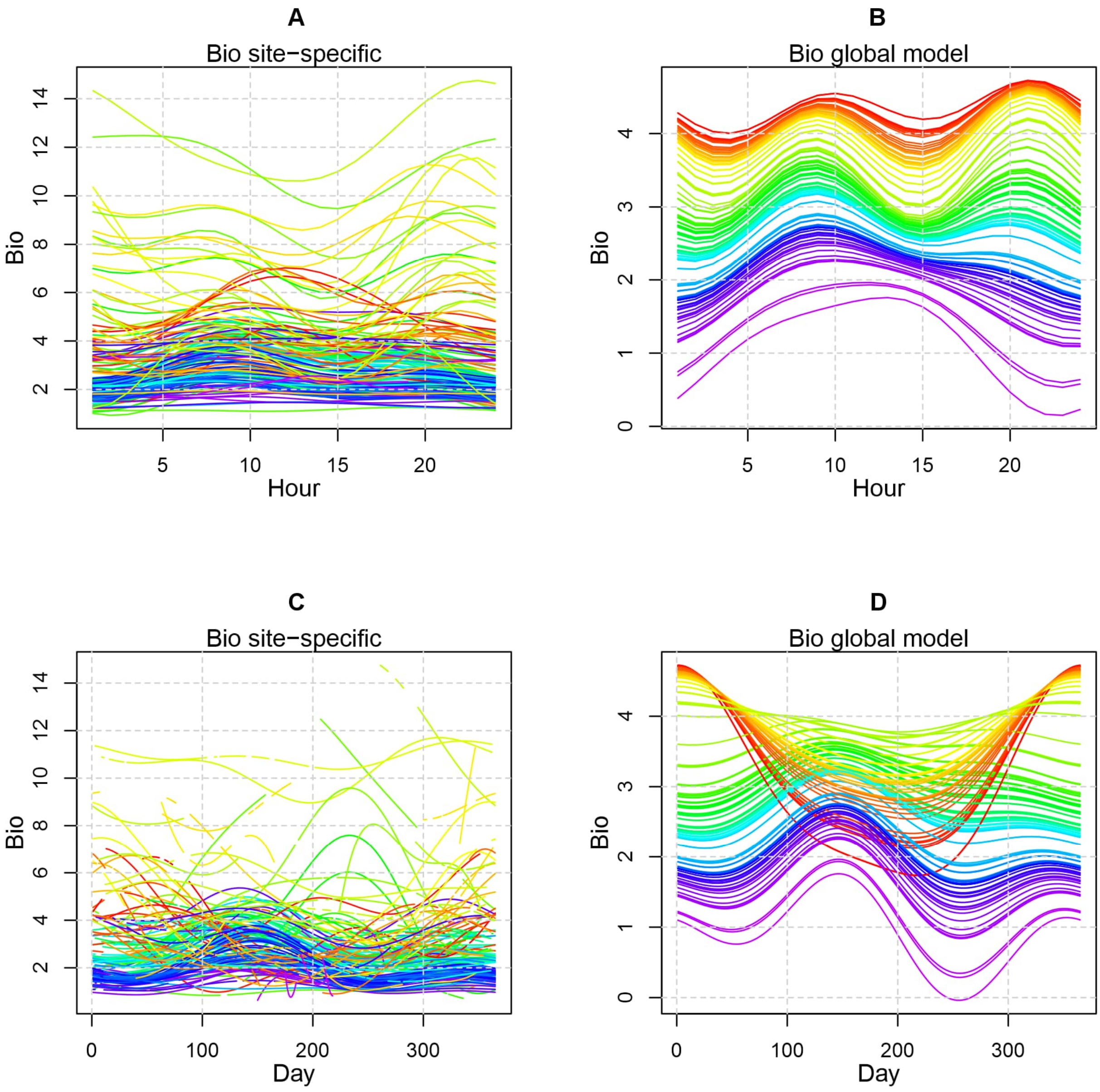

Supplementary Figure 37. Predictions of Bio for the hour of the day (A, B) and the day of the year (C, D) for all sites in the study. Here, time is represented by time relative to the sun cycle, mapped so that sunrise corresponds to 6AM and sunset to 6PM (see Methods). Site-specific models are displayed on the left (A, C), with global model predictions on the right (B, D). Each site is color-coded according to the map in Supplementary Figure 1. All sites included have data from at least 100 days except for 3 sites for which the accessible field season is very short (locations in the extreme north).

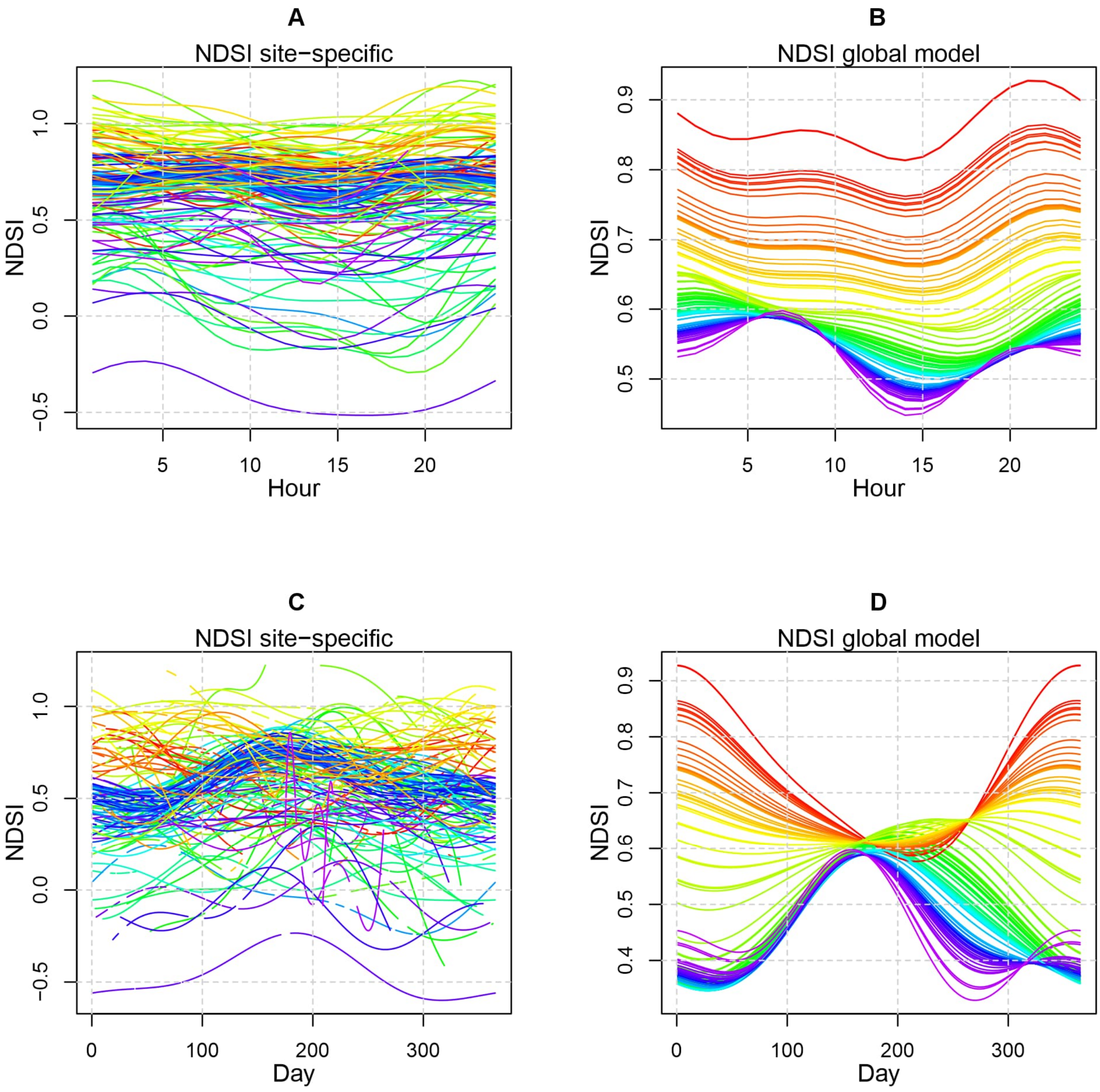

Supplementary Figure 38. Predictions of NDSI for the hour of the day (A, B) and the day of the year (C, D) for all sites in the study. Here, time is represented by time relative to the sun cycle, mapped so that sunrise corresponds to 6AM and sunset to 6PM (see Methods). Site-specific models are displayed on the left (A, C), with global model predictions on the right (B, D). Each site is color-coded according to the map in Supplementary Figure 1. All sites included have data from at least 100 days except for 3 sites for which the accessible field season is very short (locations in the extreme north).

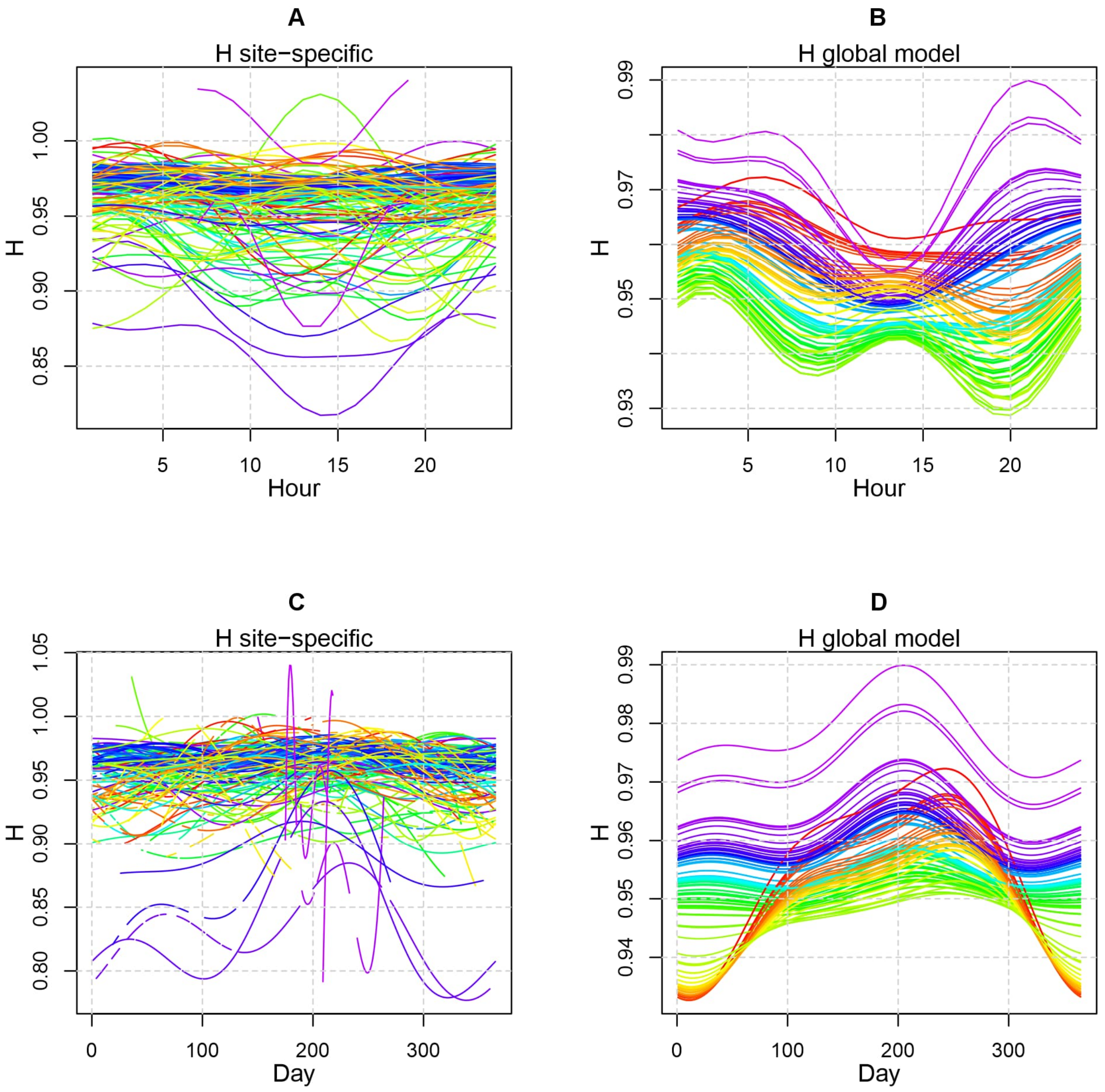

Supplementary Figure 39. Predictions of H for the hour of the day (A, B) and the day of the year (C, D) for all sites in the study. Here, time is represented by time relative to the sun cycle, mapped so that sunrise corresponds to 6AM and sunset to 6PM (see Methods). Site-specific models are displayed on the left (A, C), with global model predictions on the right (B, D). Each site is color-coded according to the map in Supplementary Figure 1. All sites included have data from at least 100 days except for 3 sites for which the accessible field season is very short (locations in the extreme north).

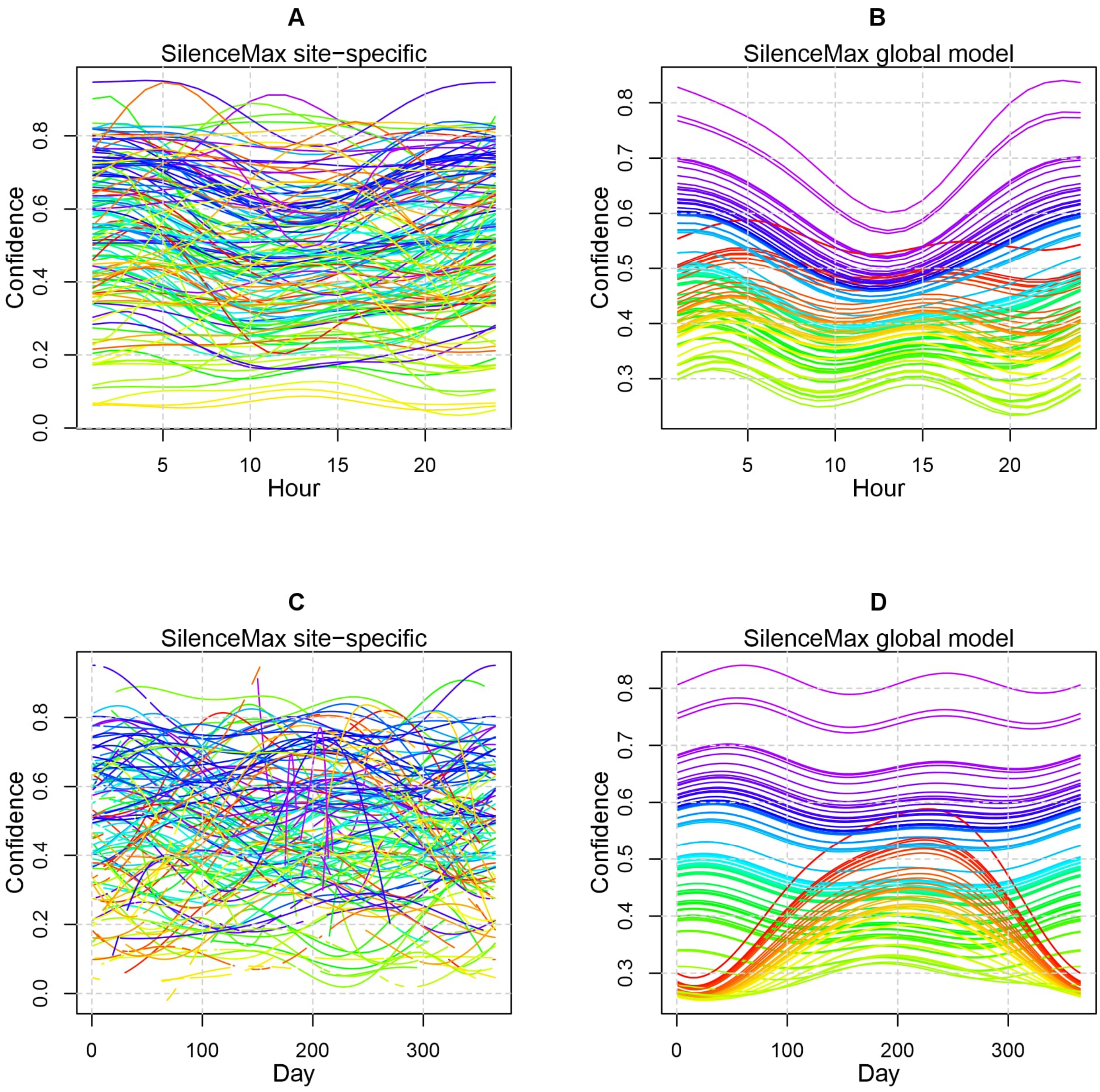

Supplementary Figure 40. Predictions of SilenceMax for the hour of the day (A, B) and the day of the year (C, D) for all sites in the study. Here, time is represented by time relative to the sun cycle, mapped so that sunrise corresponds to 6AM and sunset to 6PM (see Methods). Site-specific models are displayed on the left (A, C), with global model predictions on the right (B, D). Each site is color-coded according to the map in Supplementary Figure 1. All sites included have data from at least 100 days except for 3 sites for which the accessible field season is very short (locations in the extreme north).

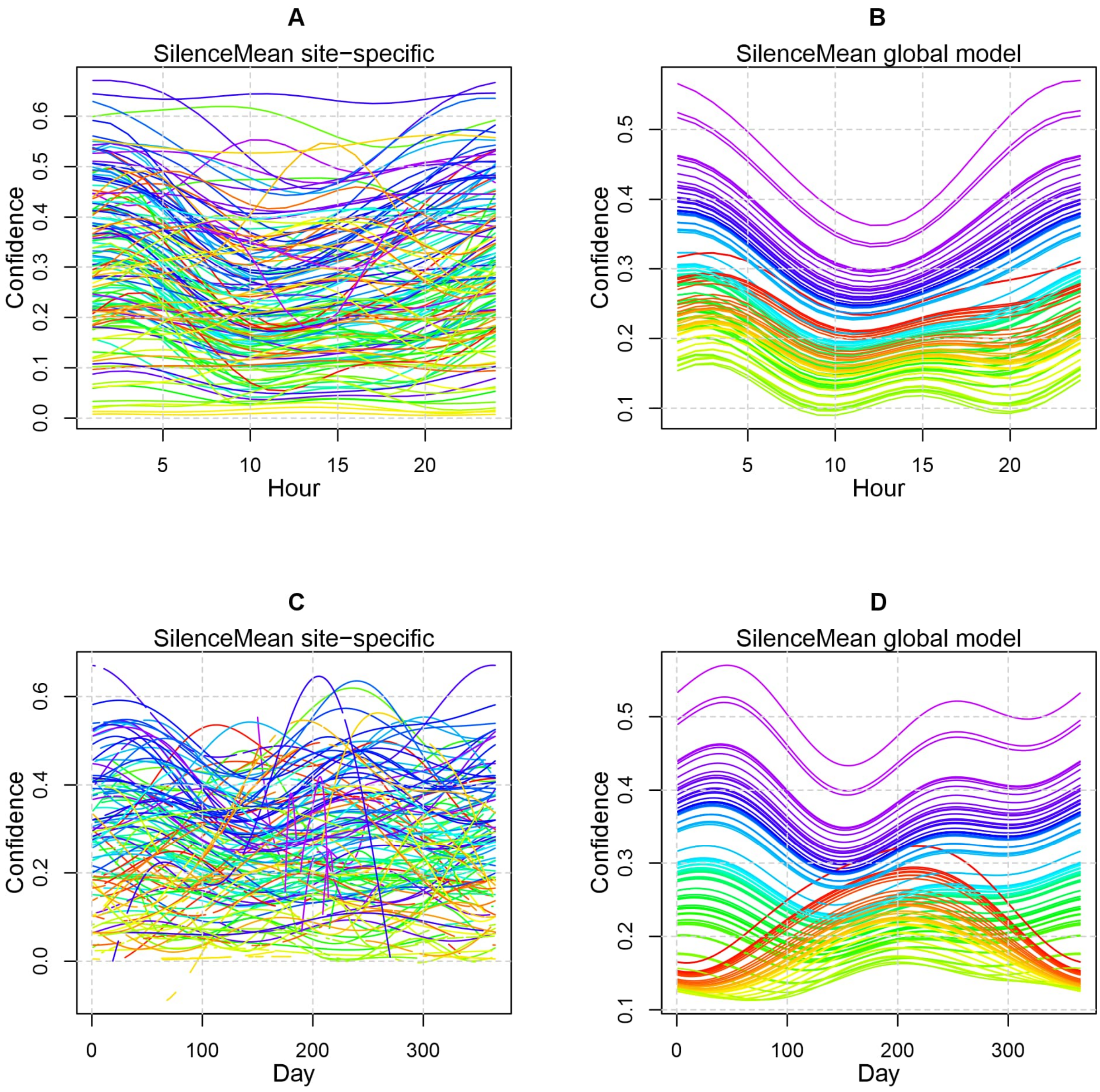

Supplementary Figure 41. Predictions of SilenceMean for the hour of the day (A, B) and the day of the year (C, D) for all sites in the study. Here, time is represented by time relative to the sun cycle, mapped so that sunrise corresponds to 6AM and sunset to 6PM (see Methods). Site-specific models are displayed on the left (A, C), with global model predictions on the right (B, D). Each site is color-coded according to the map in Supplementary Figure 1. All sites included have data from at least 100 days except for 3 sites for which the accessible field season is very short (locations in the extreme north).

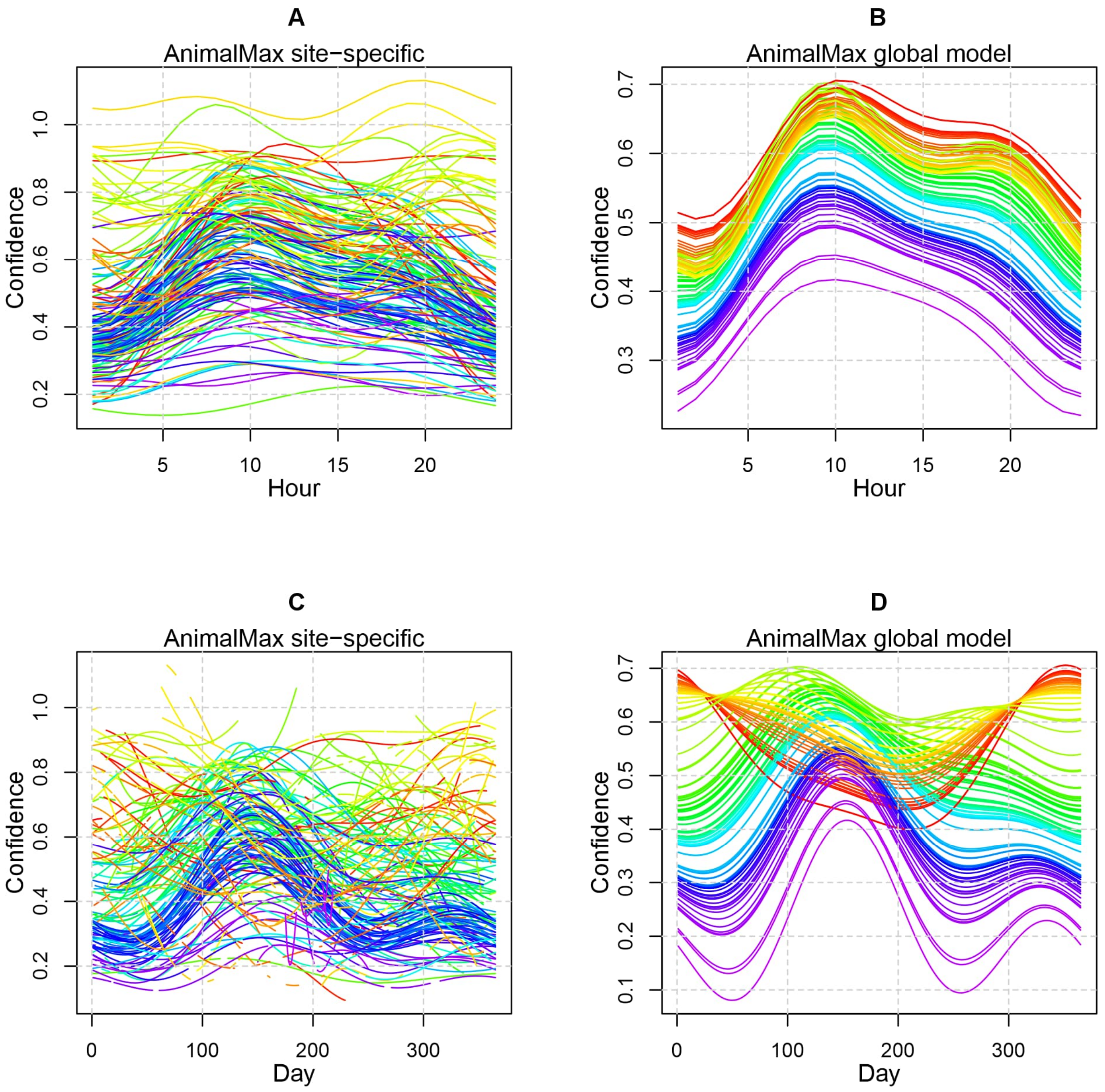

Supplementary Figure 42. Predictions of SilenceMax for the hour of the day (A, B) and the day of the year (C, D) for all sites in the study. Here, time is represented by time relative to the sun cycle, mapped so that sunrise corresponds to 6AM and sunset to 6PM (see Methods). Site-specific models are displayed on the left (A, C), with global model predictions on the right (B, D). Each site is color-coded according to the map in Supplementary Figure 1. All sites included have data from at least 100 days except for 3 sites for which the accessible field season is very short (locations in the extreme north).

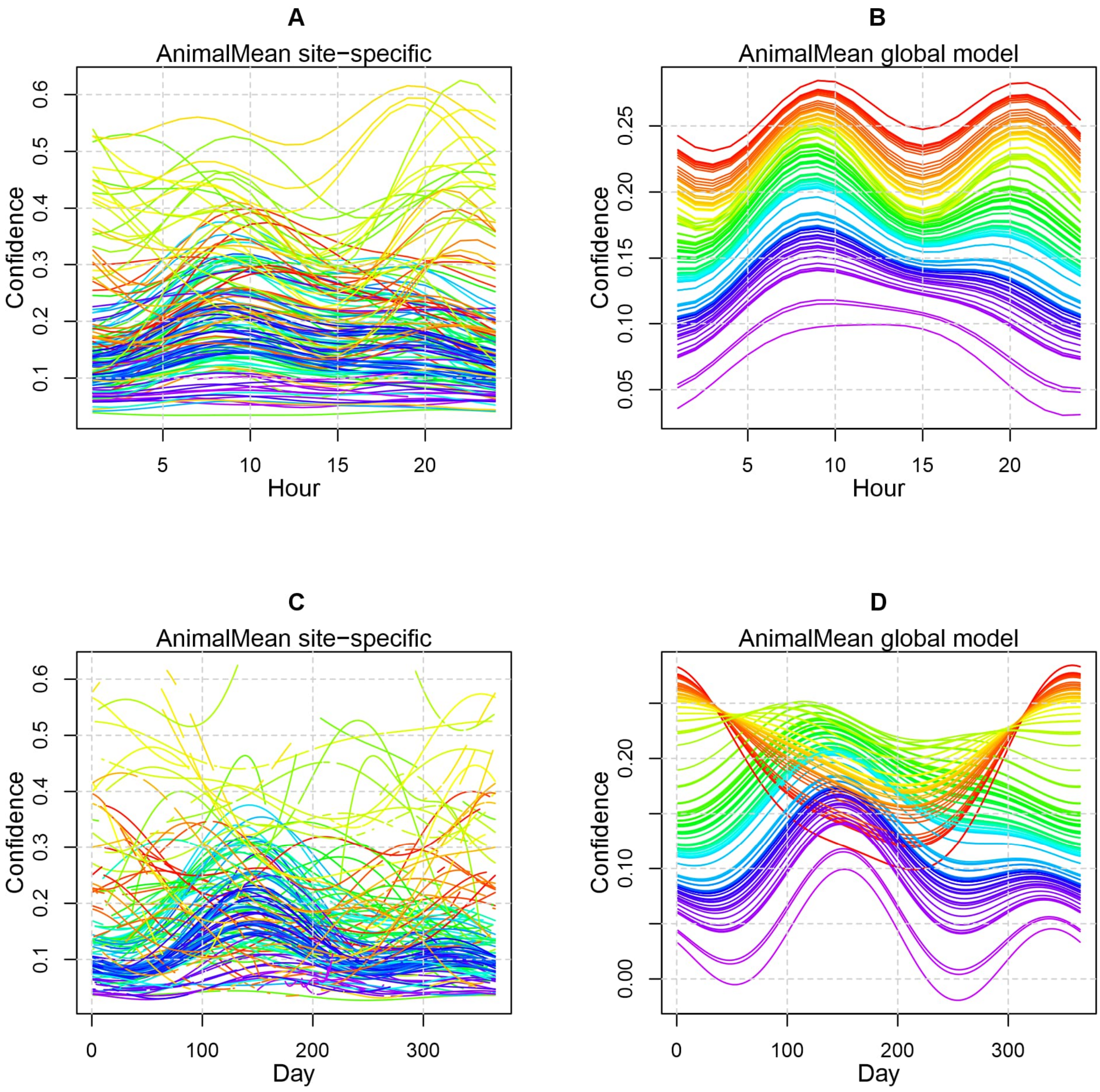

Supplementary Figure 43. Predictions of AnimalMean for the hour of the day (A, B) and the day of the year (C, D) for all sites in the study. Here, time is represented by time relative to the sun cycle, mapped so that sunrise corresponds to 6AM and sunset to 6PM (see Methods). Site-specific models are displayed on the left (A, C), with global model predictions on the right (B, D). Each site is color-coded according to the map in Supplementary Figure 1. All sites included have data from at least 100 days except for 3 sites for which the accessible field season is very short (locations in the extreme north).

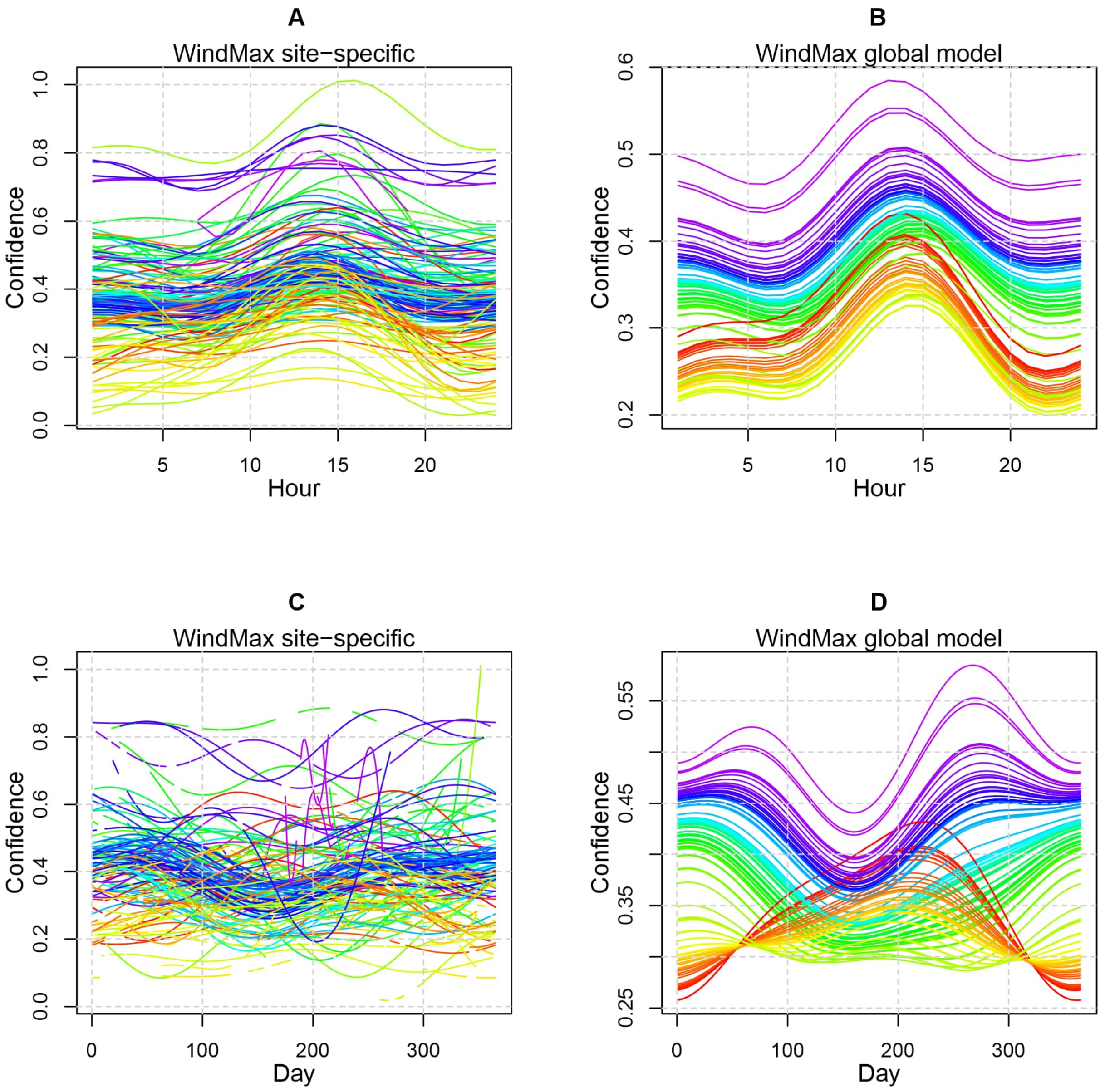

Supplementary Figure 44. Predictions of WindMax for the hour of the day (A, B) and the day of the year (C, D) for all sites in the study. Here, time is represented by time relative to the sun cycle, mapped so that sunrise corresponds to 6AM and sunset to 6PM (see Methods). Site-specific models are displayed on the left (A, C), with global model predictions on the right (B, D). Each site is color-coded according to the map in Supplementary Figure 1. All sites included have data from at least 100 days except for 3 sites for which the accessible field season is very short (locations in the extreme north).

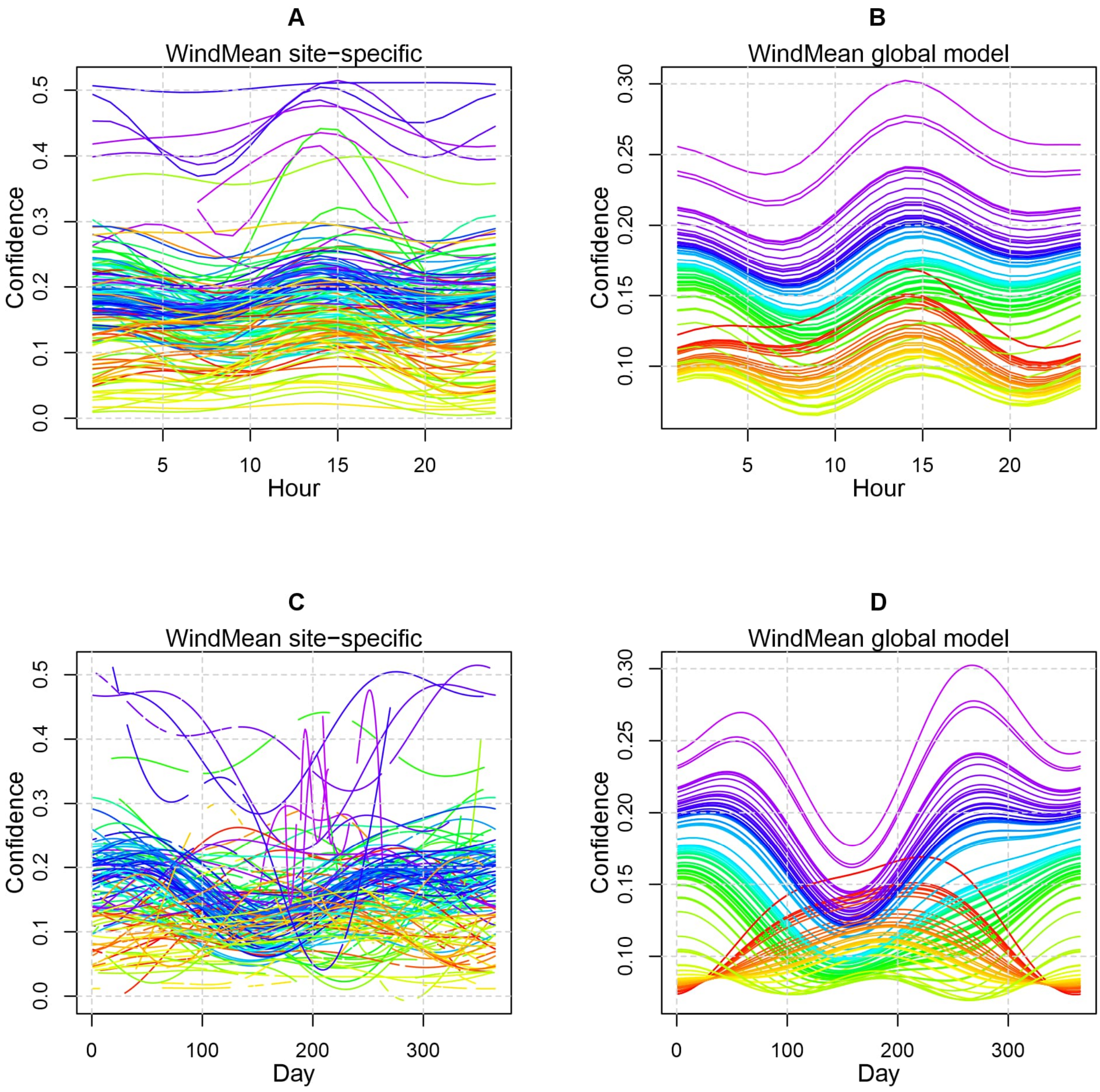

Supplementary Figure 45. Predictions of WindMean for the hour of the day (A, B) and the day of the year (C, D) for all sites in the study. Here, time is represented by time relative to the sun cycle, mapped so that sunrise corresponds to 6AM and sunset to 6PM (see Methods). Site-specific models are displayed on the left (A, C), with global model predictions on the right (B, D). Each site is color-coded according to the map in Supplementary Figure 1. All sites included have data from at least 100 days except for 3 sites for which the accessible field season is very short (locations in the extreme north).

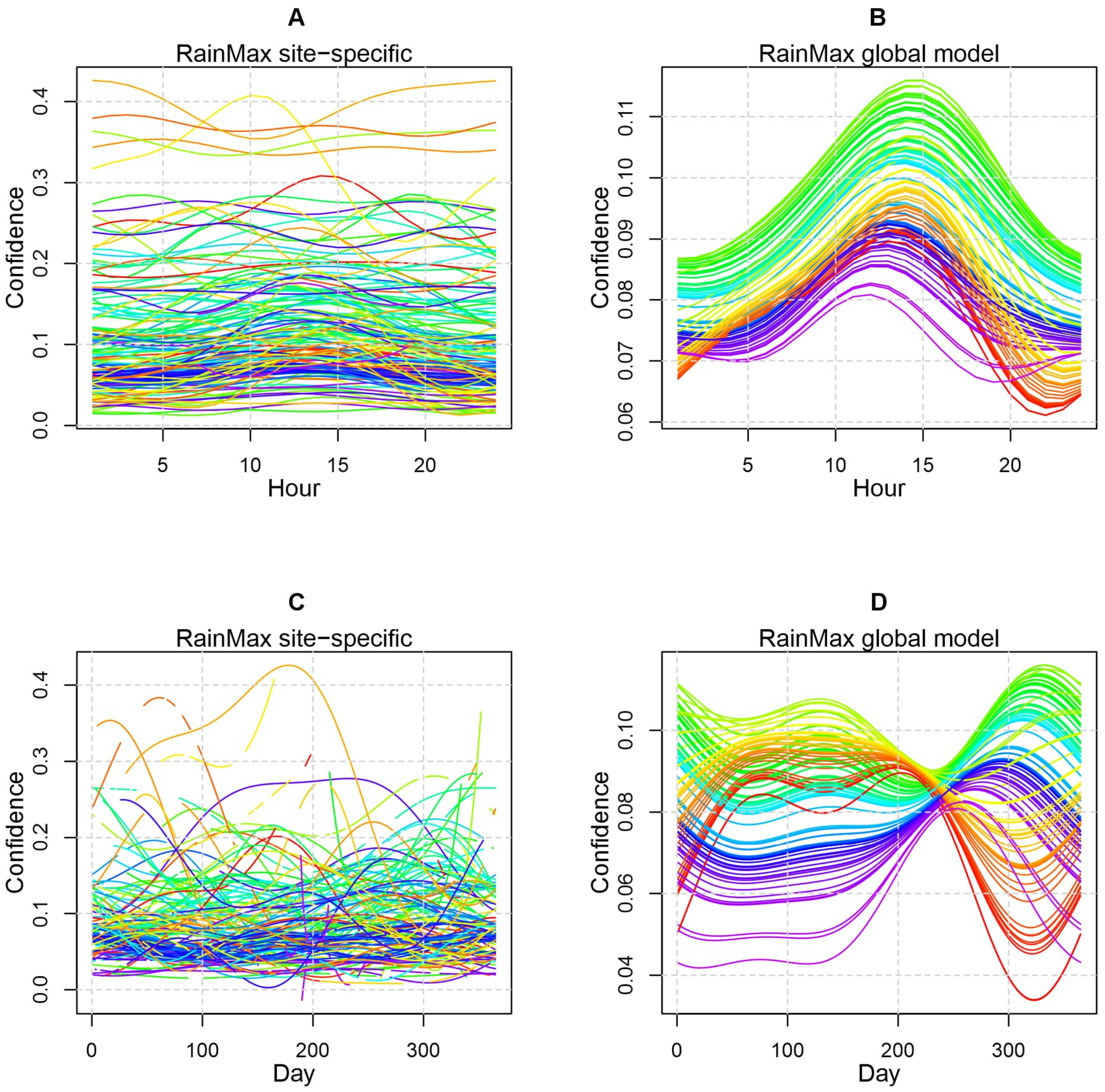

Supplementary Figure 46. Predictions of RainMax for the hour of the day (A, B) and the day of the year (C, D) for all sites in the study. Here, time is represented by time relative to the sun cycle, mapped so that sunrise corresponds to 6AM and sunset to 6PM (see Methods). Site-specific models are displayed on the left (A, C), with global model predictions on the right (B, D). Each site is color-coded according to the map in Supplementary Figure 1. All sites included have data from at least 100 days except for 3 sites for which the accessible field season is very short (locations in the extreme north).

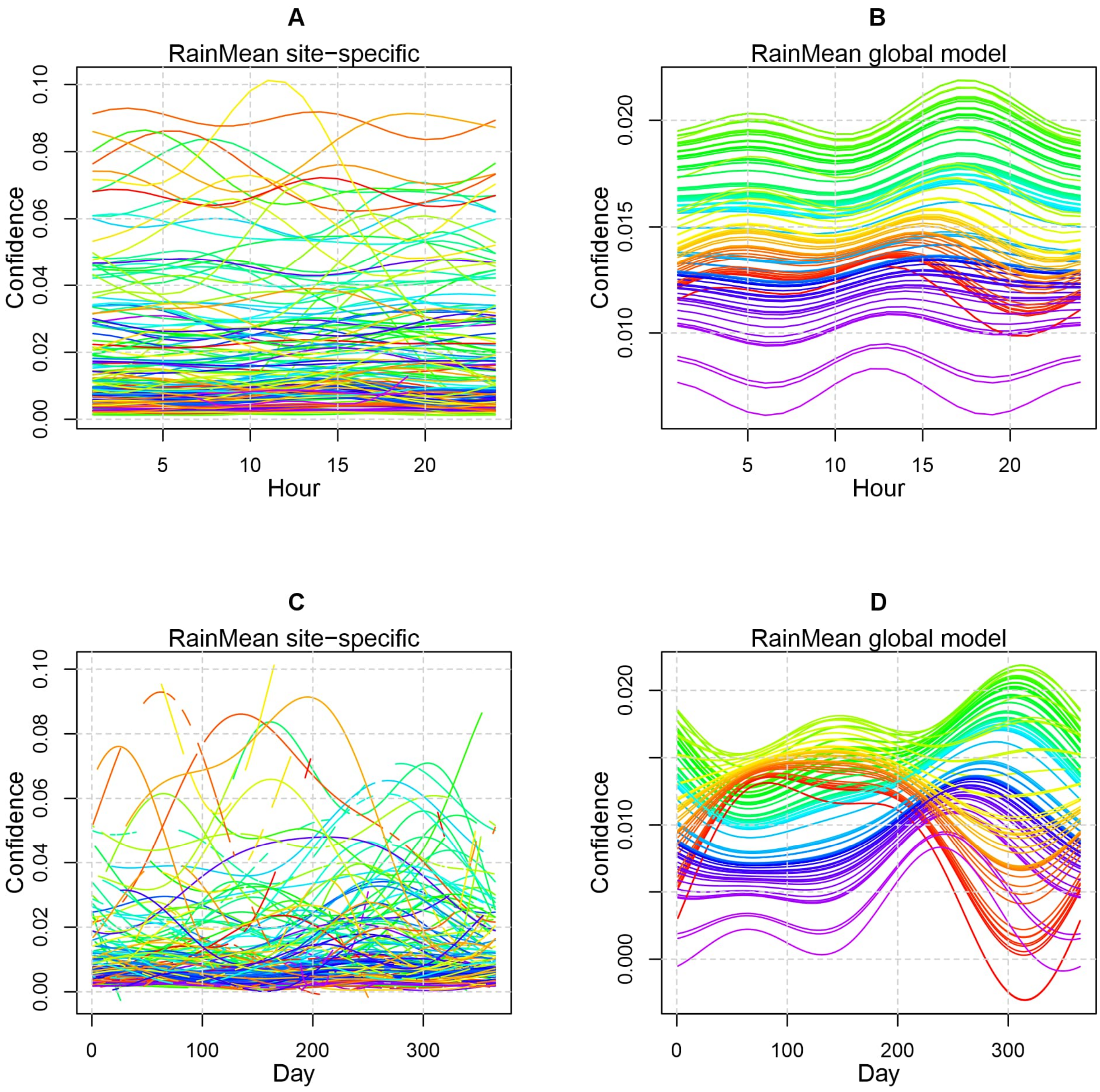

Supplementary Figure 47. Predictions of RainMean for the hour of the day (A, B) and the day of the year (C, D) for all sites in the study. Here, time is represented by time relative to the sun cycle, mapped so that sunrise corresponds to 6AM and sunset to 6PM (see Methods). Site-specific models are displayed on the left (A, C), with global model predictions on the right (B, D). Each site is color-coded according to the map in Supplementary Figure 1. All sites included have data from at least 100 days except for 3 sites for which the accessible field season is very short (locations in the extreme north).

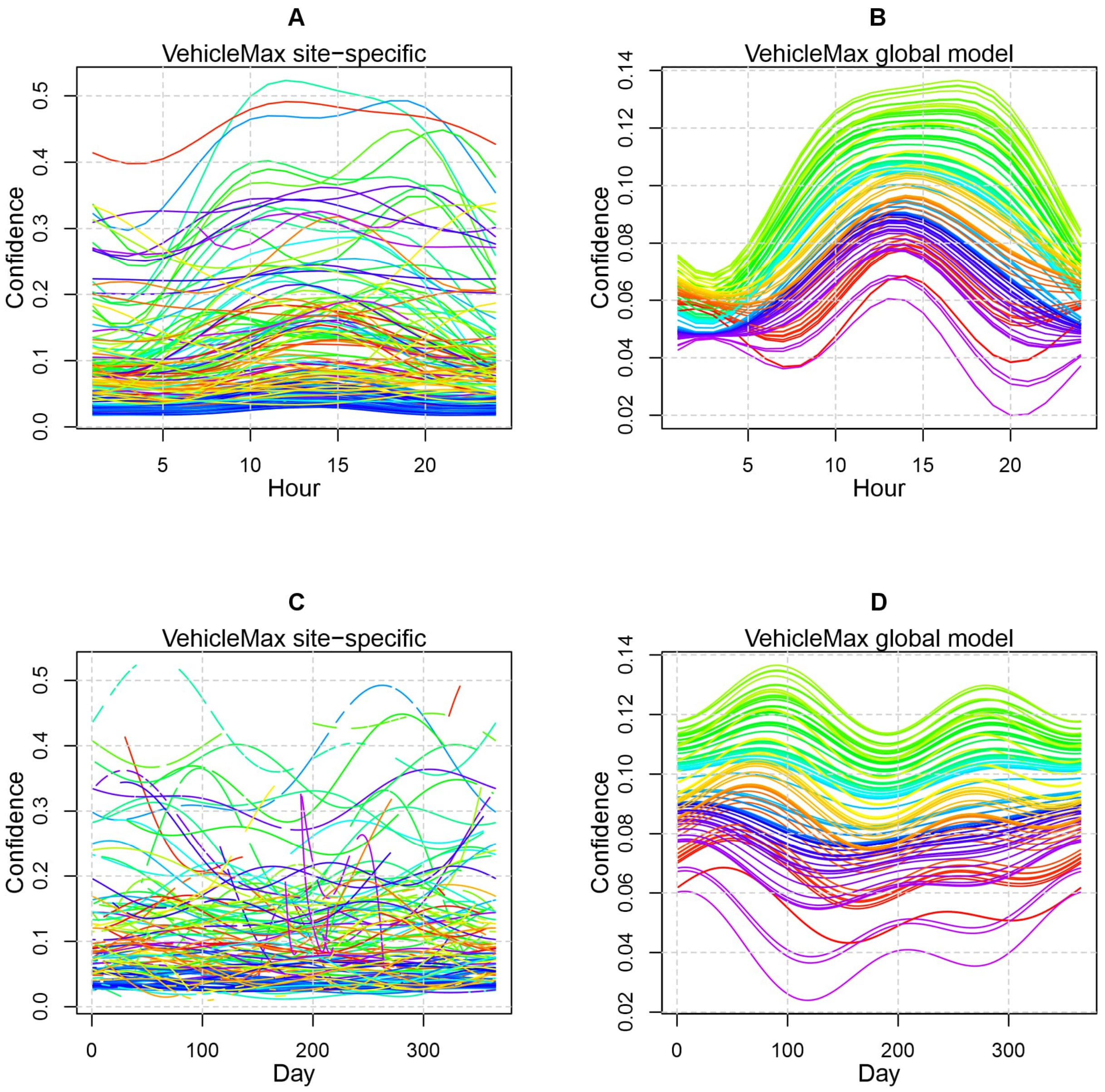

Supplementary Figure 48. Predictions of VehicleMax for the hour of the day (A, B) and the day of the year (C, D) for all sites in the study. Here, time is represented by time relative to the sun cycle, mapped so that sunrise corresponds to 6AM and sunset to 6PM (see Methods). Site-specific models are displayed on the left (A, C), with global model predictions on the right (B, D). Each site is color-coded according to the map in Supplementary Figure 1. All sites included have data from at least 100 days except for 3 sites for which the accessible field season is very short (locations in the extreme north).

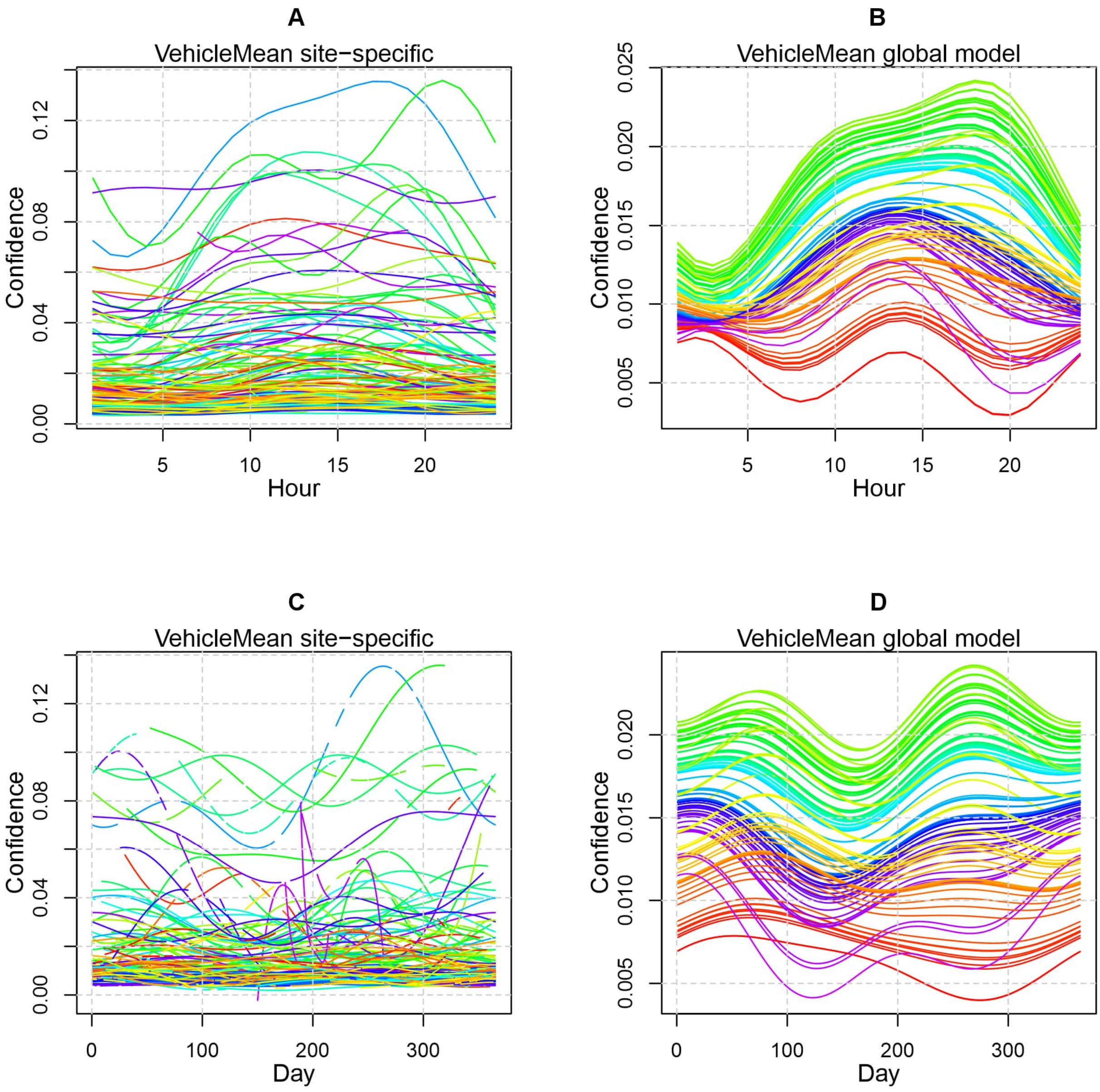

Supplementary Figure 49. Predictions of VehicleMean for the hour of the day (A, B) and the day of the year (C, D) for all sites in the study. Here, time is represented by time relative to the sun cycle, mapped so that sunrise corresponds to 6AM and sunset to 6PM (see Methods). Site-specific models are displayed on the left (A, C), with global model predictions on the right (B, D). Each site is color-coded according to the map in Supplementary Figure 1. All sites included have data from at least 100 days except for 3 sites for which the accessible field season is very short (locations in the extreme north).

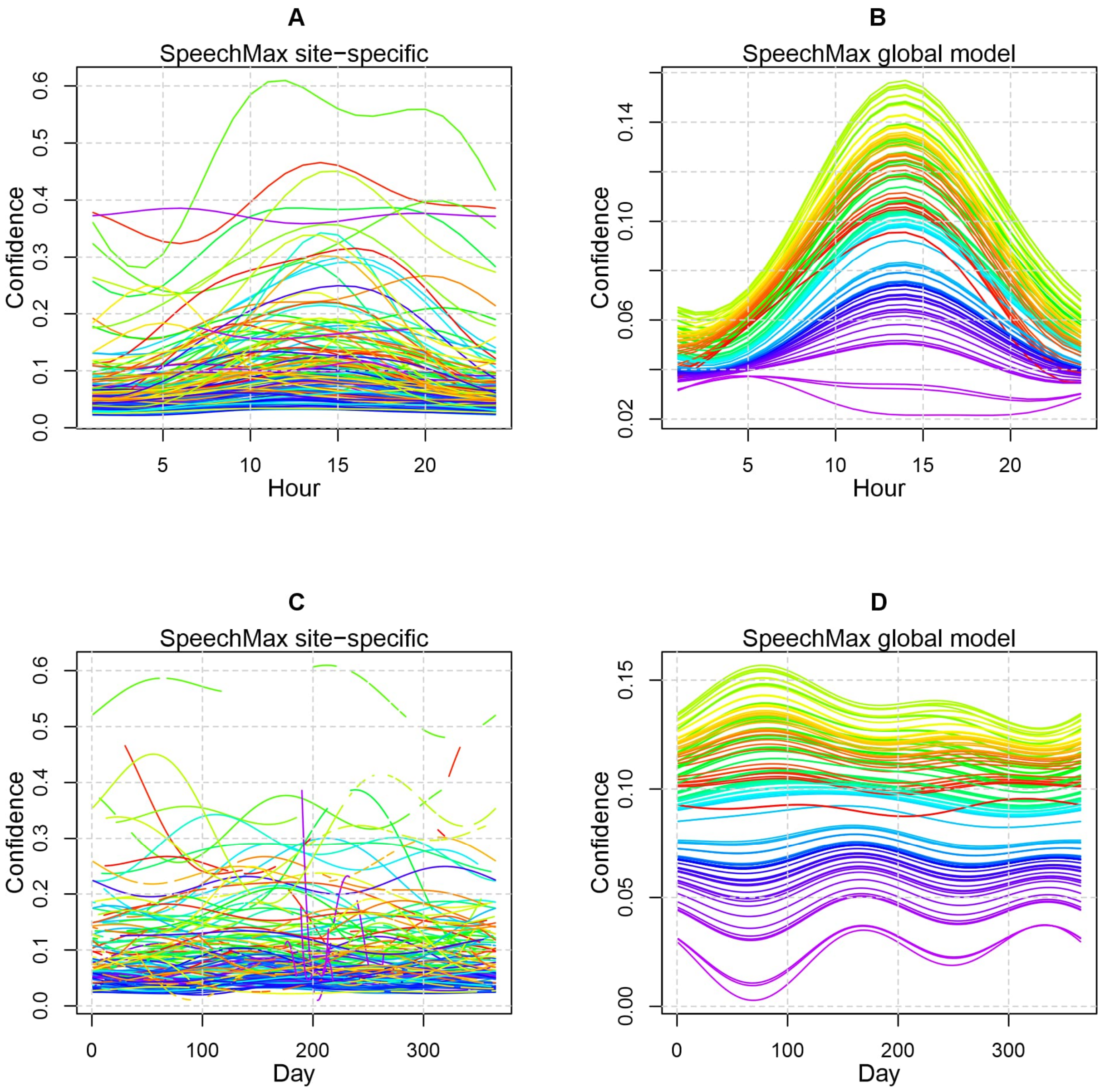

Supplementary Figure 50. Predictions of SpeechMax for the hour of the day (A, B) and the day of the year (C, D) for all sites in the study. Here, time is represented by time relative to the sun cycle, mapped so that sunrise corresponds to 6AM and sunset to 6PM (see Methods). Site-specific models are displayed on the left (A, C), with global model predictions on the right (B, D). Each site is color-coded according to the map in Supplementary Figure 1. All sites included have data from at least 100 days except for 3 sites for which the accessible field season is very short (locations in the extreme north).

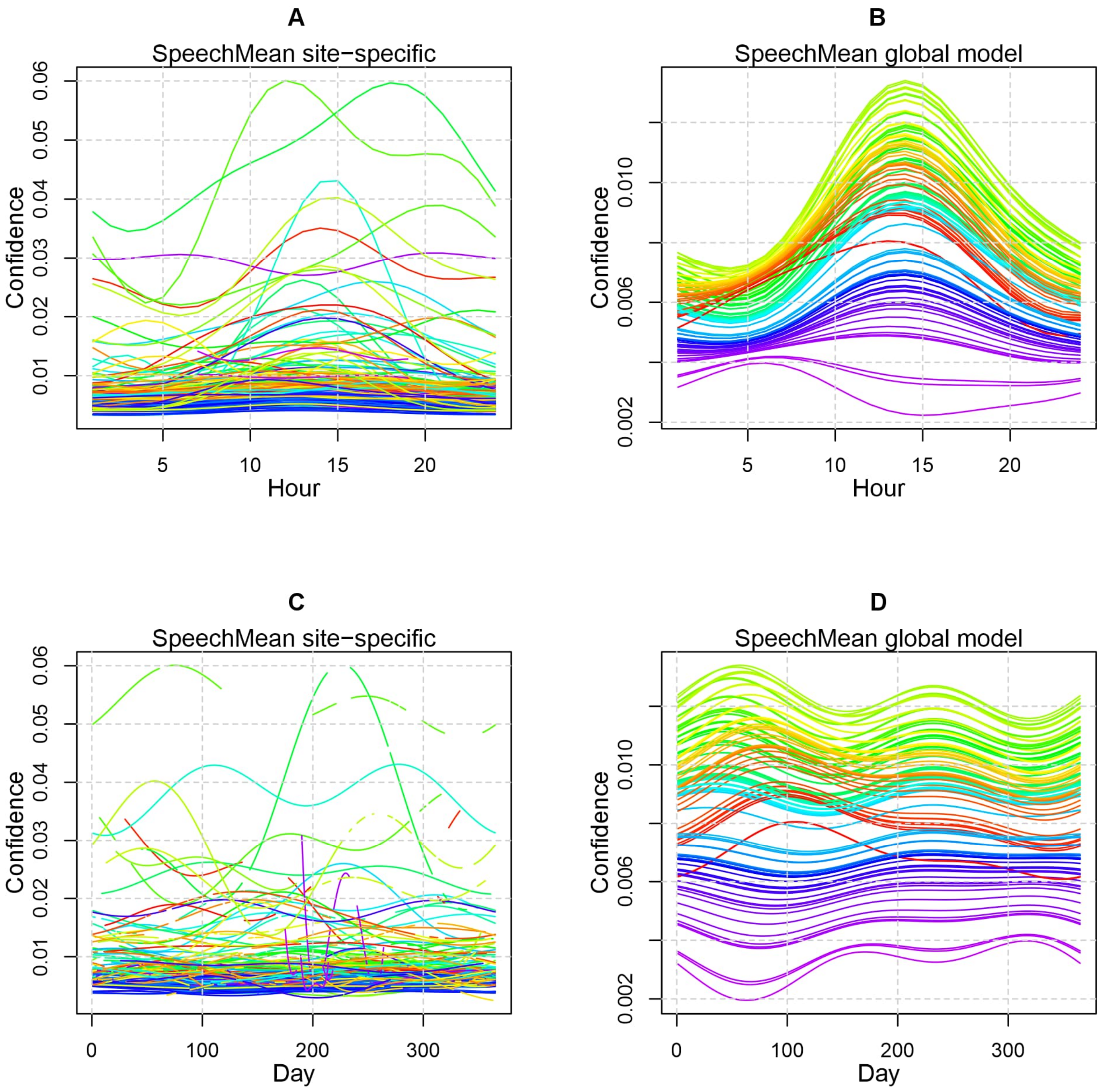

Supplementary Figure 51. Predictions of SpeechMean for the hour of the day (A, B) and the day of the year (C, D) for all sites in the study. Here, time is represented by time relative to the sun cycle, mapped so that sunrise corresponds to 6AM and sunset to 6PM (see Methods). Site-specific models are displayed on the left (A, C), with global model predictions on the right (B, D). Each site is color-coded according to the map in Supplementary Figure 1. All sites included have data from at least 100 days except for 3 sites for which the accessible field season is very short (locations in the extreme north).

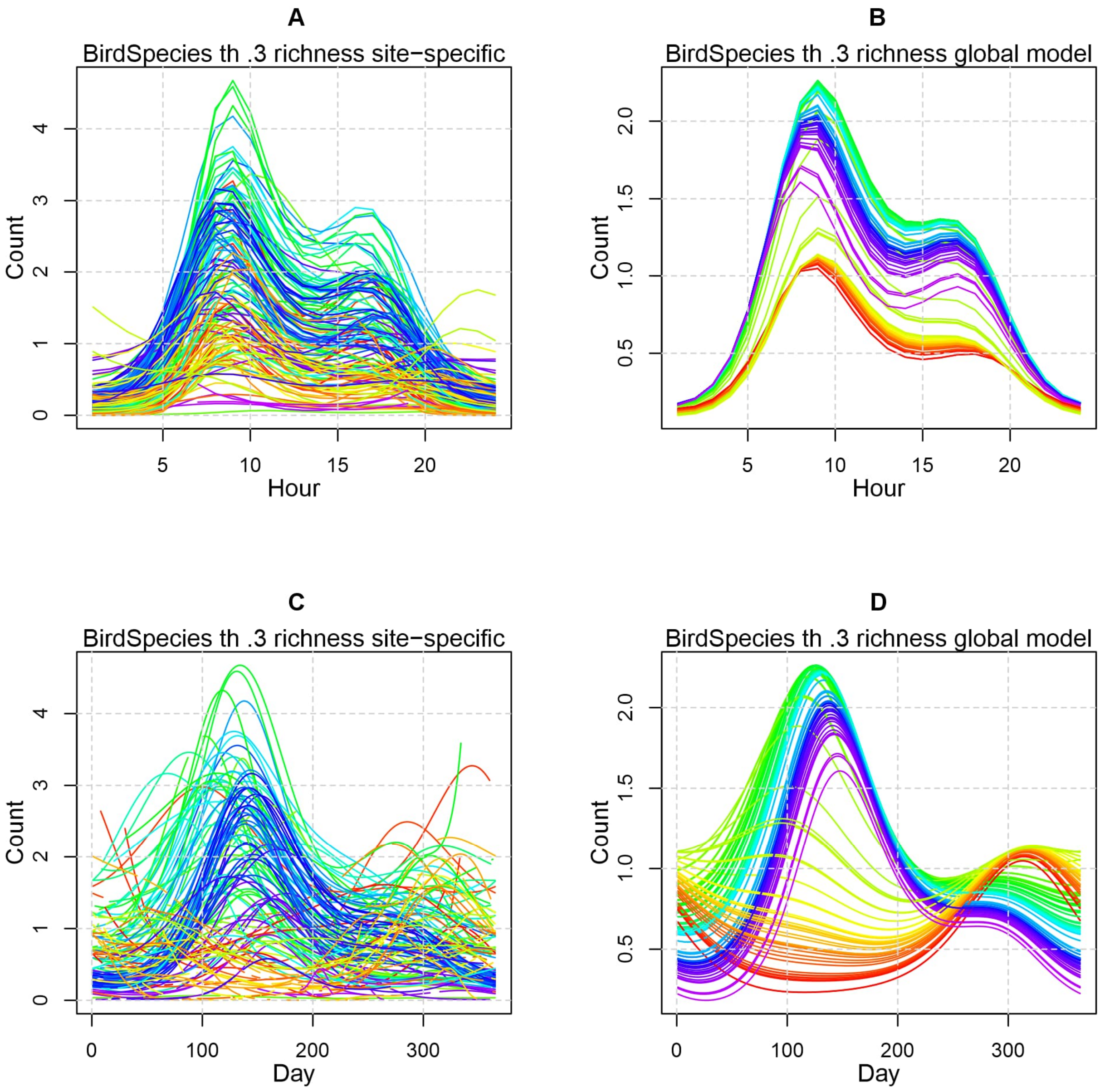

Supplementary Figure 52. Predictions of BirdSpecies with confidence threshold 0.3, measured as species richness, for the hour of the day (A, B) and the day of the year (C, D) for all sites in the study. Here, time is represented by time relative to the sun cycle, mapped so that sunrise corresponds to 6AM and sunset to 6PM (see Methods). Site-specific models are displayed on the left (A, C), with global model predictions on the right (B, D). Each site is color-coded according to the map in Supplementary Figure 1. All sites included have data from at least 100 days except for 3 sites for which the accessible field season is very short (locations in the extreme north).

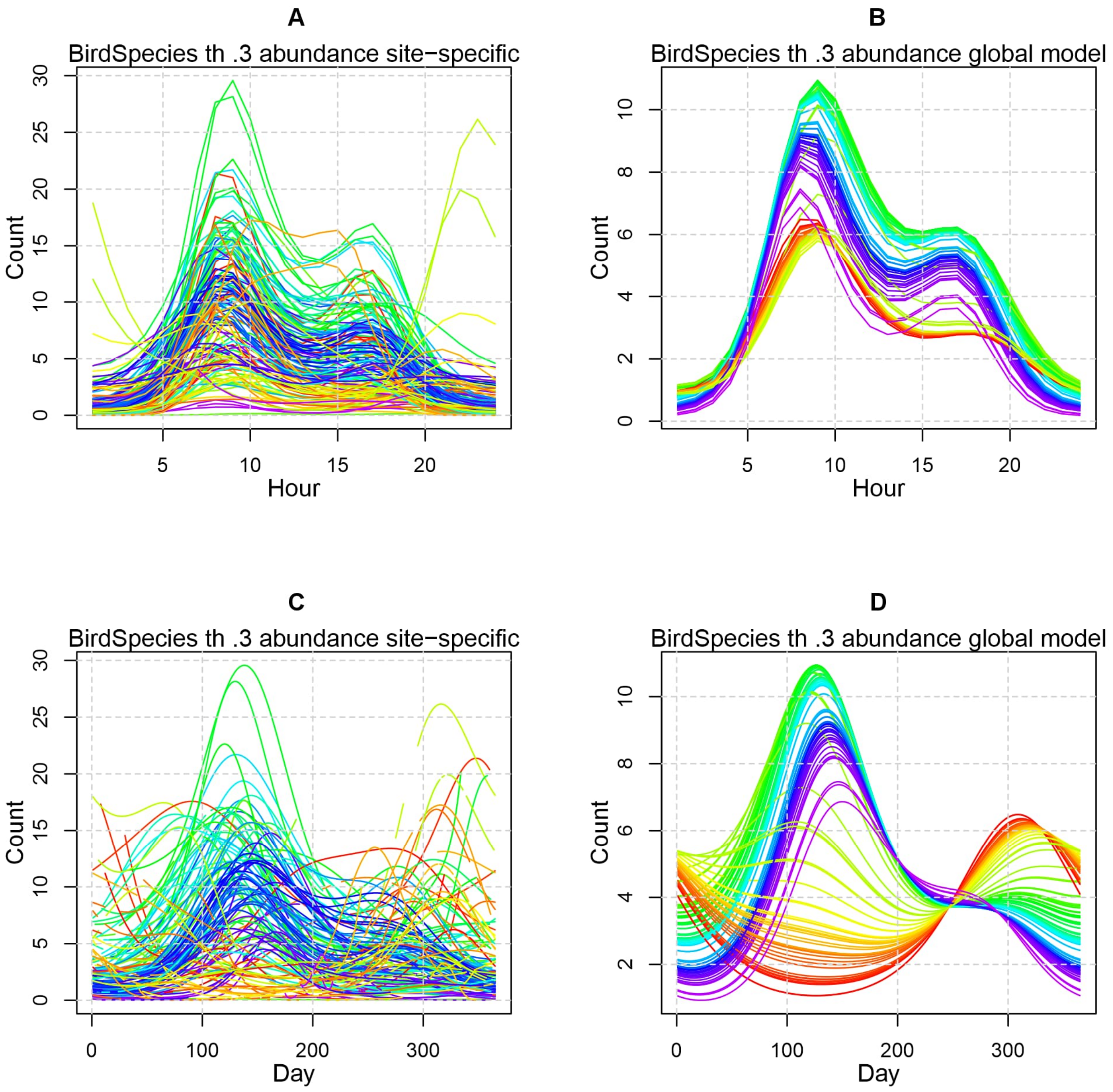

Supplementary Figure 53. Predictions of BirdSpecies with confidence threshold 0.3, measured as abundance, for the hour of the day (A, B) and the day of the year (C, D) for all sites in the study. Here, time is represented by time relative to the sun cycle, mapped so that sunrise corresponds to 6AM and sunset to 6PM (see Methods). Site-specific models are displayed on the left (A, C), with global model predictions on the right (B, D). Each site is color-coded according to the map in Supplementary Figure 1. All sites included have data from at least 100 days except for 3 sites for which the accessible field season is very short (locations in the extreme north).

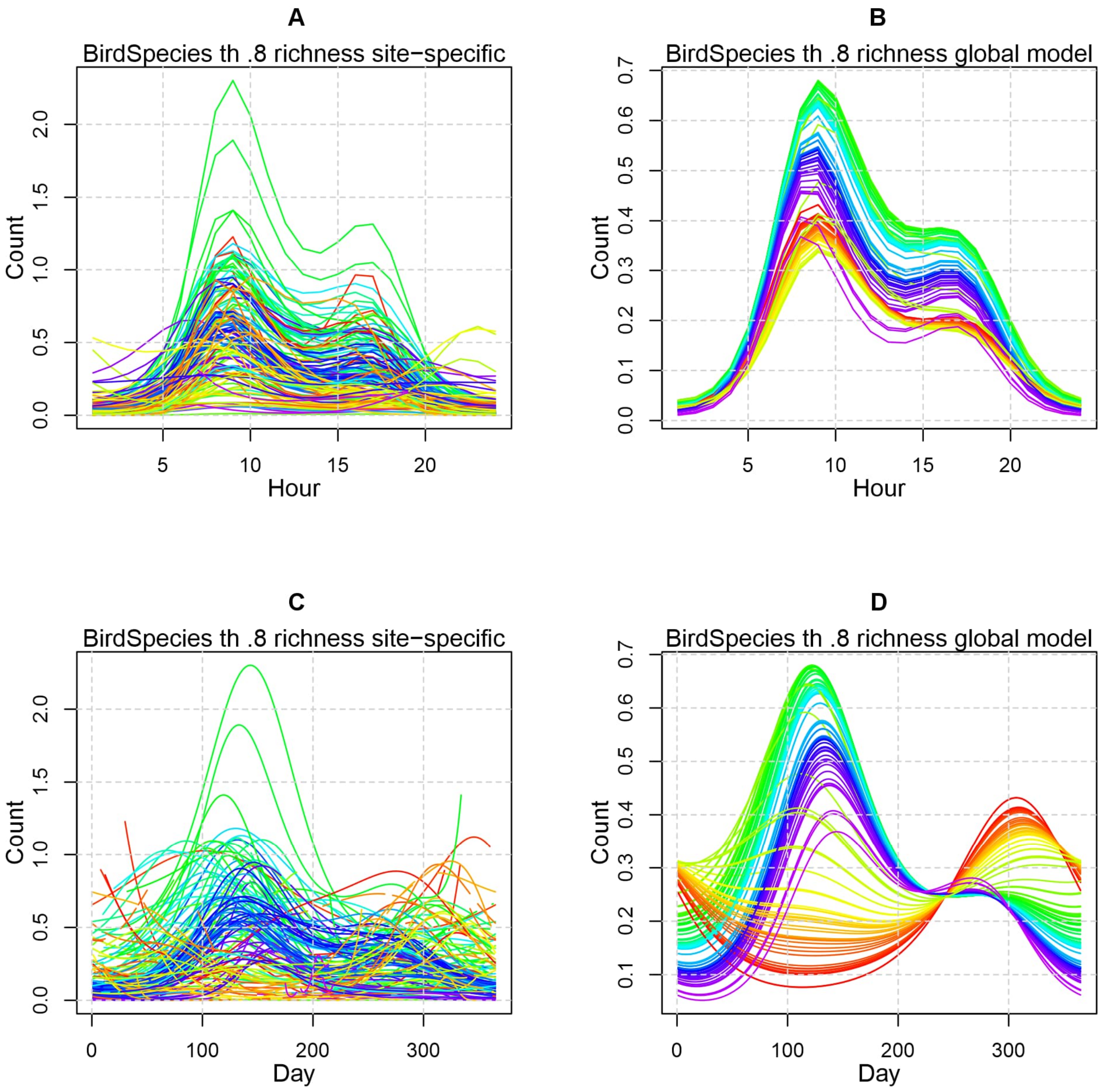

Supplementary Figure 54. Predictions of BirdSpecies with confidence threshold 0.8, measured as species richness, for the hour of the day (A, B) and the day of the year (C, D) for all sites in the study. Here, time is represented by time relative to the sun cycle, mapped so that sunrise corresponds to 6AM and sunset to 6PM (see Methods). Site-specific models are displayed on the left (A, C), with global model predictions on the right (B, D). Each site is color-coded according to the map in Supplementary Figure 1. All sites included have data from at least 100 days except for 3 sites for which the accessible field season is very short (locations in the extreme north).

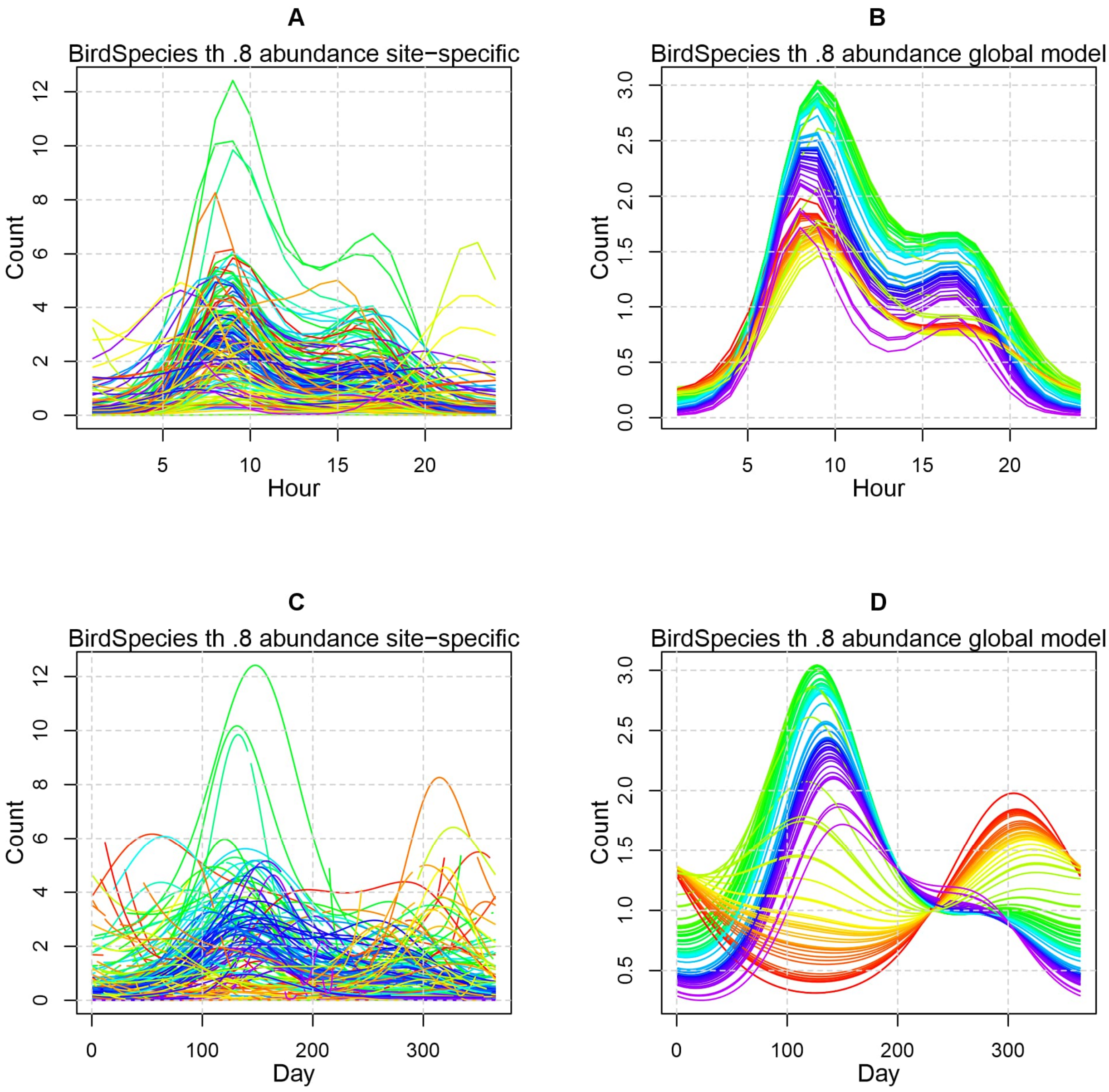

Supplementary Figure 55. Predictions of BirdSpecies with confidence threshold 0.8, measured as abundance, for the hour of the day (A, B) and the day of the year (C, D) for all sites in the study. Here, time is represented by time relative to the sun cycle, mapped so that sunrise corresponds to 6AM and sunset to 6PM (see Methods). Site-specific models are displayed on the left (A, C), with global model predictions on the right (B, D). Each site is color-coded according to the map in Supplementary Figure 1. All sites included have data from at least 100 days except for 3 sites for which the accessible field season is very short (locations in the extreme north).

Section 2. Composition of sounds under index “Animal”

To evaluate the composition of sounds classified by YAMNet[1] as “animals”, we drew on YAMNet[1] classification of audio events into three nested subclasses under “Animal”: 1) domestic animals and pets; 2) livestock, farm animals and working animals; and 3) wild animals. For each recording where an Animal event was detected with high confidence (>0.9; n=134,877), we examined the confidence with which the event could be further assigned to a given subclass. As will be evident from Supplementary Figure 56, most events could be reliably assigned to wild animals.

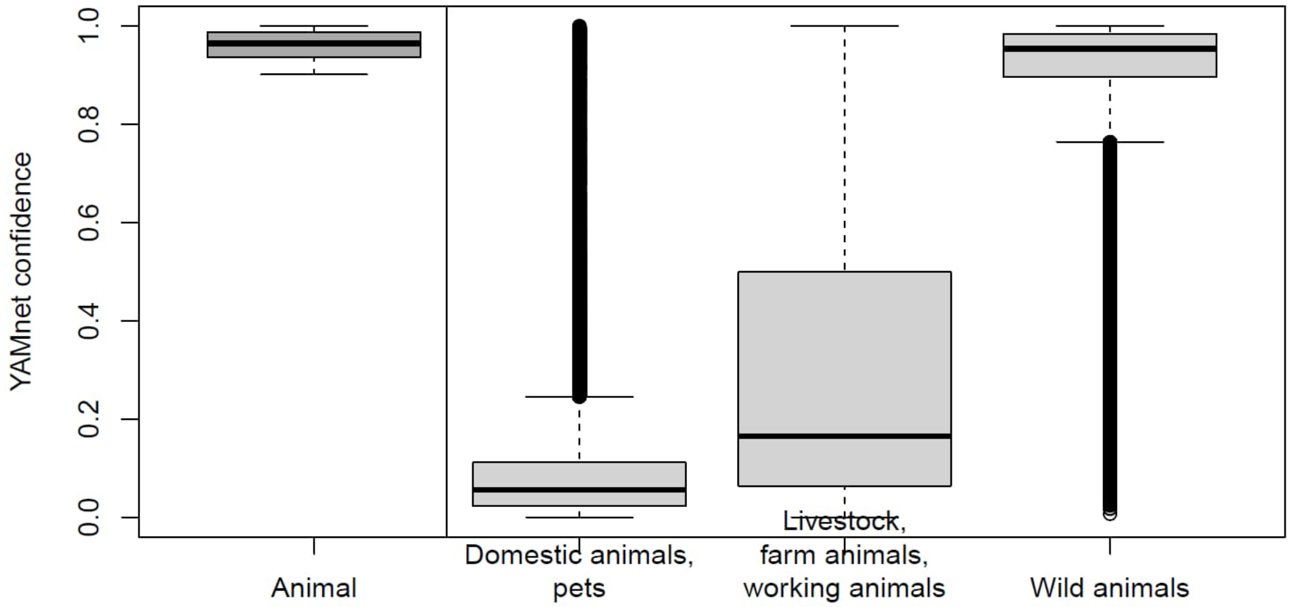

Supplementary Figure 56. Composition of sounds under index “Animal”, as split into subclasses “domestic animals and pets”; “livestock, farm animals and working animals”; and “wild animals”.

Section 3. Evaluating the effect of time representation on model predictions and inference

In models of diurnal patterns, time can be represented in two different ways: as local absolute time and as time relative to local sunrise and sunset. To test for differences in the patterns detected, we fitted local and global regression models using both time representations. As explained in the Methods, we obtained the time relative to the sun cycle by converting the original time stamp on the recording to a new time based on the information of the sunrise and sunset at the recording site, obtained using R package suncalc, version 0.5.1. The hour of the day was mapped so that the sunrise corresponded to 6AM and sunset to 6PM. For the time between the sunrise and sunset (daytime), local absolute hours were mapped linearly between 6:00AM and 6:00PM, and for the time between the sunset and sunrise (nighttime), the local absolute hours were mapped linearly between 6PM and 6AM.

To illustrate the consistency in the results obtained, we here reproduce two of the Results figures from the main text, where we used absolute hour as a representation of the time of day. In Supplementary Figure 57 and Supplementary Figure 58, we show the results from otherwise identical models but using time relative to sun set and sun rise. As will be evident from a comparison between Fig. 3 in the main text and Supplementary Figure 57 below, and between Fig. 4 of the main text and Supplementary Figure 58 below, the patterns recovered are virtually identical and our general inference thus robust with respect to the definition of time used.

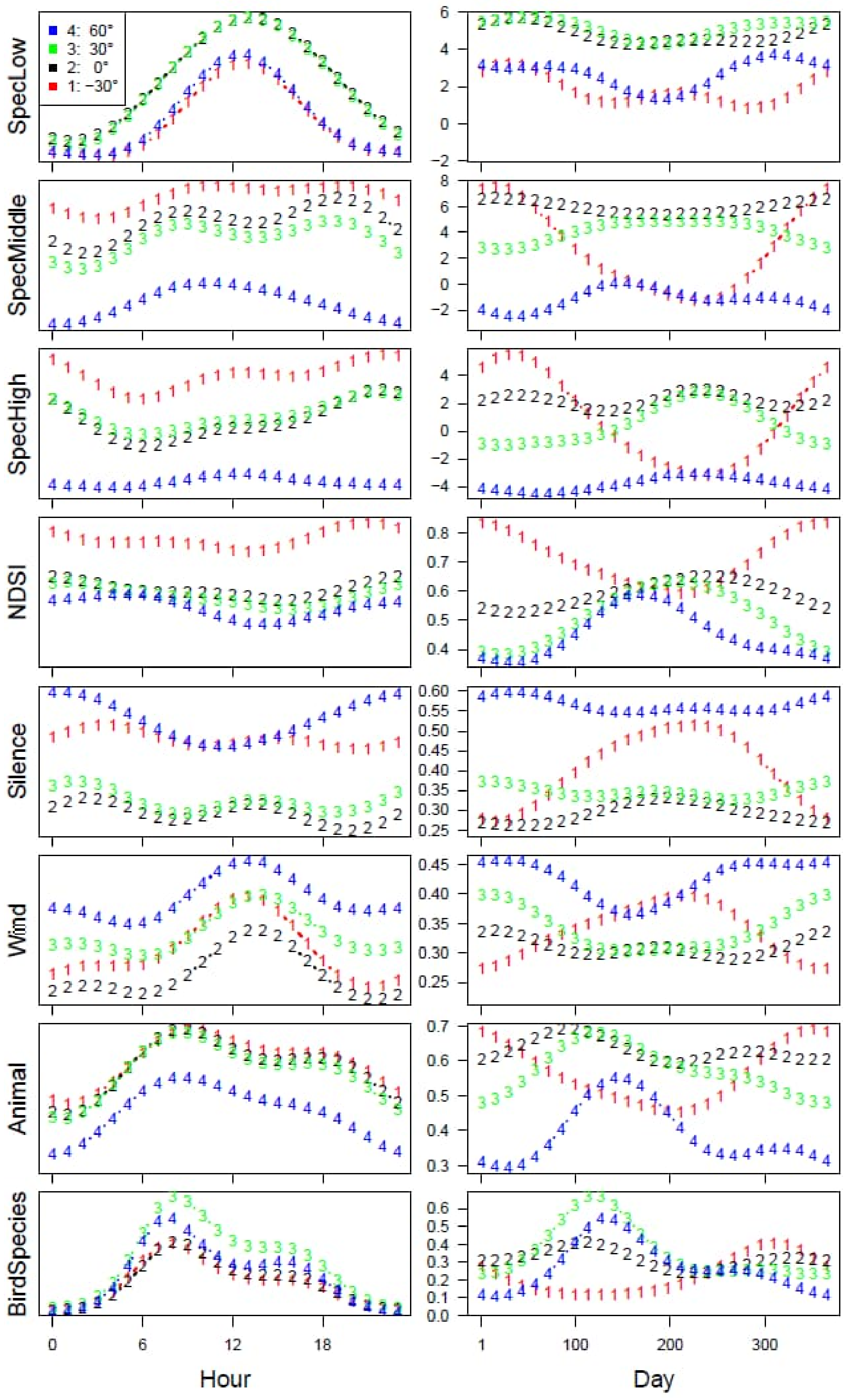

Supplementary Figure 57. Fig. 3 of the main text reproduced for an alternative definition of the local hour – here scaled as time relative to local sun rise and sun set. Shown is seasonal and diurnal variation in different acoustic indices (for definitions of each index, see Fig. 2 in main text). Panels on the left-hand side show predictions for the hour of the day and panels on the right-hand side show the predictions for the day of the year for -30°, 0°, 30° and 60° degrees of northern latitude. The predictions shown are based on the

global model fitted to all data and shown for those eight indices for which the seasonal and diurnal patterns explained a substantial part of the variation (Fig. 4 in main text)

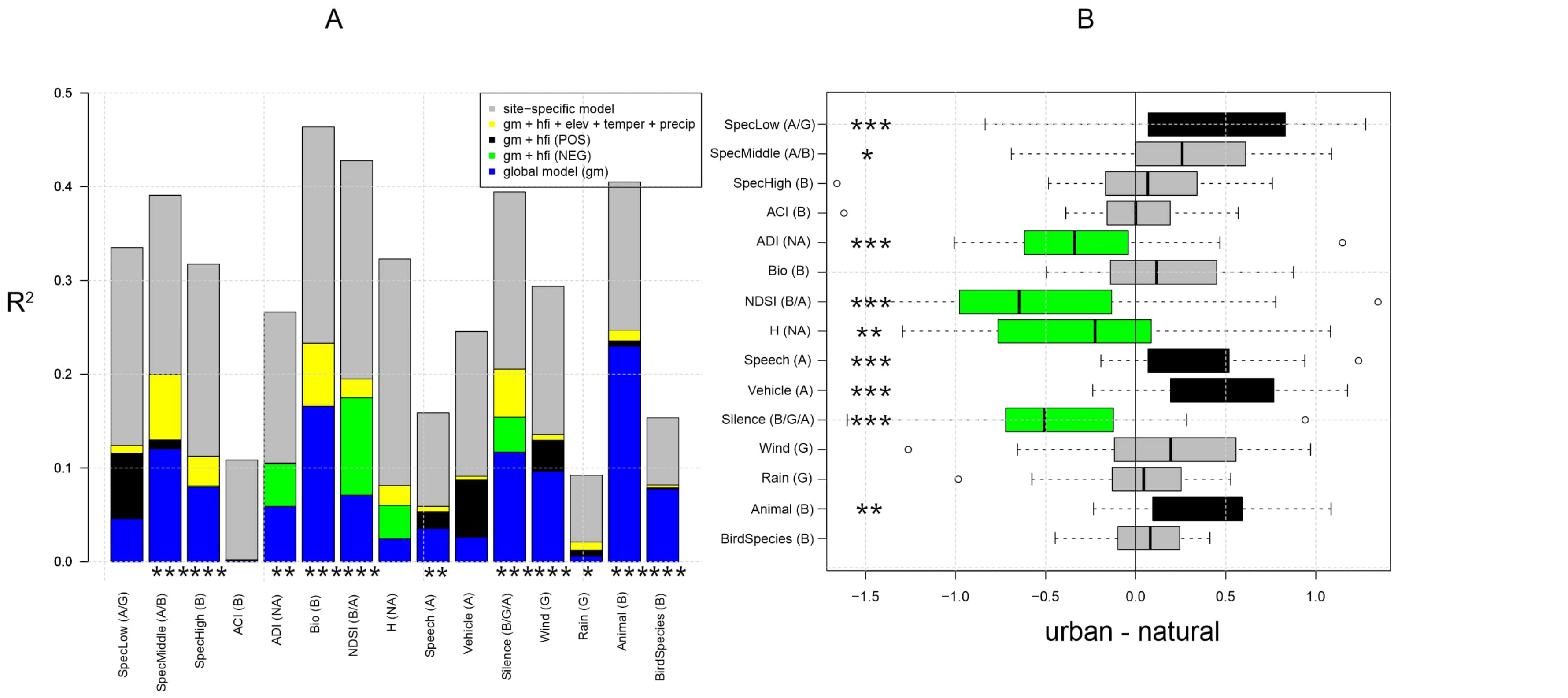

Supplementary Figure 58. Fig. 4 of the main text reproduced for an alternative definition of the local hour – here scaled as time relative to local sun rise and sun set. A) Predictability of 15 acoustic indices in space and time. Note that indices are sorted by their type (i.e. how they are calculated from the spectrogram), whereas letters underneath each column clarify how they are assigned to B=Biophony; G=Geophony; A=Anthropophony; or NA= No clear classification. The total height of each bar shows the proportion of variance explained (R^2) by site-specific models applied to the global data – i.e. the how much unexplained variation remains ($1-R^2$) in the global data after site-specific models are used to predict local patterns. The blue part of the bars show how much of the variation is explained by the global model where the only information of each site is the latitude. Asterisks show the significance of latitude in the global model, with a P-value <0.05 denoted by one asterisk, <0.01 by two and <0.001 by three asterisks. Black and green bars show how much R^2

increases when the human footprint index of a site is included in a global model. Black colour denotes positive effect and green colour denotes negative effect. The yellow bar indicates how much R^2 increases when climatic conditions (elevation, mean annual temperature, and precipitation) are added to a model already including the human footprint index. Panel B) shows differences in soundscapes observed across pairs of natural vs. urban sites. Each box shows the distribution of empirically-observed pairwise differences between the two sites within a pair, and thus the consistency of differences between natural vs. urban sites. The box shows the interquartile (50% of data) and the thick vertical line inside the box is the median. Whiskers show the minimum and maximum up to 1.5 times the interquartile range from the box and individual data points show outliers beyond this range. Since the difference is calculated as urban minus natural values, a positive value indicates higher values for urban sites, whereas a negative value will indicate higher values at natural sites. Stars show the statistical significance of the difference based on t-test, with a P-value <0.05 denoted by one asterisk, <0.01 by two and <0.001 by three asterisks. Black and green colours indicate cases for which the P-value was below 0.01, for direct comparison with panel A.

Section 4. Correlations between different soundscape indices

To quantify the level of congruence among individual soundscape indices, we calculated the Spearman rank correlation coefficients between all 15 soundscape indices. As will be evident from Supplementary Figure 59, correlations ranged from very high to virtually zero, supporting the view that different indices characterise different features of the soundscape, and that indices are mutually complementary rather than alternative.

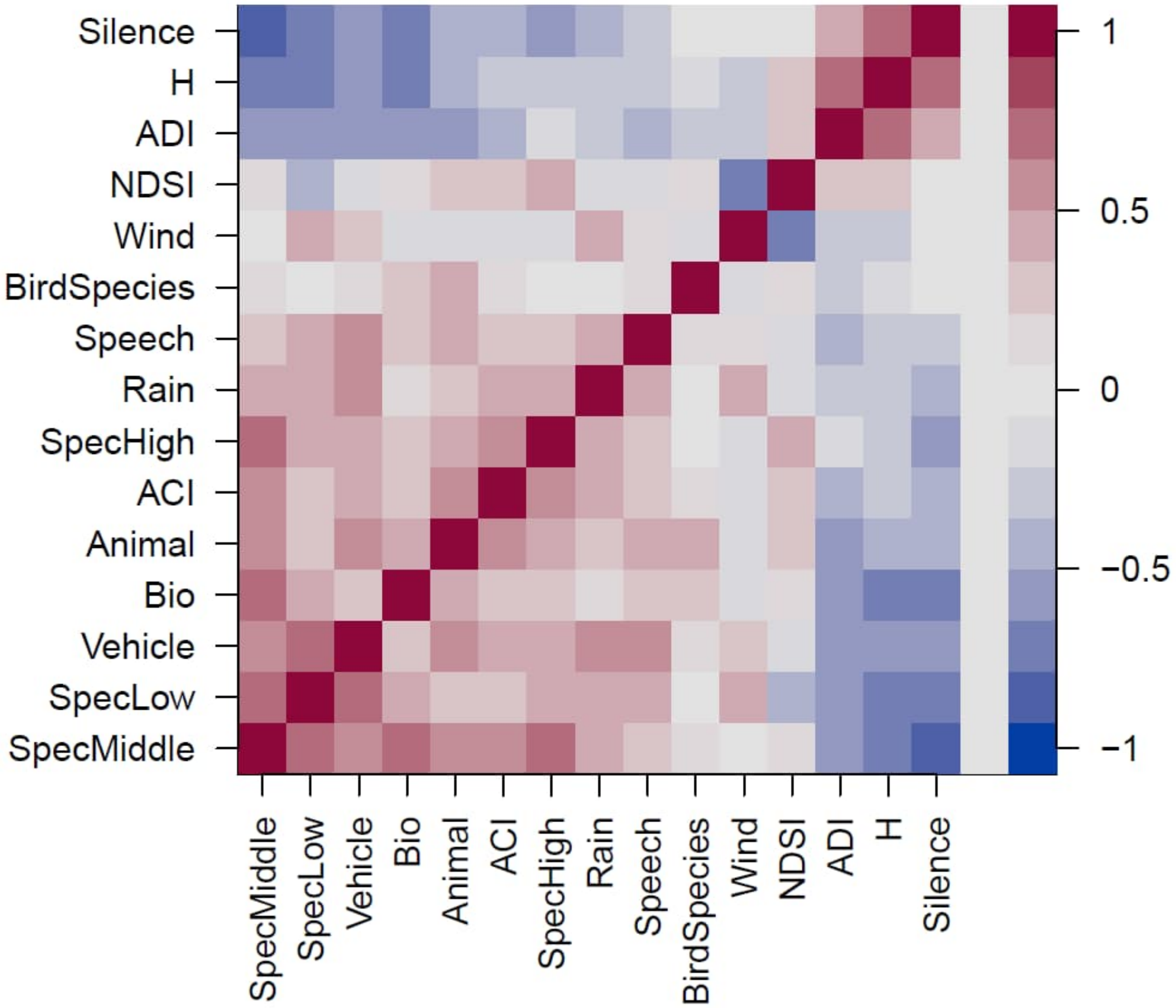

Supplementary Figure 59. Correlations between different soundscape indices

Correlations between 15 soundscape indices. Shown are the values of Spearman correlation coefficients. For a short description of each metric see Fig. 1 in the main text.

Section 5. Validation of BirdNET detections

To validate the classification of sounds to individual bird species, we asked an experienced ornithologist to listen through a material of 615 selected 3-second sound clips from two natural and two urban sites – while annotating all sounds detected. The clips were selected from four sites (two urban and two natural sites) so that from each site as many species as possible detected by BirdNET[2] were included. For each species, the 3-second clip giving the highest confidence for each species (minimum confidence being 0.1 and maximum 1.0) was selected. For each 3-second clip, a 1-second left margin and right margin was added for the human listener. The ornithologist annotated all the species that he was able to identify in each of the sound clips. The BirdNET[1] identification was considered correct if the output coincided with the species identification of this ornithologist. Based on the material of 615 selected 3-second sound clips, BirdNET gives 60% false negatives using confidence threshold 0.3 and 70% false negatives using confidence threshold of 0.8. The results summarised in Supplementary Figure 60 are comparable with the results of [3] (Fig. 4 therein that indicates false negative rate at least 80% with confidence threshold 0.55 and false negative rate at least 85% with confidence threshold 0.65).

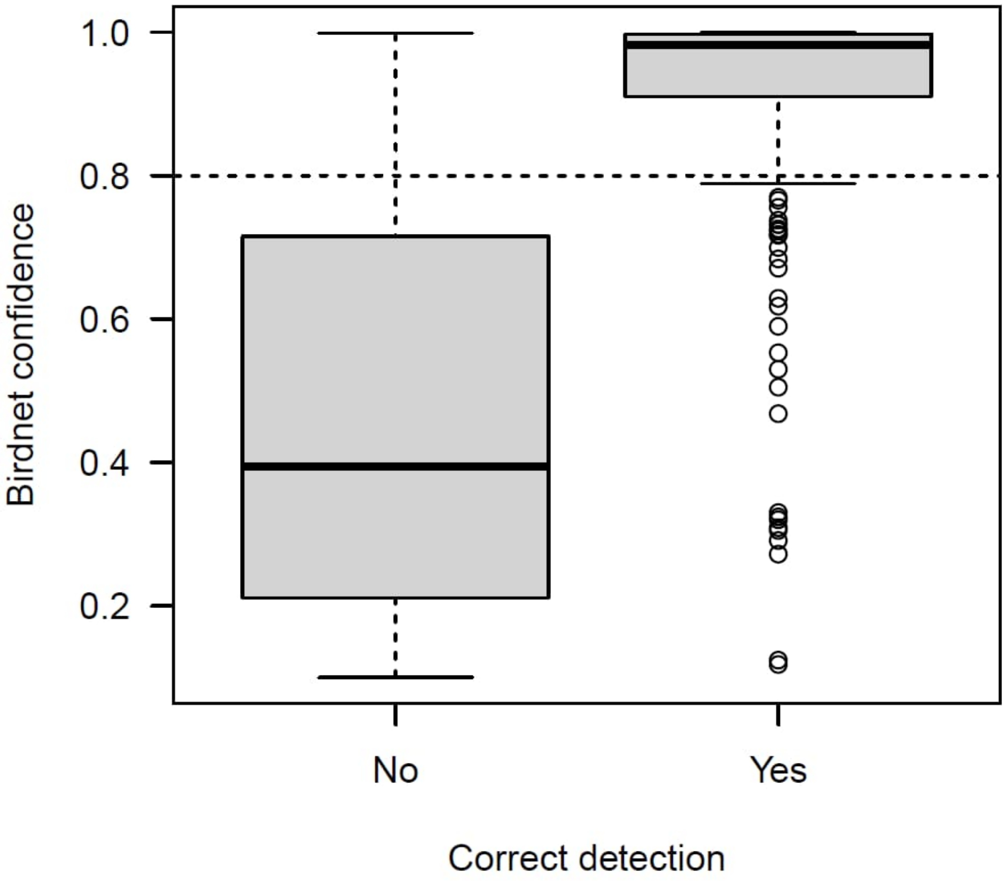

Supplementary Figure 60. Validation of BirdNET detections.
The figure shows differences in the confidence associated with the original identification by Birdnet[1], as split into identifications scored as being incorrect (X=NO) or correct (X=YES) by a human ornithologist. The dashed horizontal lines correspond to the confidence threshold of 0.8 used in the present study. A full list of manual annotations is offered in Supplement 6. The box shows the interquartile (50% of data) and the thick vertical line inside the box is the median. Whiskers show the minimum and maximum up to 1.5 times the interquartile range from the box and individual data points show outliers beyond this range.

To further analyze BirdNET results, we calculated sensitivity and specificity for its detections. Manual annotations served as ground truth where out of 615 sound clips, in 144 there were no birds based on human listener, in 225 clips there were a single species, and in 246 clips there were more than one species present. We calculated sensitivity and specificity for two cases, 1) single species and 2) more than one species present (based on human listener) and in both of them using two values (0.3 and 0.8) for BirdNET confidences as detection thresholds. When calculating the statistics, we used all BirdNET detections above the thresholds within the short clips, not only the species that gave the highest confidence. For the single species case, the sensitivities were 61% and 47% and the specificities were 33% and 81% for confidence thresholds 0.3 and 0.8, respectively. For the multi-species case, the sensitivities were 33% and 18% and the specificities were 30% and 78% for confidence thresholds 0.3 and 0.8, respectively. This indicates that BirdNET misses more detections when there are multiple species singing simultaneously (lower sensitivity for multi-species cases compared to single-species case). The effect of confidence thresholds is as expected, lower threshold increases sensitivity but reduces specificity whereas higher confidence threshold decreases sensitivity and increases specificity.

Section 6. Species and trait compositions in urban green spaces vs. more pristine sites

To test for differences in species and trait compositions between urban green spaces and more pristine sites, we used database AVONET[4] to assign information on species ecology and habitat use. Categorical traits as described in AVONET metadata are:

Habitat (Desert = drylands and other open arid habitats, often sandy with very sparse vegetation; Rock = rocky substrate typically with no or very little vegetation, including rocky outcrops, rocky coastlines, arid stony steppes, rocky mountaintops and mountain slopes; Grassland = open dry to moist grass-dominated landscapes, at all elevations; Shrubland = low stature bushy habitats, included thornscrub, thorny or arid savanna, caatinga, xerophytic shrubland and coastal scrub; Woodland = medium stature tree-dominated habitats, including Acacia woodland, riparian woodlands, mangrove forests, forest edges, also more open parkland with scattered taller trees; Forest = tall tree-dominated vegetation with more or less closed canopy, including palm forest; Human modified = urban landscapes, intensive agriculture, gardens; Wetland = wide range of freshwater aquatic habitats including lakes, marshes, swamps and reedbeds; Riverine = associated with rivers and streams at all elevations; Coastal = intertidal zones within immediate vicinity of beaches, estuaries, brackish to salty marshes, including mudflats, lagoons, alkaline wetlands, coastal dunes and harbours; Marine = pelagic, on sea near coasts, including species in the intertidal zone on beaches, and those pelagic species nesting near the sea on cliffs, islets and islands).

Habitat.Density (1 = Dense habitats. Species primarily lives in the lower or middle storey of forest, or in dense thickets, dense shrubland etc.; 2 = Semi-open habitats. Species primarily lives in open shrubland, scattered bushes, parkland, low dry or deciduous forest, thorn forest; 3 = Open habitats. Species primarily lives in desert, grassland, open water, low shrubs, rocky habitats, seashores, cities. Also applies to species living mainly on top of forest canopy (i.e. mostly in the open))

Migration (1 = Sedentary. 2 = Partially migratory, i.e. minority of population migrates long distances, or most of population undergoes short-distance migration, nomadic movements, distinct altitudinal migration, etc. 3 = Migratory, i.e. majority of population undertakes long-distance migration)

Trophic.Level (Herbivore = species obtaining at least 70% of food resources from plants; Carnivore = species obtaining at least 70% of food resources by consuming live invertebrate or vertebrate animals; Scavenger = species obtaining at least 70% of food resources from carrion or refuse; Omnivore = species obtaining resources from multiple trophic level in roughly equal proportion,categorical)

Trophic.Niche (Frugivore = species obtaining at least 60% of food resources from fruit; Granivore = species obtaining at least 60% of food resources from seeds or nuts; Nectarivore = species obtaining at least 60% of food resources from nectar; Herbivore = species obtaining at least 60% of food resources from other plant materials in non-aquatic systems, including leaves, buds, whole flowers etc.; Herbivore aquatic = species obtaining at least 60% of food resources from plant materials in aquatic systems, including algae and aquatic plant leaves; Invertivore = species obtaining at least 60% of food resources from invertebrates in terrestrial systems, including insects, worms, arachnids, etc.; Vertivore = species obtaining at least 60% of food resources from vertebrate animals in terrestrial systems, including mammals, birds, reptiles etc.; Aquatic Predator = species obtaining at least 60% of food resources from vertebrate and invertebrate animals in aquatic systems, including fish, crustacea, molluscs, etc; Scavenger = species obtaining at least 60% of food resources from carrion, offal or refuse; Omnivore = Species using multiple niches, within or across trophic levels, in relatively equal proportions

Primary.Lifestyle (Aerial = species spends much of the time in flight, and hunts or forages predominantly on the wing; Terrestrial = species spends majority of its time on the ground, where it obtains food while either walking or hopping (note this includes species that also wade in water with their body raised above the

water); Insessorial = species spends much of the time perching above the ground, either in branches of trees and other vegetation (i.e. arboreal), or on other raised substrates including rocks, buildings, posts, and wires; Aquatic = species spends much of the time sitting on water, and obtains food while afloat or when diving under the water's surface; Generalist = species has no primary lifestyle because it spends time in different lifestyle classes).

To test for differences in ecological traits between habitats (Supplementary Figure 62), we used the Wilcoxon signed rank test for the paired two-sample case to compare the proportional contribution of species with different ecological traits to the set of species detected in urban green spaces vs. at more pristine sites (Supplementary Table 2).

For a substantial number of ecological characteristics, we found significant differences between habitat categories (Supplementary Table 2). Perhaps most importantly, the audible bird communities detected in urban green spaces were more strongly dominated by species associated with human-modified habitats (Supplementary Table 2). Moreover, urban green spaces saw a significantly higher contribution by omnivores (see trait "trophic niche" in Supplementary Figure 62) and by species with a generalist lifestyle (Supplementary Figure 62).

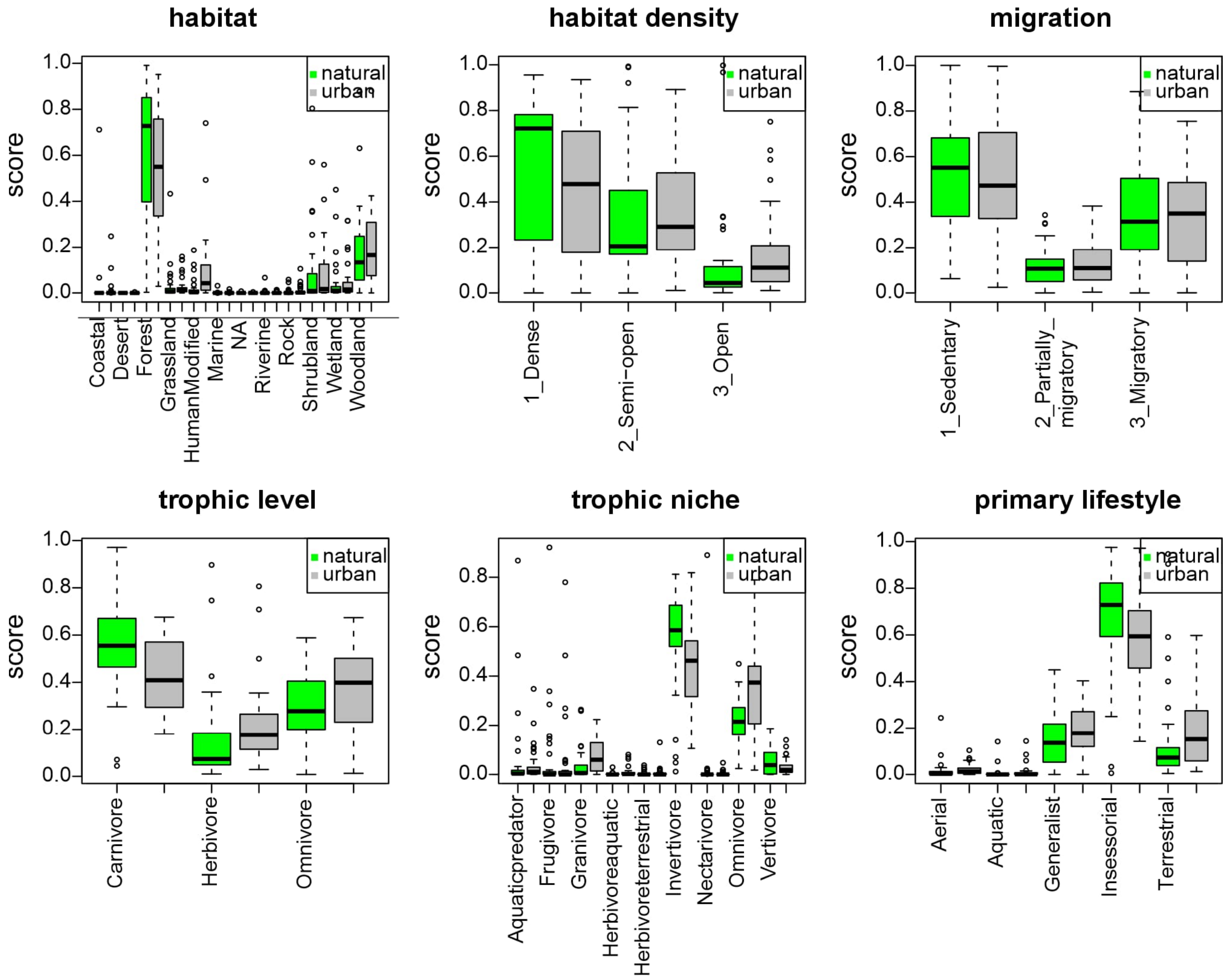

Figure S62. Trait distribution of bird species detected in urban green spaces vs. more pristine habitats, with one panel per trait. Green color is for natural and gray color is for urban.

References

1. Plakal, M. & Ellis, D. YAMNet. *GitHub*, https://github.com/tensorflow/models/tree/master/research/audioset/yamnet (2024)
2. Kahl, S., Wood, C. M., Eibl, M. & Klinck, H. BirdNET: A deep learning solution for avian diversity monitoring. *Ecol Inform* 61, 01236 (2021).
3. Funosas, D. *et al.* Assessing the potential of BirdNET to infer European bird communities from large-scale ecoacoustic data. *Ecol Indic* 164, 112146 (2024).
4. Tobias, J. A. *et al.* AVONET: morphological, ecological and geographical data for all birds. *Ecology Letters* 25, 581–597 (2022).
