# Supplementary material for: Human contributions to global soundscapes are less predictable than the acoustic rhythms of wildlife
Source: Nat Ecol Evol. 2025 Jul 9;9(9):1585–98. doi: 10.1038/s41559-025-02786-5 (PMC12420389; doi:10.1038/s41559-025-02786-5)
Supplement: Supplementary file 2 — Reporting Summary [file 41559_2025_2786_MOESM2_ESM.pdf]

nature portfolio

Corresponding author(s): Otso Ovaskainen

Last updated by author(s): May 16, 2025

Reporting Summary

Nature Portfolio wishes to improve the reproducibility of the work that we publish. This form provides structure for consistency and transparency in reporting. For further information on Nature Portfolio policies, see our Editorial Policies and the Editorial Policy Checklist.

Statistics

For all statistical analyses, confirm that the following items are present in the figure legend, table legend, main text, or Methods section.

n/a	Confirmed	
☐	☒	The exact sample size (n) for each experimental group/condition, given as a discrete number and unit of measurement
☐	☒	A statement on whether measurements were taken from distinct samples or whether the same sample was measured repeatedly
☐	☒	The statistical test(s) used AND whether they are one- or two-sided<br>*Only common tests should be described solely by name; describe more complex techniques in the Methods section.*
☐	☒	A description of all covariates tested
☐	☒	A description of any assumptions or corrections, such as tests of normality and adjustment for multiple comparisons
☐	☒	A full description of the statistical parameters including central tendency (e.g. means) or other basic estimates (e.g. regression coefficient) AND variation (e.g. standard deviation) or associated estimates of uncertainty (e.g. confidence intervals)
☐	☒	For null hypothesis testing, the test statistic (e.g. F, t, r) with confidence intervals, effect sizes, degrees of freedom and P value noted<br>*Give P values as exact values whenever suitable.*
☒	☐	For Bayesian analysis, information on the choice of priors and Markov chain Monte Carlo settings
☐	☒	For hierarchical and complex designs, identification of the appropriate level for tests and full reporting of outcomes
☐	☒	Estimates of effect sizes (e.g. Cohen's d, Pearson's r), indicating how they were calculated

Our web collection on statistics for biologists contains articles on many of the points above.

Software and code

Policy information about availability of computer code

Data collection	Custom AudioMoth firmware installed on the audio recorders used to record audio: https://github.com/OpenAcousticDevices/AudioMoth-LIFEPLAN/releases/download/0.1.5/AudioMoth-LIFEPLAN-0.1.5.bin<br>Full audio recording protocol, including the two AudioMoth configurations used to record audio: https://dx.doi.org/10.17504/protocols.io.kqdg3xbp1g25/v2
Data analysis	The scripts and code to reproduce the results of this manuscript are found in GitHub repository https://github.com/psomervuo/soundscape.<br>Bird species identifications: BirdNet Analyzer version 2.4. (Kahl, S., Wood, C. M., Eibl, M. & Klinck, H. BirdNET: A deep learning solution for avian diversity monitoring. Ecol Inform 61, 101236 (2021))<br>Spectrogram computing: Python library librosa version 0.9.2.33<br>Acoustic indices computing: Python package Acoustic_Indices v1.0.0

For manuscripts utilizing custom algorithms or software that are central to the research but not yet described in published literature, software must be made available to editors and reviewers. We strongly encourage code deposition in a community repository (e.g. GitHub). See the Nature Portfolio guidelines for submitting code & software for further information.

Data

Policy information about availability of data

All manuscripts must include a data availability statement. This statement should provide the following information, where applicable:

- Accession codes, unique identifiers, or web links for publicly available datasets
- A description of any restrictions on data availability
- For clinical datasets or third party data, please ensure that the statement adheres to our policy

The data have been deposited to Zenodo https://doi.org/10.5281/zenodo.11516373. The repository will be opened upon acceptance of the manuscript. A link for reviewers is included in the manuscript text.

Research involving human participants, their data, or biological material

Policy information about studies with human participants or human data. See also policy information about sex, gender (identity/presentation), and sexual orientation and race, ethnicity and racism.

Reporting on sex and gender	This information has not been collected.
Reporting on race, ethnicity, or other socially relevant groupings	This information has not been collected.
Population characteristics	Research does not involve humans or human data.
Recruitment	Research does not involve humans or human data.
Ethics oversight	Research does not involve humans or human data.

Note that full information on the approval of the study protocol must also be provided in the manuscript.

Field-specific reporting

Please select the one below that is the best fit for your research. If you are not sure, read the appropriate sections before making your selection.

☐ Life sciences ☐ Behavioural & social sciences ☒ Ecological, evolutionary & environmental sciences

For a reference copy of the document with all sections, see nature.com/documents/nr-reporting-summary-flat.pdf

Ecological, evolutionary & environmental sciences study design

All studies must disclose on these points even when the disclosure is negative.

Study description	At each of 139 sites around the world, we deployed 3-5 passive audio recorders in a nested design. Some sites in the Nordic countries and Madagascar were sampled continuously, while others switched annually between a natural and urban type location as proposed by local researchers. In this study, we used a randomly selected one-minute clip from each hour recorded. We used a total of 1,484,181 clips. For these clips, we calculated a set of established acoustic indices and acoustic event classes, and identified bird species vocalising. We analysed periodic rhythms in different soundscape indices, evaluated the predictability of local patterns from global ones as well as anthropogenic and climatic impacts, and effects of natural/urban site type on acoustic index values and species richness. Explanatory variables used included day of year, time of day, latitude, human footprint index, elevation, annual mean temperature, annual precipitation and habitat type. Full study description: https://doi.org/10.1371/journal.pone.0313353
Research sample	In the statistical analyses we use as the sampling unit one minute of sound. The spatiotemporal distrubution of these sampling units is described in the study description.
Sampling strategy	At each site, data were collected via passive acoustic monitoring (PAM) using AudioMoth v1.1 devices63. For any one time, there were up to five AudioMoth devices operated per site within a 1-ha area62. The total number of recordings varied between the sites due to equipment malfunctioning and logistic constraints, such as site accessibility due to road damage caused by hurricanes and storms. At some sites, the sampling period included only a few months, whereas in other sites the sampling was continuous throughout the year. The data were subsampled to the level of full hours. To allow the sampling of equivalent time periods, we ensured synchronisation among individual recorders. To this aim, all AudioMoth devices were synchronized weekly with Coordinated Universal Time. From each hour recorded, a one-minute-long clip was randomly selected. Consequently, the data to be analysed consisted of up to 24 recordings per day per site. The total number of the one-minute recordings used in the present analysis was 1,484,181.
Data collection	Sampling teams around the world placed 3-5 pre-programmed passive audio recorders at their sites. They visited them weekly or biweekly to change batteries and microSD cards. They collected metadata by scanning QR codes on the recorders and microSD cards, recording the time and location of each recorder placement and collection.

Timing and spatial scale	Audio recording began at the first site 2020-12-21 and is still ongoing. The data included in this study range from 2021-02-08 to 2024-04-18. Audio was recorded for one minute every ten minutes on site corners, and continuously for 48 hours at the start of each week and then one minute every ten minutes in the middle of the site. The recording regime was designed so that one visit per week would be enough to keep batteries running and memory cards from filling up. Our spatial scale is global, with 139 sites on six continents and a latitudinal gradient of 116 degrees. A full list of sites is in supplementary table 3. There are gaps in the recording schedule due to equipment failure and replacement delays.
Data exclusions	We excluded only sites that had too little sampling for statistical analysis. From each hour recorded, a one-minute-long clip was randomly selected.
Reproducibility	To verify species identifications, an ornithologist listened to a random sample of recordings to check the species identifications. Each site had 3-5 audio recorders whose results can be compared.
Randomization	We randomised sites as to starting at the Natural or Urban location. We randomised the choice of a one-minute clip from each hour of recording.
Blinding	Blinding was not applied because the data were analysed with automated scripts without subjective human judgment. Audio files were encrypted and not listened to or filtered by data collectors.

Did the study involve field work? ☒ Yes ☐ No

Field work, collection and transport

Field conditions	Field work was carried out over several years at 139 locations on six continents around the world, and field conditions varied considerably.
Location	Full list of 139 study sites is in supplementary table S3.
Access & import/export	Data was collected and is owned by participating teams. Teams were instructed to follow their local legislation concerning audio recording and to post signs warning that audio is being recorded.
Disturbance	Audio recorders were attached with canvas straps to trees where available. Where trees were not available, teams installed posts in the ground. The recorders were visited once a week.

Reporting for specific materials, systems and methods

We require information from authors about some types of materials, experimental systems and methods used in many studies. Here, indicate whether each material, system or method listed is relevant to your study. If you are not sure if a list item applies to your research, read the appropriate section before selecting a response.

Materials & experimental systems

n/a	Involved in the study	
☒	☐	Antibodies
☒	☐	Eukaryotic cell lines
☒	☐	Palaeontology and archaeology
☐	☒	Animals and other organisms
☒	☐	Clinical data
☒	☐	Dual use research of concern
☒	☐	Plants

Methods

n/a	Involved in the study	
☒	☐	ChIP-seq
☒	☐	Flow cytometry
☒	☐	MRI-based neuroimaging

Animals and other research organisms

Policy information about studies involving animals; ARRIVE guidelines recommended for reporting animal research, and Sex and Gender in Research

Laboratory animals	Not used
Wild animals	Wild animal sounds were passively recorded. No animals were captured or manipulated. Animals identified from the recordings were birds.
Reporting on sex	Sex was not considered or reported on.
Field-collected samples	No physical samples were collected.

Ethics oversight	No ethical approval or guidance was required, as no animals were handled in the study.

Note that full information on the approval of the study protocol must also be provided in the manuscript.

Plants

Seed stocks	Not used.
Novel plant genotypes	Not used.
Authentication	Not used.
